# Silane Redistribution Catalyzed by [Mes-B-TMP]^+^ Borinium Ion

**DOI:** 10.1021/acs.inorgchem.5c05711

**Published:** 2026-01-21

**Authors:** Min-Hsiung Wu, Yu-Jiang Lin, Bo-An Chen, Ching-Wen Chiu

**Affiliations:** Department of Chemistry, 33561 National Taiwan University, No. 1, Section 4, Roosevelt Road, Taipei 10617, Taiwan

## Abstract

Borinium ions, featuring two empty p orbitals at boron, exhibit exceptional Lewis acidity but often suffer from excessive reactivity that limits their catalytic utility. In this study, we demonstrate that the arylamino borinium ion [**1**]^+^ effectively mediates aryl transfer in ArMe_2_SiH to afford Ar_2_SiMe_2_ and Me_2_SiH_2_ under mild conditions. The reaction can also be extended to cross-coupling between ArMe_2_SiH and Et_2_SiH_2_/Et_3_SiH. Computational studies revealed that the amino substituent in [**1**]^+^ plays a pivotal role in modulating the Lewis acidity of the boron center, enabling reversible Si–H activation while preventing irreversible B–C bond cleavage and catalyst decomposition. These findings highlight the potential of electronically tuned borinium ions as catalysts for activating inert substrates in organic transformations.

## Graphical abstract

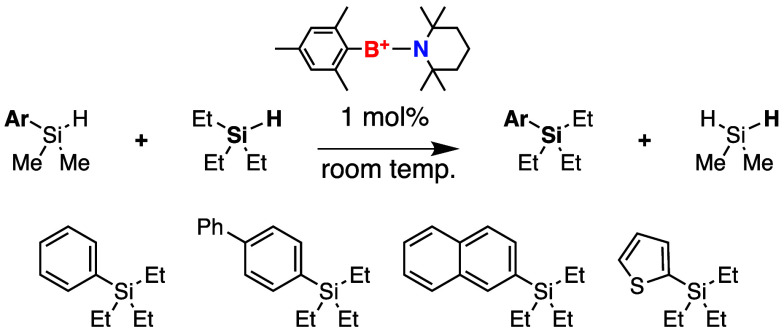

Silanes are indispensable reagents in organic synthesis, material science, polymer chemistry, and semiconductor manufacturing.^1–4^ While organometallic reagents are commonly employed for the construction of Si–C bonds, silane redistribution, a process involving the exchange of substituents between silicon centers, offers an atom-economical route to generate diverse organosilanes (Figure 1a).^5,6^ Catalytic silane redistribution was first documented in the mid-19th century, when Friedel and Ladenburg observed the disproportionation of triethoxysilane (SiH­(OEt)_3_) to silane (SiH_4_) and tetraethoxysilane (Si­(OEt)_4_) in the presence of sodium, a process likely mediated by the ethoxide anion.^7^ Since then, silane redistribution utilizing alkaline catalysts has been investigated in detail.^8–10^ Later, acidic metal halides were found to catalyze the redistribution of tetraalkylsilanes at elevated temperature with a catalytic order of Al_2_X_6_ > Ga_2_X_6_ > FeX_3_ ≈ BX_3_, which is consistent with their Lewis acidity as observed in electrophilic aromatic substitution reactions.^11^ In addition, transition-metal complexes have been shown to promote silane redistribution.^6,12–15^ However, high reaction temperatures are generally required for these systems. In 2021, our group reported the synthesis of an arylamino borinium ion, [Mes-B-TMP]­[B­(C_6_F_5_)_4_] ([**1**]­[B­(C_6_F_5_)_4_]), and demonstrated its catalytic activity in hydrosilylation of ketones and aldehydes.^16,17^ We proposed that the key activation step involves the interaction of [**1**]^+^ with the Si–H bond of silane rather than with the carbonyl compounds. This finding prompted us to explore the potential of [**1**]^+^ as a Lewis acid catalyst for silane redistribution at ambient temperature (Figure 1b).

**Figure 1:**
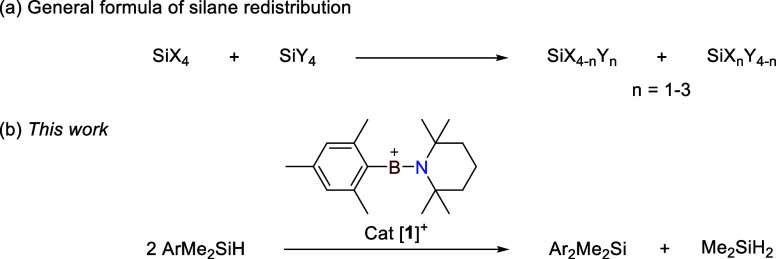
(a) General formula of silane redistribution. (b) [**1**]^+^-catalyzed aryl transfer between silanes.

In 2017, Hou and co-workers demonstrated that B­(C_6_F_5_)_3_ (BCF), a prototypical strong boron Lewis acid, can catalyze the C–Si/Si–H cross-metathesis of electron-rich hydrosilanes.^18^ This method, however, required harsh conditions (100 °C for 24 h) and was limited to substrates possessing an *m*-aminoaryl group. Unfunctionalized substrates, such as PhMe_2_SiH (**2a**), remained intact even after heating at 100 °C for 24 h. In stark contrast, simply treating a CDCl_3_ solution of **2a** with 10 mol% of [**1**]­[B­(C_6_F_5_)_4_] at room temperature in a sealed J-Young tube rapidly afforded the redistribution product, Ph_2_Me_2_Si (**3a**) and Me_2_SiH_2_ (**4**), in 43% yield. However, the borinium ion was not effective for Ph_2_SiH_2_, Et_2_SiH_2_, or Et_3_SiH (Table S1). The reaction of Ph_2_SiH_2_ produced a complex mixture of silanes containing multiple Ph_*x*_SiH_4‑*x*_ (*x* = 0–4) derivatives, indicating extensive and uncontrolled redistribution. In contrast, Et_2_SiH_2_ and Et_3_SiH remained largely unreacted under identical conditions, and no significant redistribution products were detected. Thus, we decided to focus on the redistribution of aryldimethylsilanes (ArMe_2_SiH).

To improve the yield and selectivity of the redistribution of **2a**, we screened several solvents (Table 1). Despite the complete consumption of **2a** in CDCl_3_ or CH_2_Cl_2_ upon the addition of [**1**]^+^, **3a** was obtained only in moderate yields. Analysis of the reaction mixture revealed the formation of chlorosilane byproducts (e.g., PhMe_2_SiCl), suggesting the participation of the solvent in the reaction. This side reaction is consistent with the generation of silylium ion intermediates through heterolytic cleavage of the Si–H bond.^19,20^ To suppress chlorosilane formation, the reaction was carried out in aromatic solvents. All aromatic solvents provided comparable results, with chlorobenzene (PhCl) being marginally superior. We noticed that, despite the poor solubility of [**1**]^+^ in benzene, the catalytic performance remained comparable to that observed in halobenzene solvents. This observation suggested that the catalyst loading could be reduced without compromising reactivity. Indeed, a comparable yield of **3a** was achieved within 20 min using only 1 mol% catalyst. Finally, both conversion and yield were further improved by conducting the reaction in an open vial inside a glovebox to continuously remove the volatile byproduct Me_2_SiH_2_ (**4**), thereby shifting the equilibrium toward the products (Table 1).^21,22^

**Table 1:**
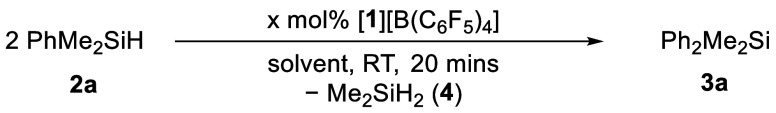
Optimization of Silane Redistribution Catalyzed by [**1**]­[B­(C_6_F_5_)_4_]

Entry	*x*	Solvent	Conversion (%)^a^	Yield (%)^a^
1	10	CDCl_3_	99	43
2	10	CH_2_Cl_2_	99	42
3	10	C_6_D_6_	60	55
4	10	*o*-C_6_D_4_Cl_2_	60	58
5	10	C_6_H_5_Cl	66	64
6	5	C_6_H_5_Cl	65	63
7	1	C_6_H_5_Cl	65	62
8^b^	1	C_6_H_5_Cl	90	84

a Conversion and yield were determined by ^1^H NMR spectroscopy.

b The reaction was stirred in an opened vial in glovebox for 1 h.

With the optimal conditions established, we next explored the substrate scope of the reaction across a series of functionalized arylsilanes (Figure 2). The *ortho*-tolyl (**2b**), *para*-bromophenyl (**2d**), biphenyl-4-yl (**2e**), 2-naphthyl (**2i**), and 2-thiophenyl (**2j**) silanes were efficiently converted to the corresponding diaryl­(dimethyl)­silanes in high yields. In contrast, the *para*-tolyl-substituted silane (**2c**) afforded **3c** in only a moderate yield. Examination of the reaction mixture revealed the presence of a 1:1 ratio of (p-tol)_3_SiMe (**5**) and (p-tol)­SiMe_3_ (**6**), indicating the occurrence of arene/Me exchange. Indeed, monitoring of the reaction progress by NMR spectroscopy confirmed the rapid generation of **3c** in a high yield within the first 5 min (Figure S2). However, the yield of **3c** subsequently declined from 80% to 50%, reaching equilibrium at 20 min with the concomitant formation of **5** and **6**. The ability of [**1**]^+^ to activate the Si–Me bond of **3c** was further confirmed by the formation of **5** and **6** (11% each) from **3c** in the presence of 1 mol% of [**1**]^+^ (Figure S3). Unfortunately, the reaction was not applicable to substrates bearing trifluoromethyl (**2f**), dimethylamino (**2g**), or methoxy (**2h**) substituents. For **2g**, catalyst inhibition via base coordination was observed, whereas activation of the CF_3_ group in **2f** led to the formation of a fluorosilane mixture (Figures S4–S6). In the case of **2h**, polymerization via a Piers–Rubinsztajn-type reaction, accompanied by methane evolution and Si–O bond formation, was observed (Figure S7).^23,24^

**Figure 2:**
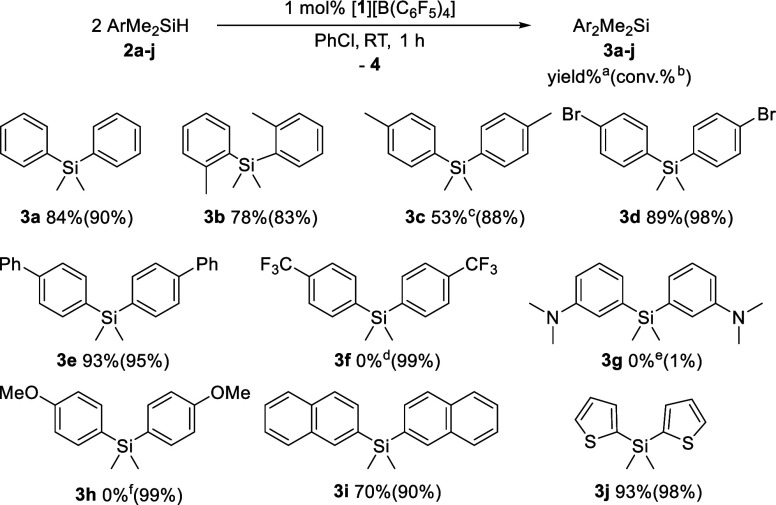
Catalytic redistribution of **2a**–**j** to afford **3a**–**j**. ^a^Yield is based on 0.5 equiv of substrate and determined by ^1^H NMR spectroscopy. ^b^Conversion of **2a**–**j** is determined by ^1^H NMR spectroscopy. ^c^Byproducts **5** and **6** were observed. ^d^Defluorination was observed. ^e^Catalyst was coordinated by aniline. ^f^Piers–Rubinsztajn reaction was observed.

After completing the homometathesis study of aryl­dimethyl­silanes, we next examined whether cross-coupling reactions between two different arylsilanes could be achieved (Table S2). Treating an equimolar mixture of **2a** and **2d** with 1 mol% [**1**]^+^ afforded a mixture containing **3a**, (4-Br-C_6_H_4_)­PhMe_2_Si (**7d**), and **3d** in 17%, 15%, and 1% NMR yield, respectively. Increasing the catalyst loading to 3 mol% markedly improved the yield of the cross-coupling product **7d** to 54%. Attempts to enhance cross-coupling by increasing the steric bulk of the aryl group were unsuccessful. The reaction of **2a** and **2b** resulted in the formation of (*o*-tolyl)­PhMe_2_Si (**7b**) in at most 36% yield. Notably, when **2a** was reacted with the more electron-donating thiophenylsilane (**2j**), the cross-coupling product (2-thio­phenyl)­PhMe_2_Si (**7j**) was formed in a 74% yield. In all cases, however, substantial amounts of homocoupling products were also obtained, due to the comparable donating abilities of the aryldimethylsilane substrates. Since tuning the electron density of the aryl ring through CF_3_, OMe, or NMe_2_ substitution was not feasible, we next explored the cross-coupling reactions of aryldimethylsilanes with Et_2_SiH_2_ and Et_3_SiH.

To our delight, the cross-coupling products **8** and **9** were obtained in high yields by mixing aryldimethylsilanes with alkylsilanes in the presence of 1 mol% of [**1**]^+^ at room temperature (Figure 3). The reaction of **2a** with 4 equiv of Et_2_SiH_2_ afforded the cross-coupling product **8a** in 73% yield, whereas mixing **2a** and 2 equiv of Et_3_SiH gave **9a** in 90% yield. Because compound **8**, ArEt_2_SiH, can undergo further aryl transfer reaction in the presence of a borinium ion catalyst, a higher loading of Et_2_SiH_2_ was essential to suppress secondary transformation. The reaction was successfully extended to other combinations of silanes, furnishing the corresponding cross-coupling products in high yields, including 2-tolyl­diethyl­silane (**8b**), 4-tolyl­diethyl­silane (**8c**), 4-bromo­phenyl­diethyl­silane (**8d**), biphenyl-4‑yl­diethyl­silane (**8e**), biphenyl-4‑yl­triethyl­silane (**9e**), 2-naphthyl­diethyl­silane (**8i**), 2-naphthyl­triethyl­silane (**9i**), 2-thio­phenyl­diethyl­silane (**8j**), and 2-thio­phenyl­triethyl­silane (**9j**).

**Figure 3:**
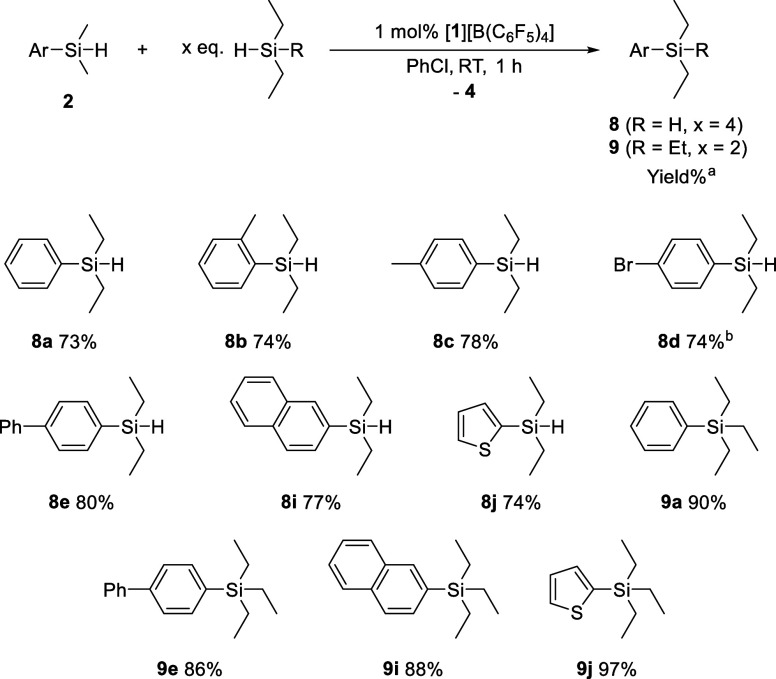
Cross-coupling of **2** with Et_2_SiH_2_ or Et_3_SiH. ^a^Yields were determined by ^1^H NMR spectroscopy. ^b^The reaction time was prolonged to 12 h.

To gain deeper insight into the aryl amino borinium-mediated aryl transfer process, we examined the redistribution reaction of PhMe_2_SiH computationally. Three potential reaction pathways between PhMe_2_SiH and [**1**]^+^ were identified, and all were found to be endergonic (Figure 4a). Compared to the Si–H addition across the B–N and B–C bonds, the formation of a silane-borinium σ-complex represents the most feasible initial interaction for [**1**]^+^ with an energy barrier of 13.58 kcal/mol.^25,26^ To elucidate the influence of the amino substituent at boron, we also evaluated the corresponding processes for the more Lewis acidic all-carbon-substituted borinium ion [Mes_2_B]^+^ (Figure 4b).^27,28^ Both the 1,2-addition pathway and the σ-complex formation at [Mes_2_B]^+^ were found to be exergonic with noticeably lower activation barriers, which are consistent with the higher intrinsic Lewis acidity of [Mes_2_B]^+^. However, the increased reactivity of [Mes_2_B]^+^ toward B–C bond cleavage, plausibly happening after the Si–H activation, would destroy the borinium scaffold and prevent catalytic turnover. This prediction aligns with experimental observations: while [**1**]^+^ serves as an efficient catalyst for silane redistribution, [Mes_2_B]^+^ decomposes upon addition of silane (Table S4).^29^ The amine substituent in [**1**]^+^ plays a key role in modulating the Lewis acidity of the boron center, thereby enabling reversible Si–H activation.

**Figure 4:**
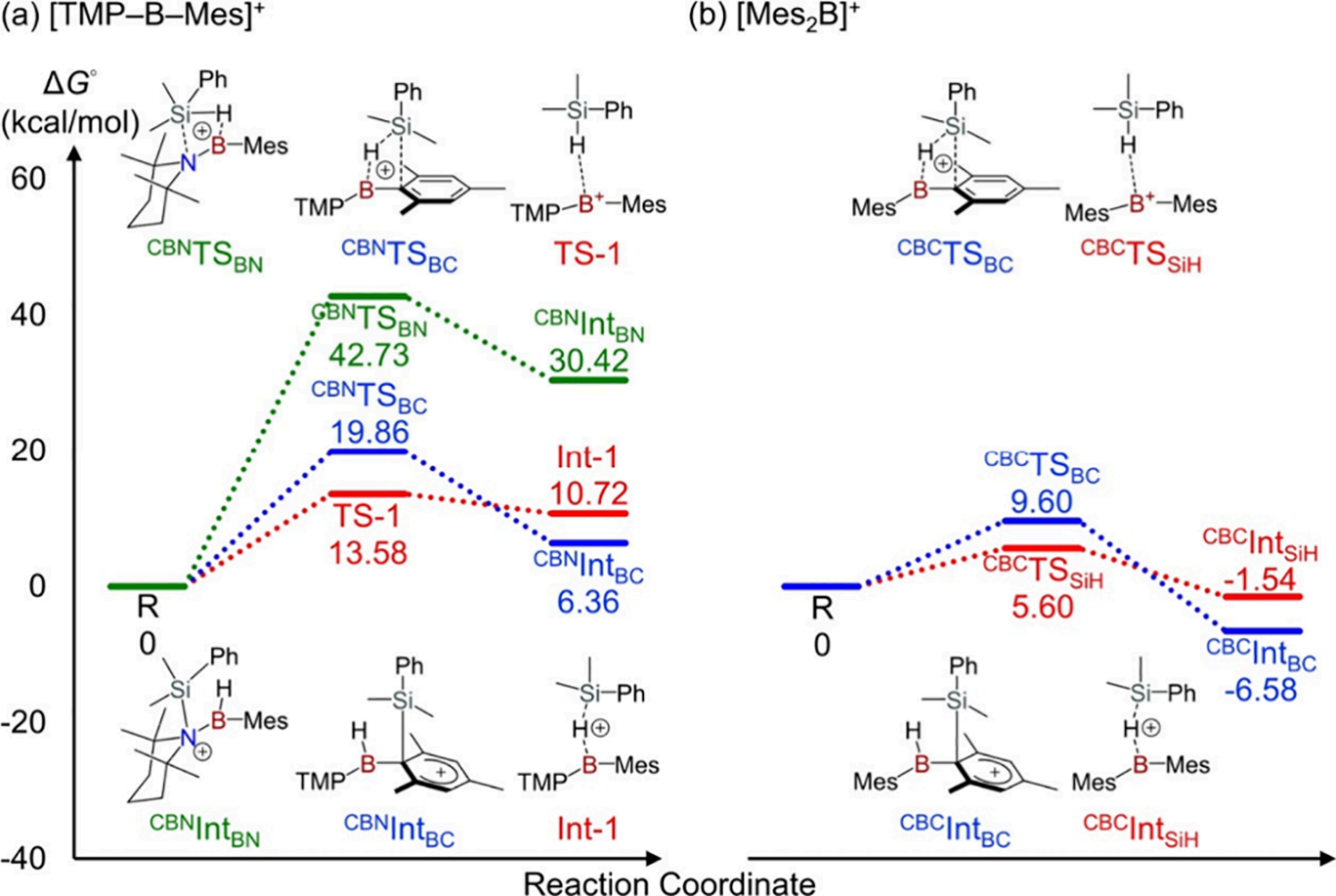
Calculated activation modes of PhMe_2_SiH by (a) [**1**]^+^ and (b) [Mes_2_B]^+^.

A proposed reaction mechanism and the calculated potential energy surface for the [**1**]^+^ catalyzed redistribution of PhMe_2_SiH is shown in Figure 5. The initial step is the formation of a silane-borinium σ-complex (**Int-1**) as discussed above. The second step involves nucleophilic attack by the phenyl group of a second PhMe_2_SiH molecule on the silicon atom of **Int-1**, inducing heterolytic Si–H cleavage to generate the phenyl-bridged disilicon cation (**Int-2**) and hydridoborane (**1-H**).^30^ Subsequent reverse hydride transfer from **1-H** to the less sterically hindered silicon center of **Int-2** yields Ph_2_SiMe_2_ and the Me_2_SiH_2_-borinium σ-complex (**Int-3**). The calculated energy differences among **Int-1**, **Int-2** + **1-H**, and **Int-3** are small, and the highest barrier in this sequence is only 14.71 kcal/mol, indicating that these species exist in a rapid and reversible equilibrium. Therefore, removal of the volatile Me_2_SiH_2_ is essential to drive the reaction toward completion. Additionally, the calculated overall energy barrier of the reaction is 25.43 kcal/mol, which is consistent with our experimental results. The calculated reaction mechanism is comparable to the BCF-mediated aryl transfer reaction proposed by Hou’s group.^18^

**Figure 5:**
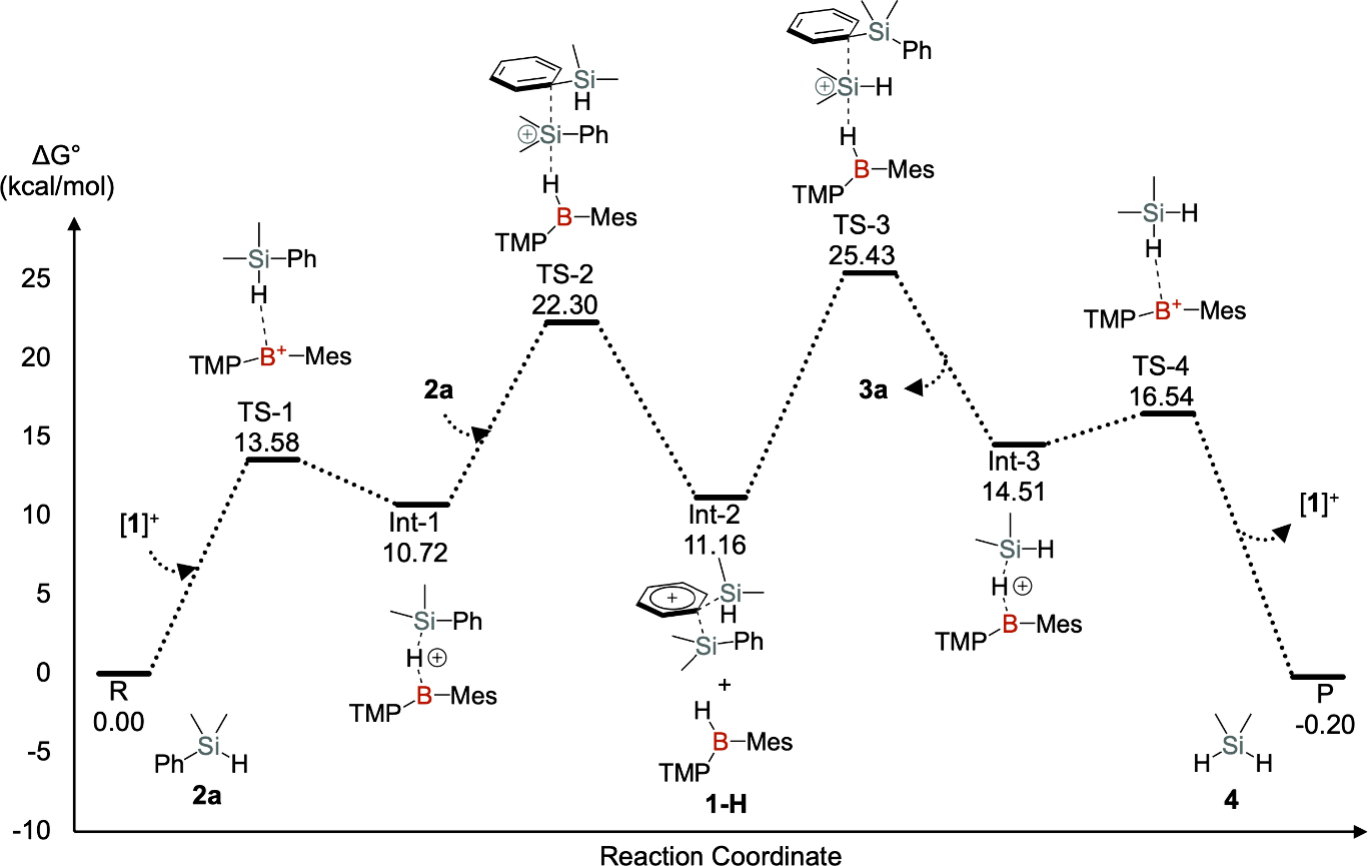
Proposed reaction mechanism of the [**1**]^+^-catalyzed redistribution of PhMe_2_SiH.

In conclusion, we have demonstrated that the arylamino borinium ion [**1**]^+^ is an effective catalyst for the redistribution of unfunctionalized ArMe_2_SiH, affording Ar_2_SiMe_2_ and Me_2_SiH_2_ at ambient temperature. The aryl transfer process between silanes can be extended to diethyl- and triethylsilanes, providing the corresponding cross-coupling products in high yields. Theoretical calculations reveal that the amino substituent in the borinium ion is essential for achieving reversible Si–H activation while preventing catalyst decomposition. Overall, this study not only demonstrates the capacity of borinium ions to active inert substrates but also highlights the critical role of the borinium substituent in determining catalyst performance.

## Supplementary Material





## Notes

Synthesis and characterization of silane substrates and redistribution products, and computational details (PDF)
Optimized geometries (XYZ)
