# Supplementary material for: Silane Redistribution Catalyzed by [Mes-B-TMP]+ Borinium Ion
Source: Inorg Chem. 2026 Jan 21;65(4):2117–21. doi: 10.1021/acs.inorgchem.5c05711 (PMC12869485; doi:10.1021/acs.inorgchem.5c05711)
Supplement: Supplementary file 1 [file ic5c05711_si_001.pdf]

Supporting Information

For

Silane Redistribution Catalyzed by [Mes-B-TMP]$^+$ Borinium Ion

*Min-Hsiung Wu, Yu-Jiang Lin, Bo-An Chen, Ching-Wen Chiu**

Department of Chemistry, National Taiwan University, No. 1, Section 4, Roosevelt Road, Taipei, Taiwan, 10617

Contants

1. General Considerations.. S3

2. Experimental Details S4

3. Synthetic Details.. S13

4. Computational Details S25

5. NMR Spectra S28

6. References S70

1. General Considerations

All the reactions are carried out by using Schlenk system or glovebox under nitrogen or argon atmosphere. CH_2Cl_2, Et_2O and THF were purified by the solvent purification system. Silanes, C_6H_5Cl, *o*-$C_6D_4Cl_2$, CD_2Cl_2, and $CDCl_3$ were dried by P_2O_5 or 4Å molecular sieves and distilled under nitrogen. Mesitylene, benzene, and hexane were dried by Na/K alloy and distilled under nitrogen. Bromoarenes, n-BuLi solution, Me_2SiHCl, and $PhMe_2SiH$ were purchased and used without further purification. NMR spectra were collected by using Bruker Avance III-500 (^{1}H: 500.2 MHz, ^{11}B: 128.4 MHz, ^{13}C: 125.7 MHz, ^{19}F: 376 MHz, ^{29}Si: 99 MHz).

Caution: *Silanes are flammable and moisture-sensitive and should be handled under an inert atmosphere.*

2. Experimental Details

2.1 Optimization of substrate

Table S1. Optimization of Silane Redistribution catalyzed by [1][B(C_6F_5)$_4$].

$$\text{Substrate} \xrightarrow[\text{CDCl}_3,\ \text{RT}]{10\ \text{mol\% } [\mathbf{1}][\text{B}(\text{C}_6\text{F}_5)_4]} \text{Product}$$

Entry	Substrate	T (h)	Product (yield %)[a]
1	$PhMe_2SiH$ (**2a**)	0.5	Ph_2Me_2Si (43) + Me_2SiH_2
2	Ph_2SiH_2 (**2b**)	0.5	Ph_4Si (14) + Ph_3SiH (11) + Ph_2SiH_2 (18) + $PhSiH_3$ (24) + SiH_4 (30)
3	Et_2SiH_2 (**2c**)	24	Et_3SiH (trace) + $EtSiH_3$ (trace)

[a] Yields were determined by ^{1}H NMR spectroscopy.

2.2 Catalytic redistribution of $PhMe_2SiH$ in a sealed J-Young tube

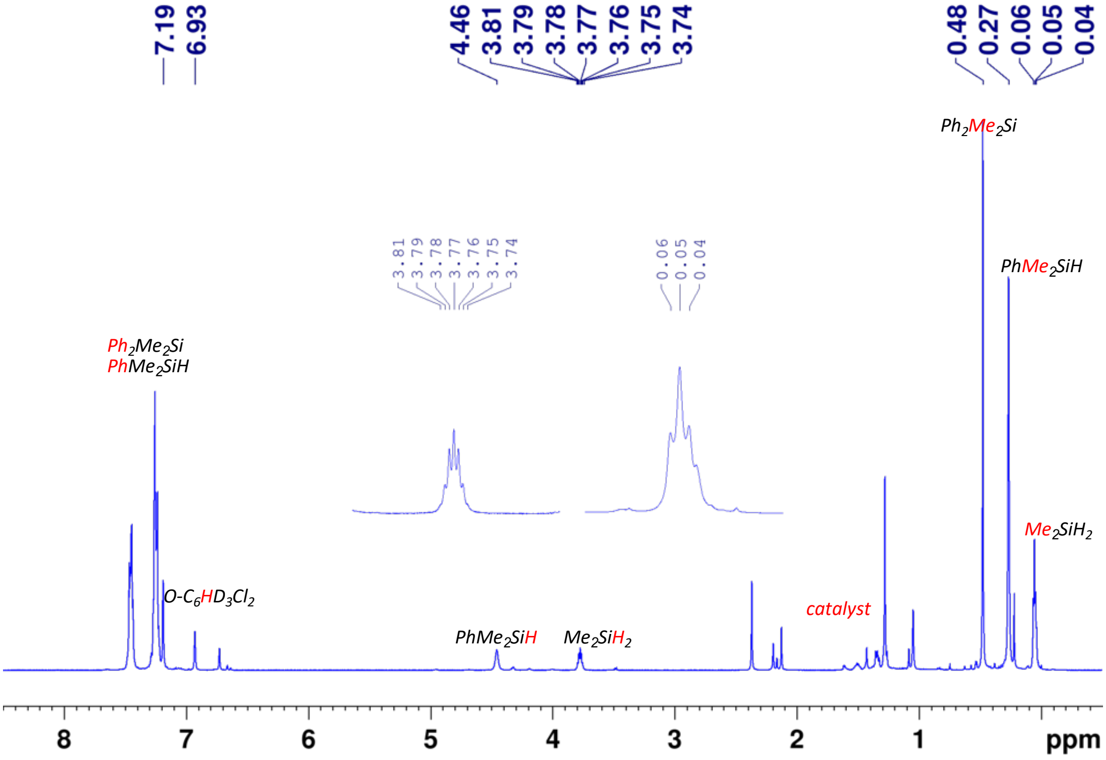

Figure S1. Crude ^{1}H NMR spectrum (400 MHz) of the catalytic redistribution of $PhMe_2SiH$ to Ph_2SiMe_2 and Me_2SiH_2 in *o*-$C_6D_4Cl_2$.

2.2 Redistribution of (4-tolyl)Me_2SiH

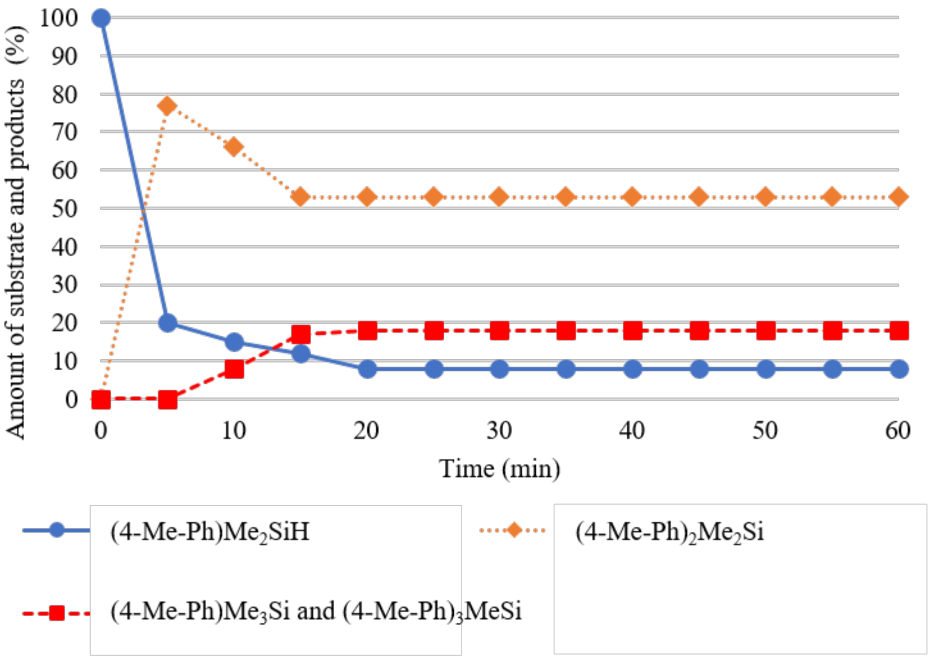

Figure S2. **Dependence of product of 2c after adding 1 mol% of [1][$B(C_6F_5)_4$] on time**

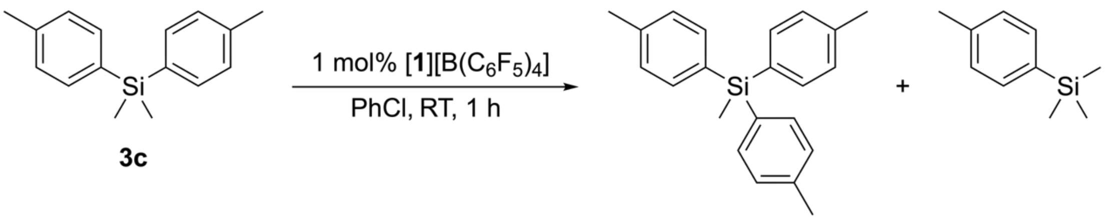

Figure S3. **Redistribution of 3c catalyzed by [1][$B(C_6F_5)_4$].**

2.3 Hydro-defluorination of (4-CF_3-C_6H_4)Me_2SiH

As shown in **Figure S4**, the signals of **2f** (4.34 - 4.30 ppm, 0.12 ppm) disappear upon the addition of catalyst, and new signals at 0.16 ppm and -0.08 ppm were observed in the ^{1}H NMR spectrum of the reaction mixture. The emerged signal centered at 0.16 ppm is assigned to the methyl groups of (4-CF_3-C_6H_4)**Me_2**SiF, while the -0.08 ppm signal is attributed to the methyl groups of **Me_2**SiF_2. The formation of Si-F bonds is further corroborated with ^{19}F NMR. As shown in **Figure S4**, the ^{19}F NMR spectrum of the reaction mixture consisting of **2f** and 1 mol% of [**1**]$^+$ features new signals at -131.98 ppm and -163.32 ppm. As the former signal is the fluorine signal of Me_2Si**F_2**, the latter one belongs to (4-CF_3-C_6H_4)Me_2Si**F**. In the proton-coupled ^{19}F NMR spectrum (**Figure S5**), the observation of two septet at -131.98 ppm and -163.32 ppm with $^3J_{H\text{-}F}$ coupling constants of 6.20 Hz (-131.98 ppm) and 7.48 Hz (-163.32 ppm) are consistent with that reported for Me_2SiF_2 and $PhMe_2SiF$.[1] However, signals of (4-CHF_2-C_6H_4)Me_2SiF or (4-CH_2F-C_6H_4)Me_2SiF were not observed.

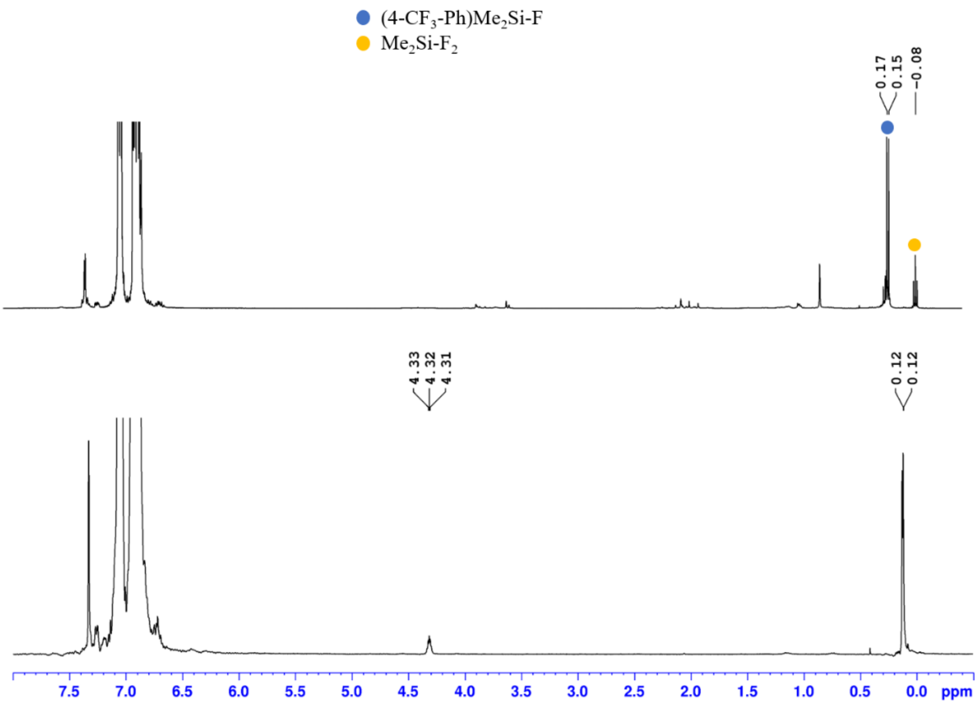

Figure S4. ^{1}H NMR spectra of 2f with 1 mol% of [1][B(C_6F_5)$_4$] (up) in comparison with 2f (down) in PhCl solution.

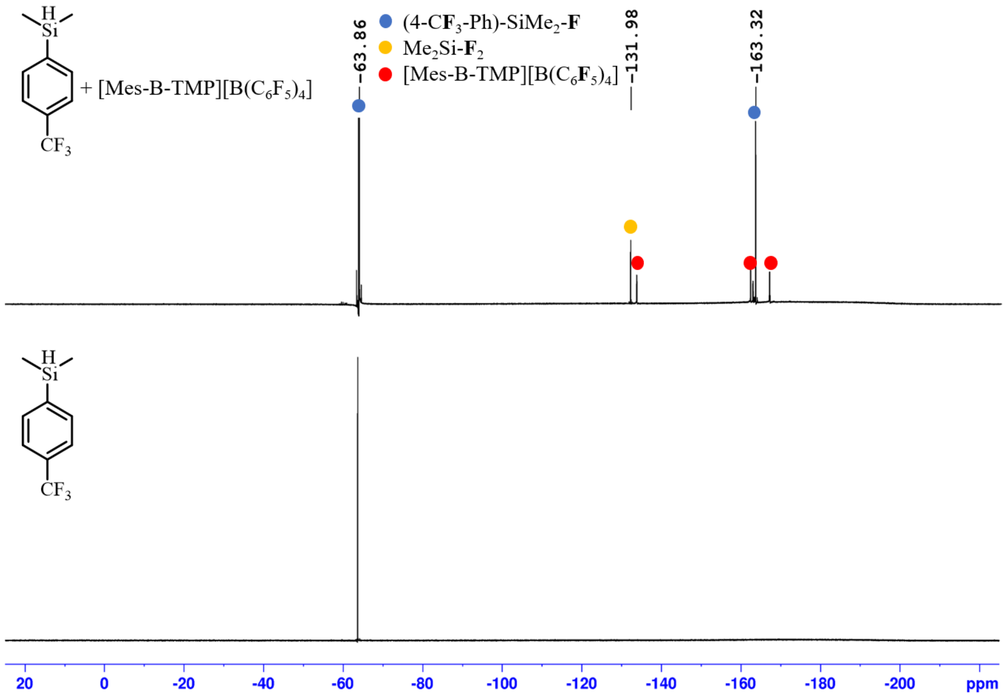

Figure S5. ^{19}F NMR spectra of 2f with 1 mol% of [1][B(C_6F_5)$_4$] (Up) in comparison with 2f (Down) in PhCl solution.

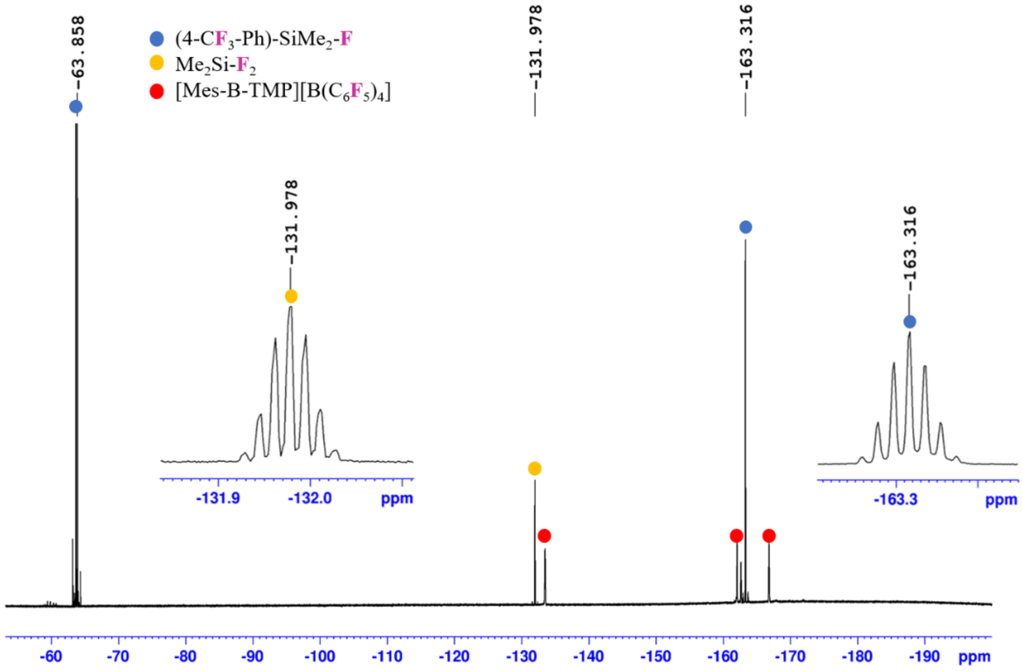

Figure S6. ^{19}F NMR spectrum of 2f with 1 mol% of [1][B(C_6F_5)$_4$] in PhCl solution.

2.4 Polymerization of (4-MeO-C_6H_4)Me_2SiH

As shown in **Figure S7**, the signal of CH_3O- and Si-**H** disappeared when **2h** was treated with [**1**]$^+$. Besides, the singlet appeared at 0.13 ppm is comparable to that of CH_4.

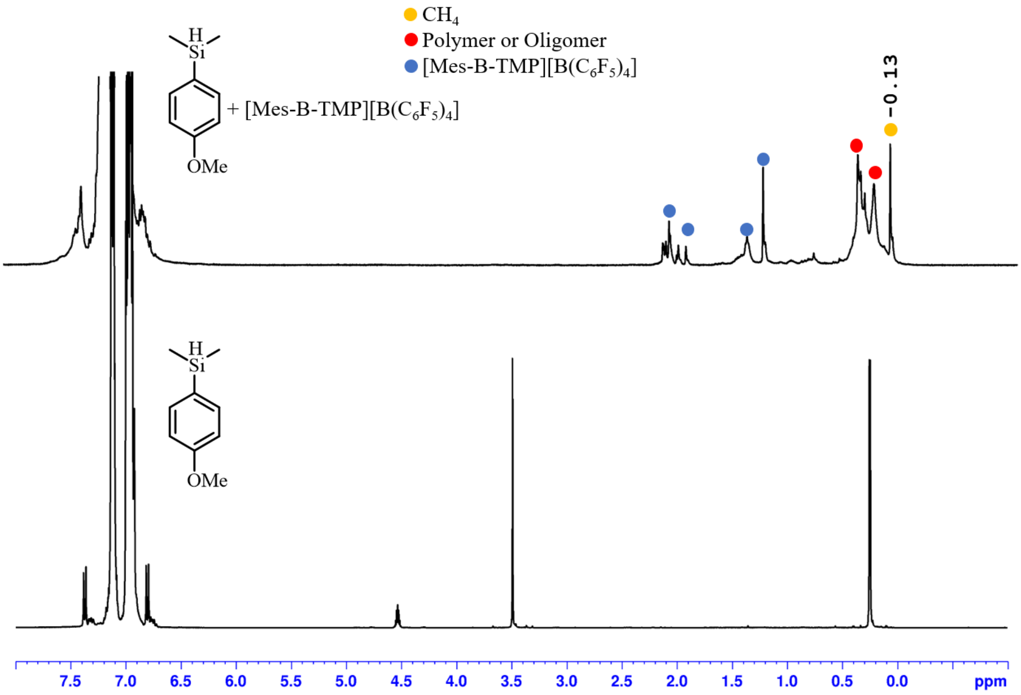

Figure S7. ^{1}**H NMR spectra of 2h with 1 mol% of [1][B(C_6F_5)$_4$] (Up) in comparison with 2h (Down) in PhCl solution.**

2.5 Cross-coupling of two silanes

We turned our attention to the cross-coupling of two different hydrosilanes (**Table S2**). Treating equimolar amounts of **2a** and **2d** with 1 mol% [**1**]$^+$ resulted in a mixture of products including **3d**, (4-Br-Ph)PhMe$_2$Si (**7d**), and **3a,** with yields of 1, 15, and 17, respectively (entry 1). Similar product distribution, but with a lower yield, was observed for **2b** (entry 3). The yield of the heteroleptic product can be improved by increasing the catalyst loading from 1 mol% to 3 mol% (entries 2 and 4). However, it also increases the yield of the undesired homo-coupling product. We hypothesize that at 1 mol% catalyst loading, the catalyst decomposes before the reaction reaches equilibrium, leading to low product yields. On the other hand, when using 3 mol% catalyst loading, the reaction reaches equilibrium, and the amount of **3d** increases dramatically compared to that in 1 mol% catalyst loading condition. To support this assumption, we repeated the reaction of **2a** and **2d** using a 3 mol% catalyst loading and monitored the reaction every 5 minutes by ^{1}H NMR (**Figure S8**). In the first 5 minutes, we have almost 40 % yield of (4-Br-Ph)PhMe$_2$Si and near 20 % yield of **3a** with only a trace amount of **3d**. The result is comparable to that in the 1 mol% catalyst loading condition (entry 1). Over time, the yield of **3d** increases to nearly 25 % after 1h. We think that the reason for the higher yield of **7d** and **3a** initially is due to **2d** being a weaker nucleophile than **2a**, making the formation of **3d** a less favorable process at the early stage of the reaction. As a result, the activated **2d** and **2a** are more likely to be attacked by the stronger nucleophile **2a**, resulting in the production of **7d** and **4a**. However, as the amount of **2a** decreases as the reaction proceeds, the process involving **3f** as the nucleophile becomes the dominate pathway leading to the formation of **3d**. Interestingly, a much higher yield of the heteroleptic product is accomplished when electron-rich thiophen-2-yl silane **2j** is examined (entry 5). We suggest that electron-rich silane **2j** can be a strong nucleophile to attack the activated phenyl silane. Although **2j** also can be activated by [**1**]$^+$, the activated thiophen-2-yl silane would be less favorable towards further reaction due to the electron richness of the silicon center compared to the activated phenyl silane. Therefore, electron-rich silane **2j** has higher selectivity in the cross-coupling reaction with **2a**.

Table S2. Cross-coupling of aryldimethylsilanes.

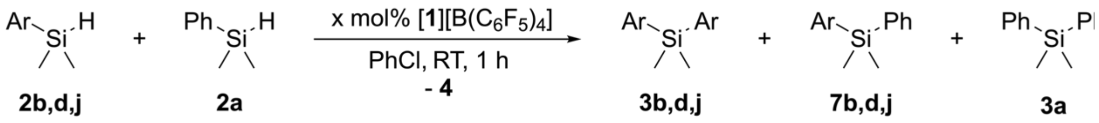

Entry	Substrate	x	Yield (**3** / **7** / **3a** [%])[b]
1	(**2d**) Ar = 4-Br-C_6H_4	1	1 / 15 / 17
2	(**2d**) Ar = 4-Br-C_6H_4	3	26 / 54 / 19
3	(**2b**) Ar = 2-tolenyl	1	4 / 12 / 13
4	(**2b**) Ar = 2-tolenyl	3	26 / 36 / 29
5	(**2j**) Ar = thiophen-2-yl	1	14 / 74 / 7

[a]Reactions were stirred open in glovebox.

[b]Yields were determined by ^{1}H NMR spectroscopy.

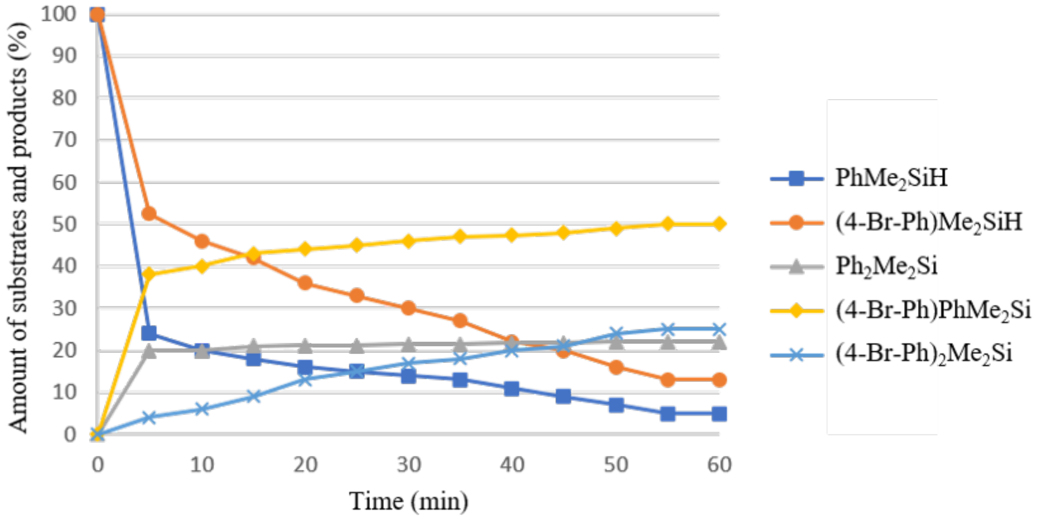

Figure S8. Dependence of product of 3f and 3a after adding 3 mol% of [2][B(C_6F_5)$_4$] on time (min)

Table S3. Cross-coupling of aryldimethylsilane and alkylsilanes

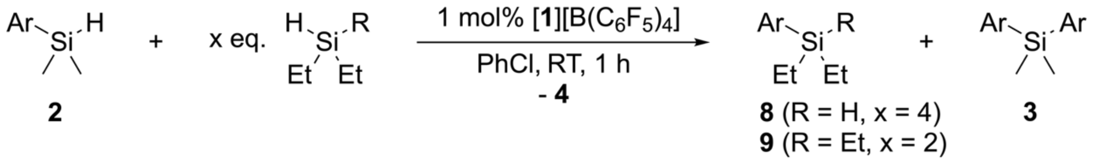

Entry	Substrate	R	x	Time (h)	Yield (**8** or **9** / **3** [%])
1	**2a** Ar = Ph	H	2	2	60 / 6
2	**2a** Ar = Ph	H	3	1	69 / 4
3	**2a** Ar = Ph	H	4	1	73 / 3
4	**2a** Ar = Ph	H	5	1	75 / 3
5	**2b** Ar = 2-tolyl	H	4	1	74 / <1
6	**2c** Ar = 4-tolyl	H	4	1	78 / 3
7	**2d** Ar = 4-Br-C_6H_4	H	4	12	74 / 2
8	**2e** Ar = biphenyl-4-yl	H	4	1	80 / <1
9	**2i** Ar = 2-naphthyl	H	4	1	77 / 1
10	**2j** Ar = 2-thiophenyl	H	4	1	74 / 4
11	**2a** Ar = Ph	Et	2	1	90 / 4
12	**2e** Ar = biphenyl-4-yl	Et	2	1	86 / 4
13	**2i** Ar = 2-naphthyl	Et	2	1	88 / 2
14	**2j** Ar = 2-thiophenyl	Et	2	1	97 / <1

[a]Reactions were constructed with 0.5 mL solvent in a J-Young tube.

[b]Yields were determined by ^{1}H NMR spectroscopy.

2.6 Comparison of boron catalyst

Table S4. Boron compound catalyzed redistribution of $PhMe_2SiH$

$$2\ PhMe_2SiH \xrightarrow{x\ \text{mol\% Catalyst}} Ph_2Me_2Si + Me_2SiH_2$$

Catalyst	x	Solvent	Temperature	Time (h)	Yield (%)
[a]$[Mes_2B]^+$	10	*o*-$C_6D_4Cl_2$	RT	1	0
[**1**]$^+$	10	*o*-$C_6D_4Cl_2$	RT	1	60
[**1**]$^+$	5	C_6H_5Cl	RT	1	63
[b]$B(C_6F_5)_3$	5	C_6D_5Cl	100°C	24	trace

[a]Catalyst was fully decomposed. [b]Experiment was reported by literature.[2]

3. Synthetic Details

3.1 Synthesis of catalyst [1][$B(C_6F_5)_4$]

A solution of MesTMPBCl (10 mg, 0.033 mmol) in $CDCl_3$ in J-Young tube was added Na[$B(C_6F_5)_4$] (23.2 mg, 0.033 mmol) to get a brown solution with white solid in the bottom. A solution was filtered to remove white solid, and the solvent was removed under vacuum affording brown oil (27.7 mg, 88% yield). ^1H NMR (400 MHz, $CDCl_3$): δ = 7.13 (s, 2H), 2.63 (s, 6H), 2.44 (s, 3H), 1.95-1.86 (m, 2H), 1.77-1.71 (m, 4H), 1.62 (s, 12H) ppm. ^{11}B NMR (128 MHz, $CDCl_3$): δ = 54.5 (br) ppm. $^{13}C\{^1H\}$ NMR (100 MHz, $CDCl_3$): δ = 156.2, 153.4, 149.1, 147.2, 139.1, 137.1, 135.2, 130.1, 60.4, 37.5, 30.9, 23.1, 22.8, 16.4 ppm. $^{19}F\{^1H\}$ NMR (376 MHz, $CDCl_3$): δ = -131.8, -162.3, -166.1 ppm.

3.2 Synthesis and characterization of substrates

General procedure A:

Corresponding bromo-arene compounds (30 mmol, 1 eq.) dissolve in 50 mL THF and cool to -78 °C. Then *n*-BuLi (2.5 M solution in hexane, 14.4 mL, 36 mmol, 1.2 eq.) was added dropwise and the reaction mixture was stirred for 3 hours at -78 °C. Then chlorodimethylsilane (4.331 mL, 39 mmol, 1.3 eq.) was added dropwise then allowed to warm to room temperature and was stirred overnight. The crude reaction mixture was quenched by $NH_4Cl_{(aq)}$ and extracted with Et_2O and brine. The organic layer was dried over Na_2SO_4 and concentrated under reduced pressure. Purification was performed by column chromatography (Hexane/Et_2O = 100/1) or distillation to give the corresponding silanes. Silane **2b**, **2d**, **2f-h** were synthesized by procedure A.

General procedure B:

In a 100 mL three-neck round-bottom flask equipped with a reflux condenser contain Mg (874.8 mg, 36 mmol, 1.2 eq.) with 30 mL THF. Corresponding bromo-substituted compounds (30 mmol, 1 eq.) dissolving in 20 mL THF was added dropwise and the reaction mixture was reflux and stirred for 1 hour affording a Grignard reagent. Another flask that contains chlorodimethylsilane (4.331 mL, 39 mmol, 1.3 equiv) and THF 30 mL was cooled to -78 °C. Then the Grignard reagent was added dropwise to the flask, and slowly warm to room temperature and was stirred overnight. The crude reaction mixture was quenched by $NH_4Cl_{(aq)}$ and extracted with Et_2O and brine. The organic layer was dried over Na_2SO_4 and concentrated under reduced pressure. Purification was performed by column chromatography (Hexane/Et_2O = 100/1) or distillation to give corresponding silanes. Silane **2c, 2e, 2i, 2j** were synthesized by procedure B.

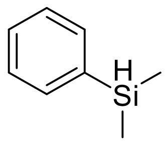

dimethyl(phenyl)silane (2a)[3]

Colorless oil. ^{1}H NMR (400 MHz, $CDCl_3$): δ = 7.76-7.82 (m, 2H) ,7.56-7.61 (m, 3H), 4.72 (sept, J = 3.7 Hz, 1H), 0.59 ppm (d, J = 3.7 Hz, 6H); ^{13}C{^{1}H} NMR (126 MHz, $CDCl_3$): δ = 137.5, 134.1, 129.3, 127.9, -3.7 ppm; ^{29}Si{^{1}H} NMR (99 MHz, $CDCl_3$): δ = -17.1 ppm.

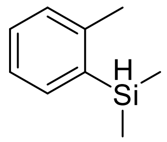

dimethyl(*o*-tolyl)silane (2b)[4]

Colorless oil. ^{1}H NMR (500 MHz, $CDCl_3$): δ = 7.52 (d, J = 7.5 Hz, 1H) , 7.33 (td, J = 7.5 Hz, 1H) ,7.22 (t, J = 7.5 Hz, 2H) , 4.59 (sept, 1H) , 2.51 (s, 3H), 0.40 ppm (d, J = 3.9 Hz, 6H); ^{13}C{^{1}H} NMR (126 MHz, $CDCl_3$): δ = 143.8, 136.3, 134.7, 129.7, 129.6, 125.2, 22,5, -3.4 ppm; ^{29}Si{^{1}H} NMR (99 MHz, $CDCl_3$): δ = -20.2 ppm.

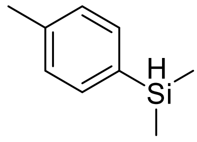

dimethyl(*p*-tolyl)silane (2c)[5]

Colorless oil. ^{1}H NMR (500 MHz, $CDCl_3$): δ = 7.48 (d, J = 8.1 Hz, 2H), 7.22 (d, J = 8.1 Hz, 2H), 4.57 (sept, 1H), 2.39 (s, 3H), 0.37 ppm (d, J = 4.0 Hz, 6H); ^{13}C{^{1}H} NMR (126 MHz, $CDCl_3$): δ = 139.2, 134.2, 133.9, 128.7, 129.9, 21.6, -3.5 ppm; ^{29}Si{^{1}H} NMR (99 MHz, $CDCl_3$): δ = -17.5 ppm.

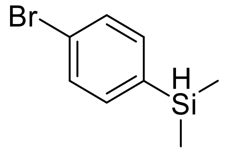

(4-bromophenyl)dimethylsilane (2d)[5]

Colorless oil. ^{1}H NMR (500 MHz, $CDCl_3$): δ = 7.59 (d, J = 8.2 Hz, 2H), 7.49 (d, J = 8.2 Hz, 2H), 4.53 (sept, J = 3.7 Hz, 1H), 0.44 ppm (d, J = 3.8 Hz, 6H); ^{13}C{^{1}H} NMR (126 MHz, $CDCl_3$): δ = 136.1, 135.6, 131.0, 124.0, -3.8 ppm; ^{29}Si{^{1}H} NMR (99 MHz, $CDCl_3$): δ = -16.7 ppm.

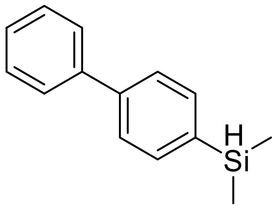

[1,1'-biphenyl]-4-yldimethylsilane (2e)[4]

Colorless oil. ^{1}H NMR (500 MHz, $CDCl_3$): δ = 7.72-7.65 (m, 6H), 7.52 (t, J = 7.5 Hz, 2H),7.42 (tt, J = 7.4 Hz, 1H) 4.57 (sept, J = 3.7 Hz, 1H), 0.46 ppm (d, J = 3.9 Hz, 6H); ^{13}C{^{1}H} NMR (126 MHz, $CDCl_3$): δ = 142.2, 141.2, 136.3, 134.7, 128.9, 127.6, 127.3, 126.8, -3.6 ppm; ^{29}Si{^{1}H} NMR (99 MHz, $CDCl_3$): δ = -17.2 ppm.

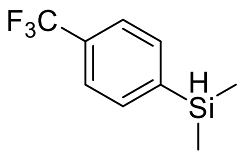

dimethyl(4-(trifluoromethyl)phenyl)silane (2f)[5]

Colorless oil. ^{1}H NMR (500 MHz, $CDCl_3$): δ = 7.68 (d, J = 8.3 Hz, 2H), 7.62 (d, J = 8.3 Hz, 2H), 4.48 (sept, J = 3.7 Hz, 1H), 0.39 ppm (d, J = 3.8 Hz, 6H); ^{13}C{^{1}H} NMR (126 MHz, $CDCl_3$): δ = 142.6, 134.5, 131.4 (q, J = 32.0 Hz), 124.6 (q, J = 3.7 Hz), 124.5 (q, J = 3.8 Hz), -3.9 ppm; ^{29}Si{^{1}H} NMR (99 MHz, $CDCl_3$): δ = -16.4 ppm.

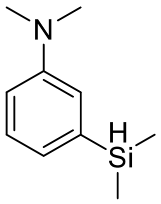

3-(dimethylsilyl)-*N*,*N*-dimethylaniline (2g)[2]

Colorless oil. ^{1}H NMR (500 MHz, $CDCl_3$): δ = 7.31 (dd, J = 7.97 Hz, 1H), 7.00-6.95 (m, 2H), 6.82 (dddd, J = 0.9 Hz, 1H), 4.47 (sept, J = 3.67 Hz, 1H), 3.01 (s, 6H), 0.39 ppm (d, J = 3.8 Hz, 6H), ^{13}C{^{1}H} NMR (126 MHz, $CDCl_3$): δ = 150.2, 138.1, 128.8, 122.3, 118.0, 113.8, 40.7, -3.5 ppm; ^{29}Si{^{1}H} NMR (99 MHz, $CDCl_3$): δ = -16.5 ppm.

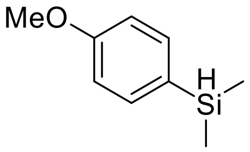

(*p*-methoxyphenyl)dimethylsilane (2h)[5]

Colorless oil. ^{1}H NMR (500 MHz, $CDCl_3$): δ = 7.50 (d, J = 8.57 Hz, 2H), 6.95 (d, J = 8.7 Hz, 2H), 4.49 (sept, J = 3.9 Hz, 1H), 3.84 (s, 3H), 0.36 ppm (d, J = 3.9 Hz, 6H); ^{13}C{^{1}H} NMR (126 MHz, $CDCl_3$): δ = 160.7, 135.6, 128.3, 113.8, 55.2, -3.4 ppm; ^{29}Si{^{1}H} NMR (99 MHz, $CDCl_3$): δ = -17.8 ppm.

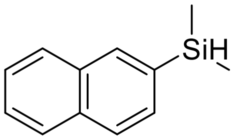

dimethyl(naphthalen-2-yl)silane (2i)[4]

Colorless oil. ^{1}H NMR (500 MHz, $CDCl_3$): δ = 8.11 (s, 1H), 7.93-7.85 (m, 3H), 7.67 (dd, J = 8.1 Hz, 1H), 7.57-7.51 (m, 2H), 4.63 (sept, J = 3.6 Hz, 1H), 0.49 ppm (d, J = 3.7 Hz, 6H); ^{13}C{^{1}H} NMR (126 MHz, $CDCl_3$): δ = 135.0, 134.8, 133.9, 133.1, 130.3, 128.1, 127.9, 127.2, 126.5, 126.1, -3.5 ppm; ^{29}Si{^{1}H} NMR (99 MHz, $CDCl_3$): δ = -16.6 ppm.

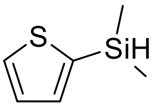

dimethyl(thiophen-2-yl)silane (2j)[4]

Colorless oil. ^{1}H NMR (500 MHz, $CDCl_3$): δ = 7.69 (dd, J = 4.6 Hz, 1H), 7.40 (d, J = 3.3 Hz, 1H), 7.27 (dd, J = 3.4 Hz, 1H), 4.67 (sept, J = 3.5 Hz, 1H), 0.48 ppm (d, J = 3.6 Hz, 6H); ^{13}C{^{1}H} NMR (126 MHz, $CDCl_3$): δ = 136.3, 135.2, 131.2, 128.4, -2.6 ppm; ^{29}Si{^{1}H} NMR (99 MHz, $CDCl_3$): δ = -23.2 ppm.

3.3 Synthesis and characterization of homo-metathesis product

General procedure of homo-metathesis:

In a nitrogen-filled glovebox, hydrosilanes (0.45 mmol) and internal standard mesitylene (10 uL) and solvent PhCl were mixed in a vial. and the total volume of the solution was 150 uL. Then took 50 uL of the mixture to measure the initial ratio of hydrosilanes and internal standard mesitylene by ^{1}H NMR spectrum. The remaining mixture, which contains hydrosilanes (0.30 mmol) and mesitylene (6.66 uL) was added a PhCl solution of catalyst (0.003 mmol) (100 uL). Then the mixture was stirred open to a nitrogen atmosphere at room temperature for 1 hour. After that the resulting mixture was diluted with PhCl, and tested the ^{1}H NMR spectrum. Purification was performed by column chromatography (Hexane/Et_2O = 100/1) or distillation to give corresponding products.

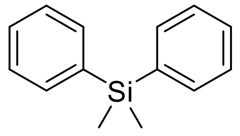

dimethyldiphenylsilane (3a)[6]

colorless oil, ^{1}H NMR (500 MHz, $CDCl_3$): δ = 7.55-7.50 (m, 4H), 7.40-7.32 (m, 6H), 0.56 ppm (s, 6H); ^{13}C{^{1}H} NMR (126 MHz, $CDCl_3$): δ = 138.4, 134.3, 129.2, 127.9, -2.3 ppm; ^{29}Si{^{1}H} NMR (99 MHz, $CDCl_3$): δ = -8.1 ppm.

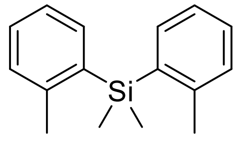

dimethyldi-*o*-tolylsilane (3b)[7]

colorless oil, ^{1}H NMR (500 MHz, $CDCl_3$): δ = 7.55 (dd, J = 7.18Hz, 2H), 7.31 (td, J = 7.6 Hz, 2H), 7.20 (t, J = 7.6 Hz, 2H), 7.15(d, J = 7.6 Hz, 2H), 2.23 (s, 6H), 0.65 ppm (s, 6H); ^{13}C{^{1}H} NMR (126 MHz, $CDCl_3$): δ = 143.9, 137.2, 134.9, 129.9, 129.5, 125.2, 23.1, -0.76 ppm; ^{29}Si{^{1}H} NMR (99 MHz, $CDCl_3$): δ = -8.8 ppm.

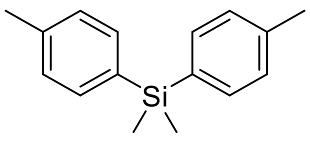

dimethyldi-*p*-tolylsilane (3c)[8]

colorless oil, ^{1}H NMR (500 MHz, $CDCl_3$): δ = 7.42 (d, J = 8.5 Hz, 2H), 7.18 (d, J = 7.8 Hz, 2H), 2.36 (s, 6H), 0.53 ppm (s, 6H); ^{13}C{^{1}H} NMR (126 MHz, $CDCl_3$): δ = 139.9, 134.9, 134.4, 128.8, 21.6, -2.07 ppm; ^{29}Si{^{1}H} NMR (99 MHz, $CDCl_3$): δ = -8.5 ppm.

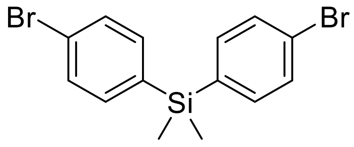

bis(4-bromophenyl)dimethylsilane (3d)[9]

White solid. ^{1}H NMR (500 MHz, $CDCl_3$): δ = 7.50 (d, J = 7.6 Hz, 4H), 7.35 (d, J = 7.6 Hz, 4H), 0.54 (s, 6H) ppm; ^{13}C{^{1}H} NMR (126 MHz, $CDCl_3$): δ = 136.6, 135.8, 131.2, 124.4, -2.4 ppm; ^{29}Si{^{1}H} NMR (99 MHz, $CDCl_3$): δ = -7.0 ppm.

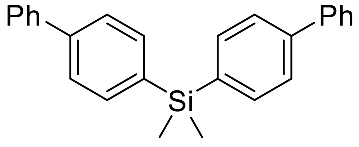

di([1,1'-biphenyl]-4-yl)dimethylsilane (3e)[10]

White solid. ^{1}H NMR (500 MHz, $CDCl_3$): δ = 7.70-7.60 (m, 12H), 7.46 (t, J = 8.4 Hz, 4H),7.37 (tt, J = 7.9 Hz, 2H), 0.65 ppm (s, 6H); ^{13}C{^{1}H} NMR (126 MHz, $CDCl_3$): δ = 142.0, 141.2, 137.0, 134.8, 128.9, 127.5, 127.3, 126.7, -2.15 ppm; ^{29}Si{^{1}H} NMR (99 MHz, $CDCl_3$): δ = -8.00 ppm.

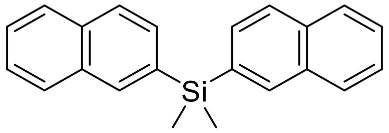

dimethyldi(naphthalen-2-yl)silane (3i)

Colorless oil. ^{1}H NMR (500 MHz, $CDCl_3$): δ = 8.06 (s, 2H), 7.81 (d, J = 9.1 Hz, 6H),

7.63 (d, J = 8.8 Hz, 2H), 7.52-7.44 (m, 4H), 0.72 ppm (s, 6H); $^{13}C\{^{1}H\}$ NMR (126 MHz, $CDCl_3$): δ = 135.8, 135.1, 133.9, 133.1, 130.6, 128.2, 127.9, 127.2, 126.6, 126.1, -2.1 ppm; $^{29}Si\{^{1}H\}$ NMR (99 MHz, $CDCl_3$): δ = -7.3 ppm.

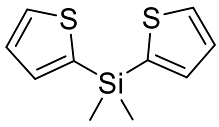

dimethyldi(thiophen-2-yl)silane (3j)[11]

Colorless oil. ^{1}H NMR (400 MHz, $CDCl_3$): δ = 7.62 (dd, J = 4.3 Hz, 2H), 7.32 (d, J = 3.7 Hz, 2H), 7.19 (dd, J = 4.5 Hz, 2H), 0.65 ppm (s, 6H). $^{13}C\{^{1}H\}$ NMR (126 MHz, $CDCl_3$): δ = 137.3, 135.4, 131.2, 128.1, -0.1 ppm; $^{29}Si\{^{1}H\}$ NMR (99 MHz, $CDCl_3$): δ = -15.3 ppm.

3.4 Synthesis and characterization of cross-coupling product

General procedure of cross-coupling of $ArMe_2SiH$ and $PhMe_2SiH$:

In a nitrogen-filled glovebox, $ArMe_2SiH$ (0.45 mmol) and $PhMe_2SiH$ (0.45 mmol) and internal standard mesitylene (10 uL) and solvent PhCl were mixed in a vial. and the total volume of the solution was 150 uL. Then took 50 uL of the mixture to measure the initial ratio of hydrosilanes and internal standard mesitylene by ^{1}H NMR spectrum. The remaining mixture, which contains hydrosilanes (0.30 mmol) and mesitylene (6.66 uL) was added a PhCl solution of catalyst (0.003 mmol) (100 uL). Then the mixture was stirred open to a nitrogen atmosphere at room temperature for 1 hour. After that the resulting mixture was diluted with PhCl and monitor by ^{1}H NMR spectrum.

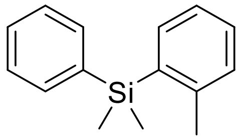

dimethyl(phenyl)(*o*-tolyl)silane (7b)[12]

Colorless oil. ^{1}H NMR (400 MHz, $CDCl_3$): δ = 7.56-7.50 (3H), 7.45-7.36 (3H), 7.36-7.30 (1H), 7.26-7.14 (2H), 2.29 (3H), 0.61 ppm (s, 6H); $^{13}C\{^{1}H\}$ NMR (126 MHz, $CDCl_3$): δ = 144.2, 139.1, 136.3, 135.5, 134.1, 130.0, 129.7, 129.1, 125.1, 23.3, -1.3 ppm; $^{29}Si\{^{1}H\}$ NMR (99 MHz, $CDCl_3$): δ = -8.1 ppm.

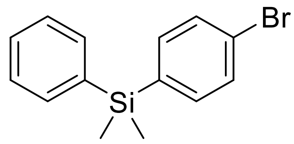

(4-bromophenyl)dimethyl(phenyl)silane (7d)[13]

Colorless oil. ^1H NMR (400 MHz, $CDCl_3$): δ = 7.55-7.47 (4H), 7.44-7.32 (5H), 0.55 ppm (s, 6H); $^{13}C\{^1H\}$ NMR (126 MHz, $CDCl_3$): δ = 137.7, 137.3, 135.9, 134.2, 131.1, 129.5, 128.1, 124.1, -2.4 ppm; $^{29}Si\{^1H\}$ NMR (99 MHz, $CDCl_3$): δ = -7.6 ppm.

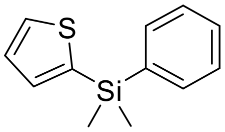

dimethyl(phenyl)(thiophen-2-yl)silane (7j)[14]

Colorless oil. ^1H NMR (400 MHz, $CDCl_3$): δ = 7.70-7.63 (1H), 7.62-7.58 (2H), 7.44-7.34 (3H), 7.34-7.30 (1H), 7.26-7.20 (1H), 0.61 ppm (s, 6H); $^{13}C\{^1H\}$ NMR (126 MHz, $CDCl_3$): δ = 138.0, 137.9, 135.5, 134.1, 131.3, 129.5, 128.3, 128.0, -1.1 ppm; $^{29}Si\{^1H\}$ NMR (99 MHz, $CDCl_3$): δ = -11.6 ppm.

General Procedure of Cross-Coupling of $ArMe_2SiH$ with Et_2SiH_2/Et_3SiH:

Hydrosilanes (0.30 mmol) and Et_2SiH_2/Et_3SiH (0.60-1.5 mmol) and internal standard mesitylene (10 uL) and solvent PhCl were mixed in a J-Young NMR tube. Then measured the initial ratio of hydrosilanes and internal standard mesitylene by ^1H NMR spectrum. Then the mixture was added a PhCl solution of catalyst (0.003 mmol) (100 uL). tested the ^1H NMR spectrum. Purification was performed by column chromatography (Hexane/Et_2O = 100/1) or distillation to give corresponding products.

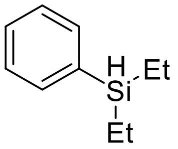

Diethyl(phenyl)silane (8a)[15]

Colorless oil. ^1H NMR (400 MHz, $CDCl_3$): δ = 7.54-7.50 (m, 2H), 7.37-7.31 (m, 3H), 4.19 (quint, 1H), 1.00 (t, J = 9.3Hz, 6H), 0.86 ppm (m, 4H); $^{13}C\{^1H\}$ NMR (126 MHz,

CDCl3): δ = 135.7, 134.8, 129.3, 127.9, 8.2, 3.6 ppm; $^{29}Si\{^{1}H\}$ NMR (99 MHz, CDCl3): δ = -5.1 ppm.

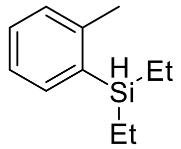

diethyl(*o*-tolyl)silane (8b)

Colorless oil. ^{1}H NMR (500 MHz, $CDCl_3$): δ = 7.45 (d, J = 8.85 Hz, 1H), 7.28 (td, J = 7.25 Hz, 1H) ,7.17 (t, J = 7.73 Hz, 2H) , 4.32 (quint, J = 3.57 Hz 1H) , 2.45 (s, 3H), 1.0 (t, J = 8.3 Hz, 6H), 0.91-0.83 ppm (m, 4H); $^{13}C\{^{1}H\}$ NMR (126 MHz, $CDCl_3$): δ = 144.0, 135.5, 134.5, 129.6, 129.5, 125.0, 22,7, 8.5, 3.7 ppm; $^{29}Si\{^{1}H\}$ NMR (99 MHz, $CDCl_3$): δ = -7.5 ppm.

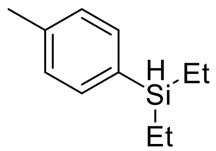

diethyl(*p*-tolyl)-silane (8c)

Colorless oil. ^{1}H NMR (500 MHz, $CDCl_3$): δ = 7.44 (d, J = 7.29 Hz, 2H), 7.22 (d, J = 7.29 Hz, 2H), 4.20 (quint, J = 3.19 Hz 1H) , 2.36 (s, 3H), 1.0 (t, J = 7.59Hz, 6H), 0.91-0.83 ppm (m, 4H); $^{13}C\{^{1}H\}$ NMR (126 MHz, $CDCl_3$): δ = 139.1, 134.8, 131.9, 128.8, 21,6, 8.3, 3.7 ppm; $^{29}Si\{^{1}H\}$ NMR (99 MHz, $CDCl_3$): δ = -5.4 ppm.

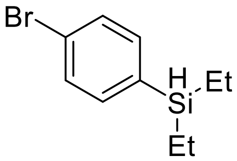

(4-bromophenyl)diethylsilane (8d)

Colorless oil. ^{1}H NMR (500 MHz, $CDCl_3$): δ = 7.49 (d, J = 7.82 Hz, 2H), 7.39 (d, J = 6.80 Hz, 2H), 4.17 (quint, J = 3.40 Hz 1H), 1.0 (t, J = 7.8Hz, 6H), 0.86-0.79 ppm (m, 4H); $^{13}C\{^{1}H\}$ NMR (126 MHz, $CDCl_3$): δ = 136.2, 134.4, 130.9, 124.0, 8.0, 3.3 ppm; $^{29}Si\{^{1}H\}$ NMR (99 MHz, $CDCl_3$): δ = -4.8 ppm.

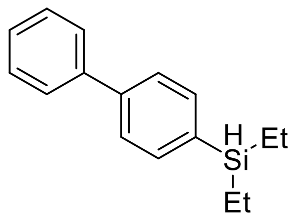

[1,1'-biphenyl]-4-yldiethylsilane (8e)[16]

Colorless oil. ^{1}H NMR (500 MHz, $CDCl_3$): δ = 7.69-7.61 (m, 6H), 7.48 (t, J = 8.1 Hz, 2H),7.39 (tt, J = 7.42 Hz, 1H) 4.31 (quint, J = 3.84 Hz, 1H), 1.1 (t, J = 7.61 Hz, 6H), 0.97-0.88 ppm (m, 4H); ^{13}C{^{1}H} NMR (126 MHz, $CDCl_3$): δ = 142.0, 141.2, 135.3, 134.5, 128.9, 127.5, 127.3, 126.7, 8.3, 3.6 ppm; ^{29}Si{^{1}H} NMR (99 MHz, $CDCl_3$): δ = -5.3 ppm.

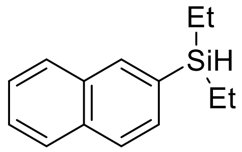

diethyl(naphthalen-2-yl)silane (8i)

Colorless oil. ^{1}H NMR (500 MHz, $CDCl_3$): δ = 8.08 (s, 1H), 7.90-7.80 (m, 3H), 7.62 (dd, J = 7.56 Hz, 1H), 7.54-7.48 (m, 2H), 4.37 (quint, J = 3.45 Hz, 1H), 1.08 (t, J = 6.70 Hz, 6H), 0.99-0.92 ppm (m, 4H); ^{13}C{^{1}H} NMR (126 MHz, $CDCl_3$): δ = 135.5, 133.8, 133.1, 133.0, 130.8, 128.0, 127.8, 127.0, 126.3, 125.9, 8.2, 3.6. ppm; ^{29}Si{^{1}H} NMR (99 MHz, $CDCl_3$): δ = -4.5 ppm.

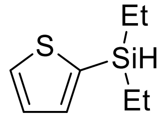

diethyl(thiophen-2-yl)silane (8j)

Colorless oil. ^{1}H NMR (500 MHz, $CDCl_3$): δ = 7.63 (dd, J = 4.64 Hz, 1H), 7.33 (dd, J = 3.39 Hz, 1H), 7.22 (dd, J = 4.49 Hz, 1H), 4.36 (quint, J = 3.3 Hz, 1H), 1.05 (t, J = 8.3 Hz, 6H), 0.90-0.83 ppm (m, 4H); ^{13}C{^{1}H} NMR (126 MHz, $CDCl_3$): δ = 135.6, 133.8, 131.0, 128.1, 7.9, 4.2 ppm; ^{29}Si{^{1}H} NMR (99 MHz, $CDCl_3$): δ = -12.3 ppm.

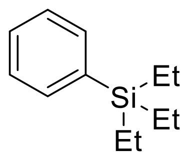

triethyl(phenyl)silane (9a)[17]

Colorless oil. ^{1}H NMR (400 MHz, $CDCl_3$): δ = 7.52-7.47 (m, 2H), 7.37-7.33 (m, 3H), 0.97 (t, J = 7.63Hz, 9H), 0.80 ppm (q, J = 7.30 Hz, 6H); ^{13}C{^{1}H} NMR (126 MHz, $CDCl_3$): δ = 137.6, 134.3, 128.8, 127.8, 7.5, 3.5 ppm; ^{29}Si{^{1}H} NMR (99 MHz, $CDCl_3$): δ = 1.8 ppm.

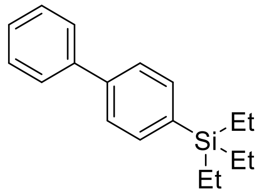

[1,1'-biphenyl]-4-yltriethylsilane (9e)[18]

Colorless oil. ^{1}H NMR (500 MHz, $CDCl_3$): δ = 7.65-7.60 (m, 6H), 7.47 (t, J = 6.5 Hz, 2H),7.39 (tt, J = 7.34 Hz, 1H), 1.03 (t, J = 7.61 Hz, 9H), 0.86 ppm (q, J = 7.46 Hz, 6H); ^{13}C{^{1}H} NMR (126 MHz, $CDCl_3$): δ = 141.5, 141.3, 136.4, 135.3, 128.9, 127.4, 127.3, 126.5, 7.6, 3.6 ppm; ^{29}Si{^{1}H} NMR (99 MHz, $CDCl_3$): δ = 1.9 ppm.

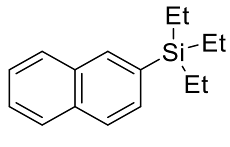

triethyl(naphthalen-2-yl)silane (9i)[18]

Colorless oil. ^{1}H NMR (500 MHz, $CDCl_3$): δ = 8.01 (s, 1H), 7.88-7.82 (m, 3H), 7.60 (dd, J = 8.30 Hz, 1H), 7.53-7.48 (m, 2H), 1.03 (t, J = 7.90 Hz, 9H), 0.91 ppm (q, J = 8.48 Hz, 6H); ^{13}C{^{1}H} NMR (126 MHz, $CDCl_3$): δ = 135.1, 134.9, 133.8, 133.1, 130.7, 128.1, 127.8, 126.9, 126.3, 125.9, 7.6, 3.5 ppm; ^{29}Si{^{1}H} NMR (99 MHz, $CDCl_3$): δ = 2.3 ppm.

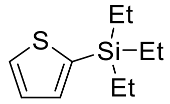

triethyl(thiophen-2-yl)silane (9j)[19]

Colorless oil. ^{1}H NMR (500 MHz, $CDCl_3$): δ = 7.60 (dd, J = 5.02 Hz, 1H), 7.26 (dd, J = 3.37 Hz, 1H), 7.20 (dd, J = 4.49 Hz, 1H), 1.00 (t, J = 8.0 Hz, 9H), 0.90-0.83 ppm (q, J = 7.6 Hz, 6H); $^{13}C\{^1H\}$ NMR (126 MHz, $CDCl_3$): δ = 136.5, 134.6, 130.4, 128.0, 7.3, 4.5 ppm; $^{29}Si\{^1H\}$ NMR (99 MHz, $CDCl_3$): δ = 0.0 ppm.

4. Computational Details

All quantum chemical calculations were performed with ORCA 6.0.1[20, 21] in the following procedure: The geometries were optimized in gas phase using the r^2SCAN-3c composite method with tight self-consistent field convergence criteria and tight geometric convergence criteria.[22-24] Harmonic frequency calculations at the same level of theory were carried out to verify that the optimized structures correspond to either local minimums (with zero imaginary vibrational frequency) or transition states (with one imaginary vibrational frequency). Quasi rigid-rotor harmonic oscillator model (quasi-RRHO) with Grimme's entropy interpolation method was applied for low frequencies (< 100 cm^{-1}) to calculate the zero-point energies (ZPE) and thermo corrections ($G_{corr} = G - EE$) at 1.00 atm and 298.15 K using ideal gas relation without frequency scaling factor.[25] Symmetry factors are manually assigned with the ignorance of hydrogen atoms. Higher accuracy electronic energies (EE) were performed on the optimized geometry under DLPNO-CCSD(T)/cc-pVTZ,[26, 27] and VeryTightSCF and TightPNO criteria was used to ensure accurate results. Solvation free energies were obtained from the difference in electronic energy of the optimized structure with and without implicit solvation model SMD(*o*-dichlorobenzene)[28] under M06-2X[29]/cc-pVTZ and an additional phase transition energy (1.00 atm gaseous phase to 1.0000 M solution phase) of 0.003018804 Hartree was added. All calculations were performed with DefGrid3 integration grid. The total free energies for each compound were calculated as the summation of single point, G_{corr}, and solvation energies, as shown below.

$$G_{\mathrm{tot}} = G_{\mathrm{gas}} + \Delta G_{\mathrm{sol}}$$

$$\begin{cases} G_{\mathrm{gas}} = \mathrm{EE}^{\text{DLPNO-CCSD(T)/cc-pVTZ}} + G_{\mathrm{corr}}^{\mathrm{r^2SCAN\text{-}3c}} \\ \Delta G_{\mathrm{sol}} = \mathrm{EE}_{\mathrm{SMD(ODCB)}}^{\text{M06-2X/cc-pVTZ}} - \mathrm{EE}^{\text{M06-2X/cc-pVTZ}} + \Delta G_{\mathrm{phase}} \end{cases}$$

$$\Delta G_{\mathrm{phase}} = k_{\mathrm{B}} T \ln\left(\frac{RT/p}{1\ \mathrm{L}}\right) = 0.003018804\ \text{Hartree, for } \begin{cases} p = 1.00\ \mathrm{atm} \\ T = 298.15\ \mathrm{K} \end{cases}$$

$$1\ \text{Hartree} = 627.5094741\ \mathrm{kcal/mol}$$

Table S5. Calculated energys of optimized structures.

	G_{coor}	SP	EE_{sol}	EE_{vac}
[**1**]$^+$	0.387841	-781.687672	-782.808072	-782.731371
$PhMe_2SiH$	0.137238	-600.760612	-601.558794	-601.548320
Ph_2SiMe_2	0.213985	-831.485184	-832.615112	-832.596330
Me_2SiH_2	0.062169	-370.039701	-370.504070	-370.502262
[**TS-1**]$^+$	0.551866	-1382.460385	-1384.363252	-1384.286056
[**Int-1**]$^+$	0.552872	-1382.465111	-1384.367397	-1384.289354
[**TS-2**]$^+$	0.717925	-1983.240735	-1985.929378	-1985.849537
**1**-H	0.398806	-782.510206	-783.570818	-783.552987
[**Int-2**]$^+$	0.293490	-1200.711010	-1202.358314	-1202.281636
[**TS-3**]$^+$	0.718456	-1983.236233	-1985.924422	-1985.844540
[**Int-3**]$^+$	0.475085	-1151.729206	-1153.306922	-1153.232280
[**TS-4**]$^+$	0.473837	-1151.727040	-1153.303424	-1153.231104
[$^{\mathbf{CBN}}\mathbf{TS_{BC}}$]$^+$	0.555473	-1382.454962	-1384.355438	-1384.279213
[$^{\mathbf{CBN}}\mathbf{Int_{BC}}$]$^+$	0.556801	-1382.477811	-1384.375944	-1384.299720
[$^{\mathbf{CBN}}\mathbf{TS_{BN}}$]$^+$	0.557581	-1382.417628	-1384.321245	-1384.242032
[$^{\mathbf{CBN}}\mathbf{Int_{BN}}$]$^+$	0.561121	-1382.440336	-1384.342573	-1384.262896
[$\mathbf{Mes_2B}$]$^+$	0.299838	-722.728899	-723.797792	-723.721287
[$^{\mathbf{CBC}}\mathbf{TS_{SiH}}$]$^+$	0.463398	-1323.510783	-1325.364000	-1325.285230
[$^{\mathbf{CBC}}\mathbf{Int_{SiH}}$]$^+$	0.465700	-1323.523268	-1325.376374	-1325.296402
[$^{\mathbf{CBC}}\mathbf{TS_{BC}}$]$^+$	0.467061	-1323.508300	-1325.361323	-1325.282768
[$^{\mathbf{CBC}}\mathbf{Int_{BC}}$]$^+$	0.469266	-1323.535834	-1325.387162	-1325.308156

Table S6. Calculated solvation and total energys of optimized structures.

	ΔG_{solv}	symmetry	G_{tot}
[**1**]$^+$	-0.073682	1	-781.3735
$PhMe_2SiH$	-0.007455	1	-600.6308
Ph_2SiMe_2	-0.015764	2	-831.2870
Me_2SiH_2	0.001211	2	-369.9763
[**TS-1**]$^+$	-0.074177	1	-1381.9827
[**Int-1**]$^+$	-0.075024	1	-1381.9873
[**TS-2**]$^+$	-0.076822	1	-1982.5996
**1**-H	-0.014812	1	-782.1262
[**Int-2**]$^+$	-0.073659	1	-1200.4912
[**TS-3**]$^+$	-0.076863	1	-1982.5946
[**Int-3**]$^+$	-0.071623	1	-1151.3257
[**TS-4**]$^+$	-0.069301	1	-1151.3225
[$^{\mathbf{CBN}}\mathbf{TS_{BC}}$]$^+$	-0.073207	1	-1381.9727
[$^{\mathbf{CBN}}\mathbf{Int_{BC}}$]$^+$	-0.073205	1	-1381.9942
[$^{\mathbf{CBN}}\mathbf{TS_{BN}}$]$^+$	-0.076194	1	-1381.9362
[$^{\mathbf{CBN}}\mathbf{Int_{BN}}$]$^+$	-0.076659	1	-1381.9559
[$\mathbf{Mes_2B}$]$^+$	-0.073486	4	-722.5025
[$^{\mathbf{CBC}}\mathbf{TS_{SiH}}$]$^+$	-0.075752	1	-1323.1231
[$^{\mathbf{CBC}}\mathbf{Int_{SiH}}$]$^+$	-0.076953	1	-1323.1345
[$^{\mathbf{CBC}}\mathbf{TS_{BC}}$]$^+$	-0.075536	1	-1323.1168
[$^{\mathbf{CBC}}\mathbf{Int_{BC}}$]$^+$	-0.075987	1	-1323.1426

5. NMR Spectra

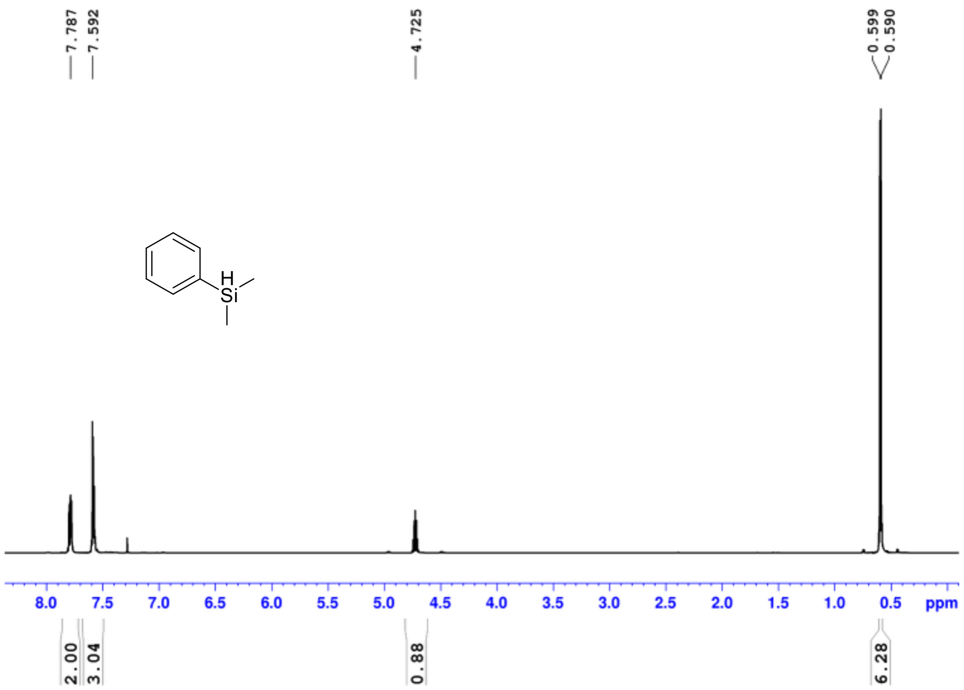

Figure S9. ^{1}H **NMR spectrum of** $PhMe_2SiH$

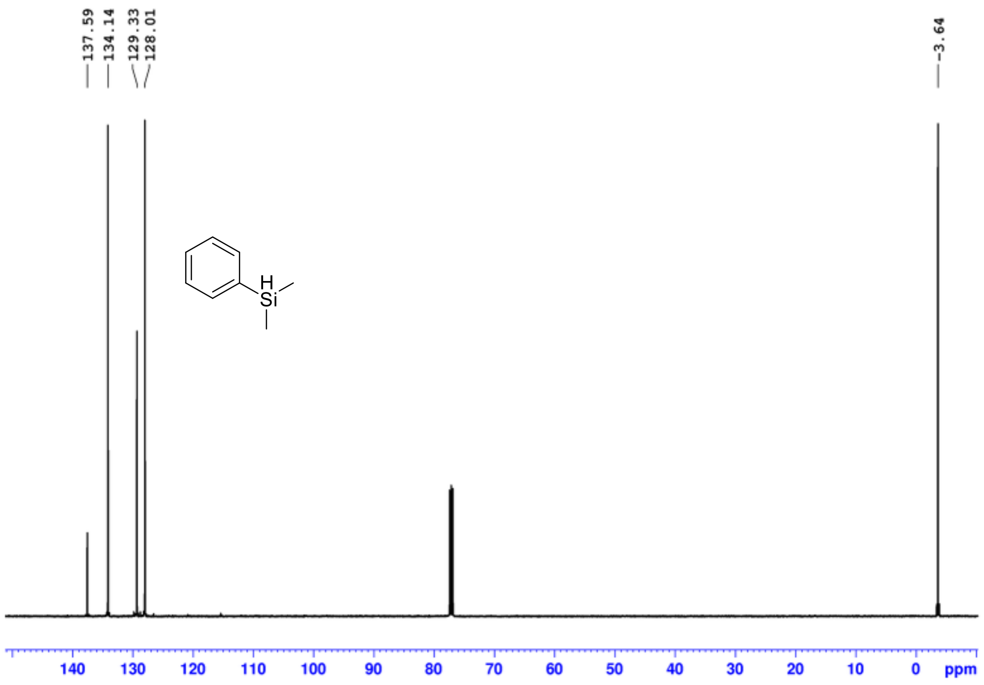

Figure S10. $^{13}C\{^{1}H\}$ **NMR spectrum of** $PhMe_2SiH$

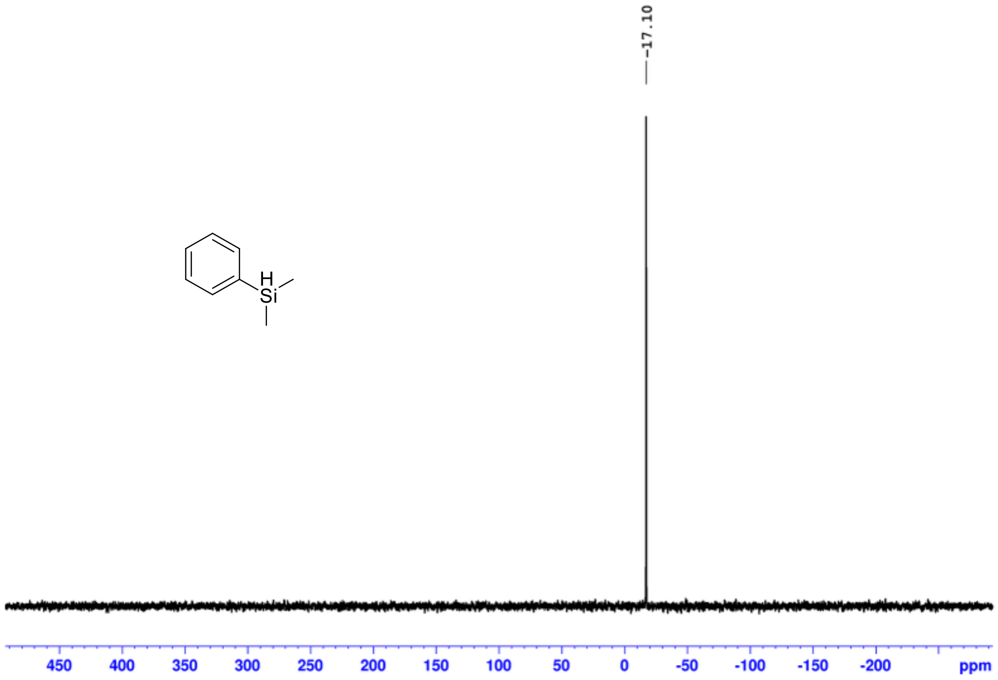

Figure S11. $^{29}Si\{^1H\}$ NMR spectrum of $PhMe_2SiH$

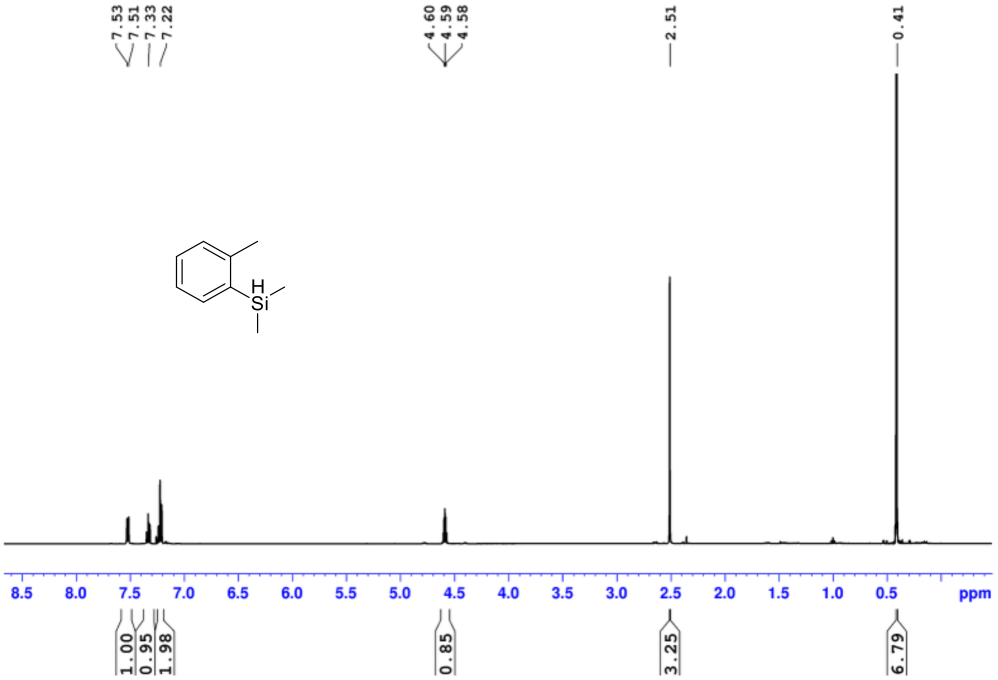

Figure S12. ^1H NMR spectrum of (2-tolyl)Me_2SiH

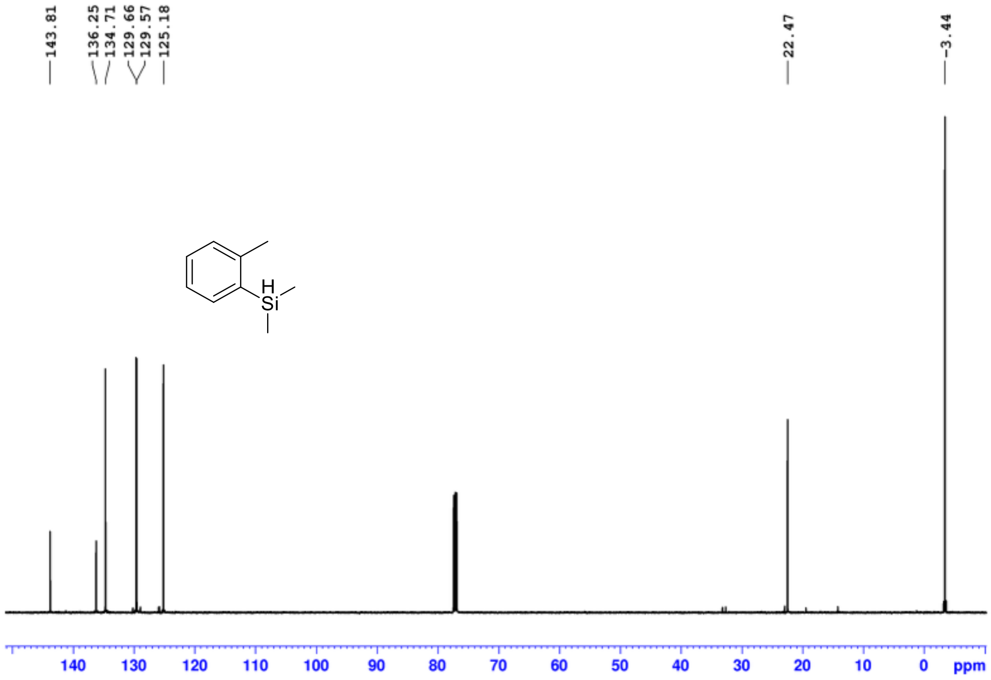

Figure S13. $^{13}C\{^{1}H\}$ **NMR spectrum of (2-tolyl)Me$_2$SiH**

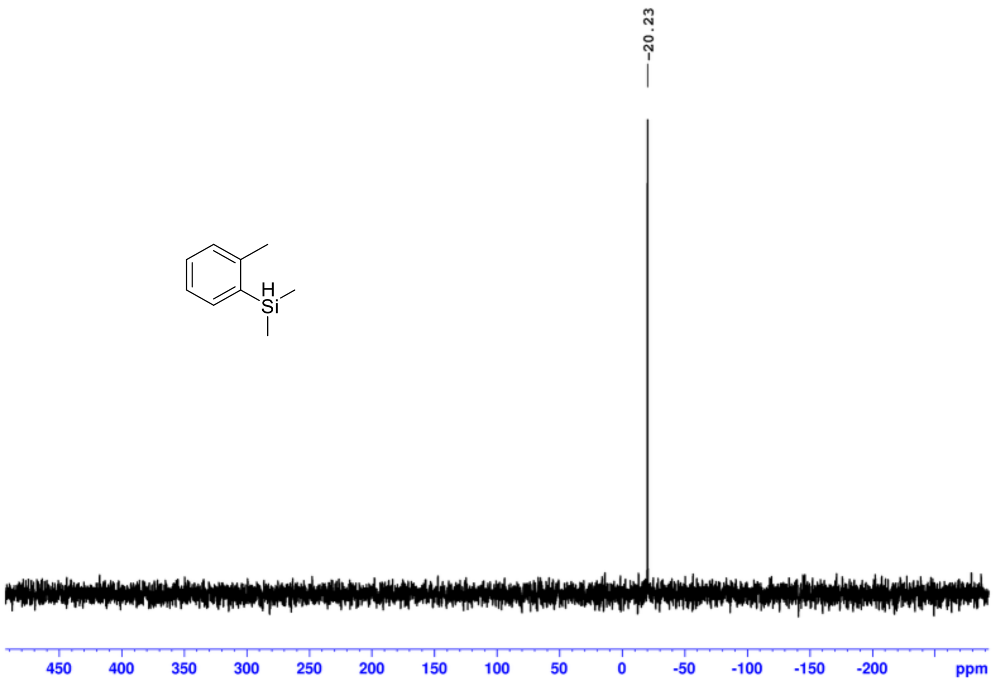

Figure S14. $^{29}Si\{^{1}H\}$ **NMR spectrum of (2-tolyl)Me$_2$SiH**

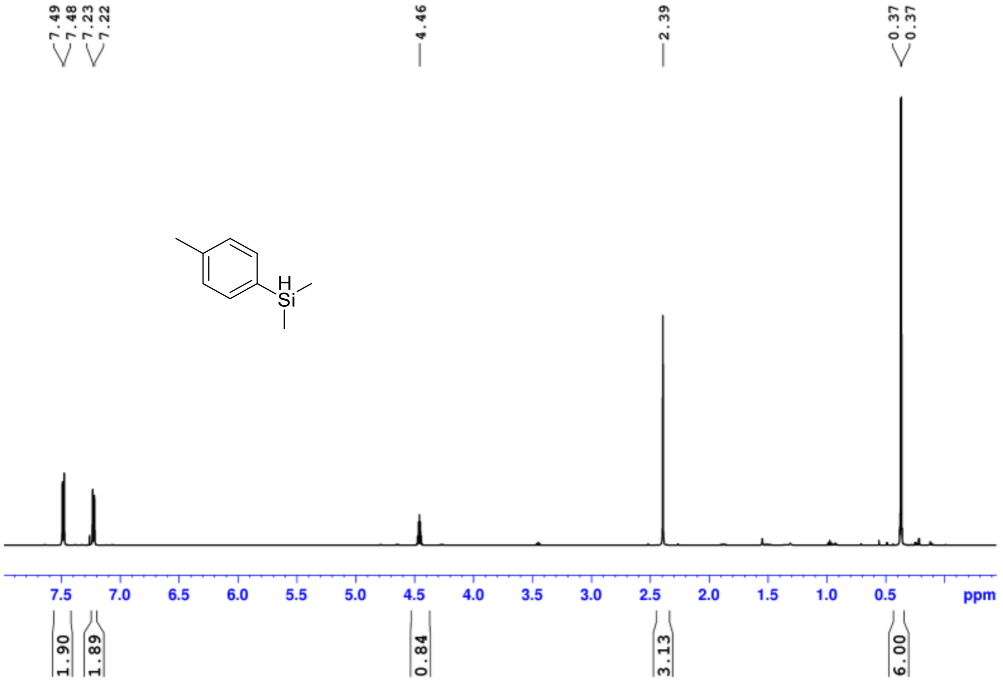

Figure S15. ^{1}H NMR spectrum of (4-tolyl)Me_2SiH

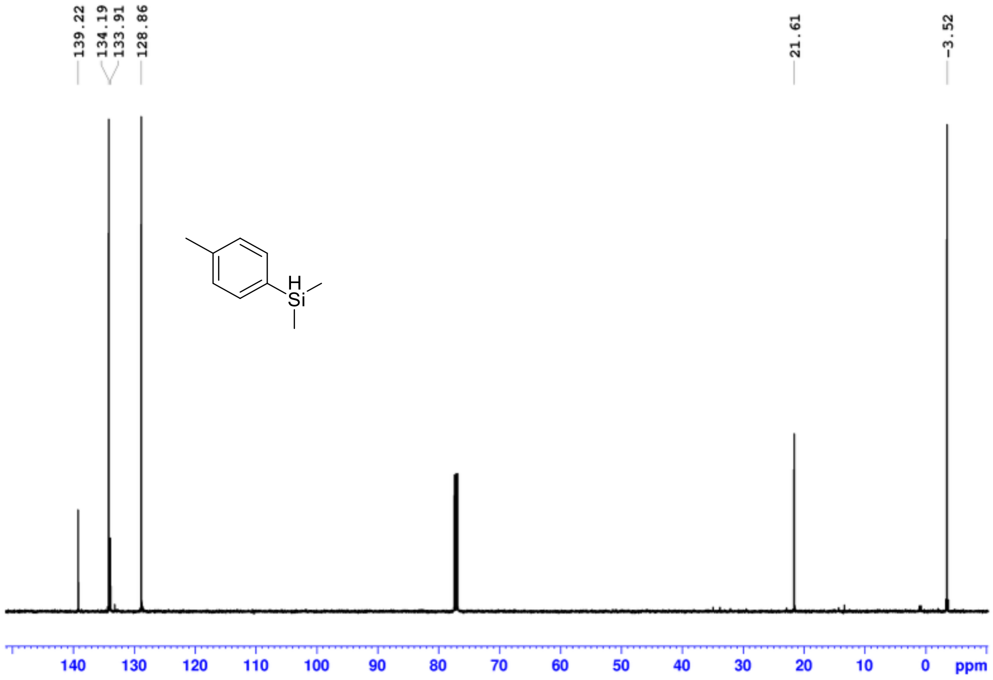

Figure S16. $^{13}C\{^{1}H\}$ NMR spectrum of (4-tolyl)Me_2SiH

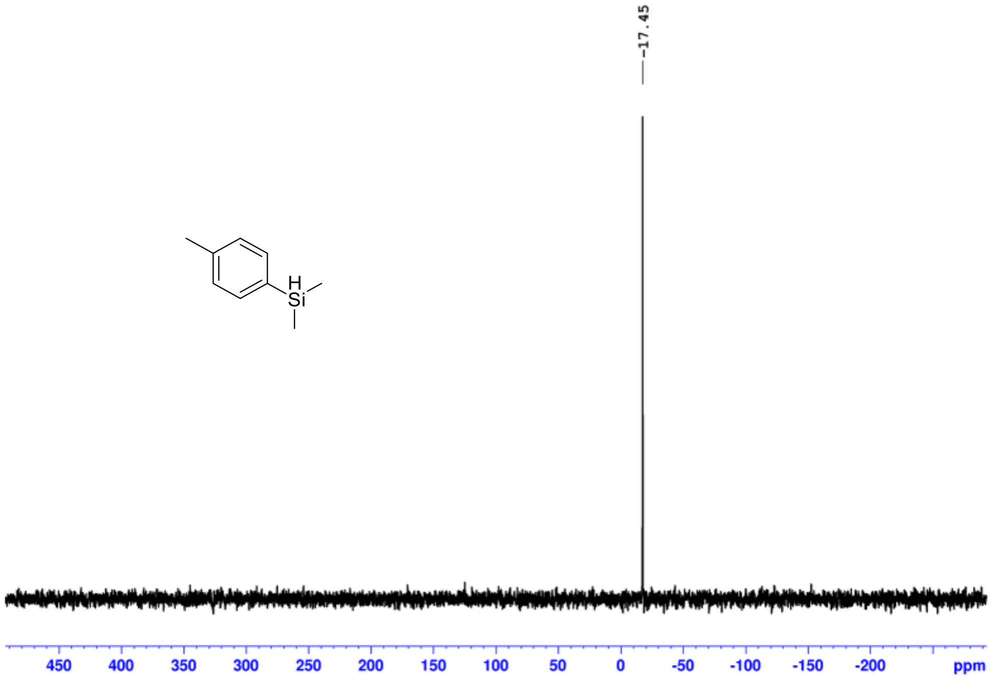

Figure S17. $^{29}Si\{^{1}H\}$ **NMR spectrum of (4-tolyl)Me_2SiH**

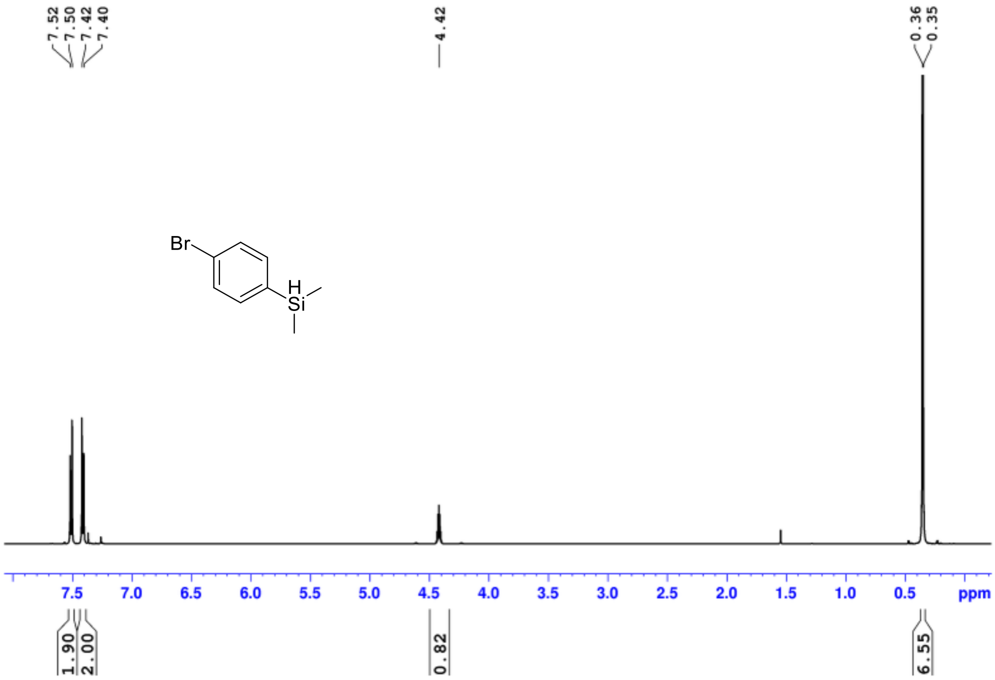

Figure S18. ^{1}H **NMR spectrum of (4-Br-C_6H_4)Me_2SiH**

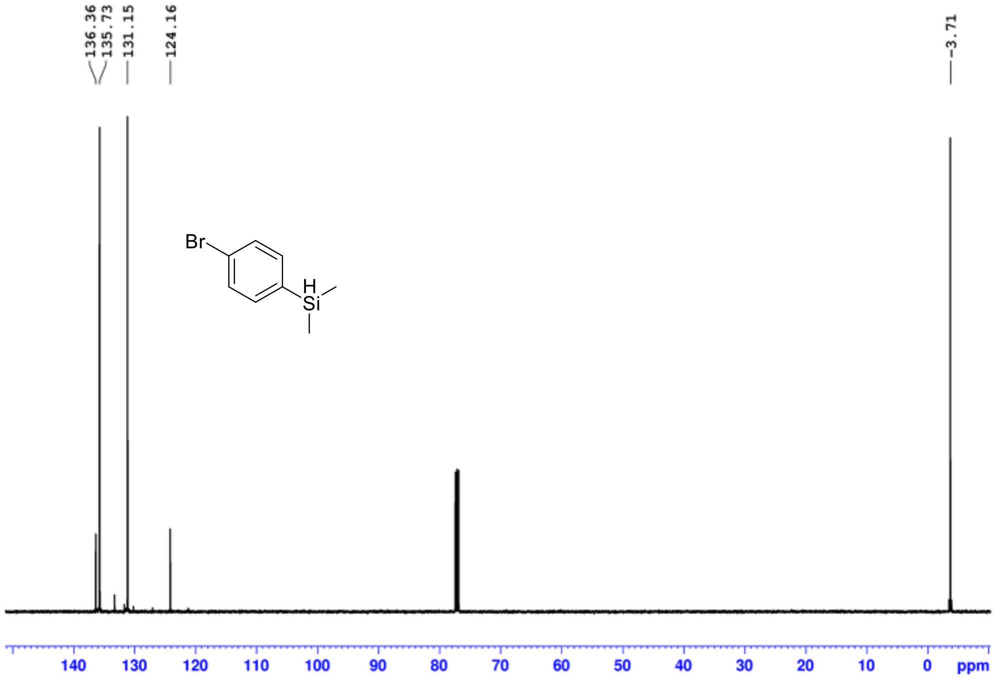

Figure S19. $^{13}C\{^1H\}$ NMR spectrum of (4-Br-C_6H_4)Me_2SiH

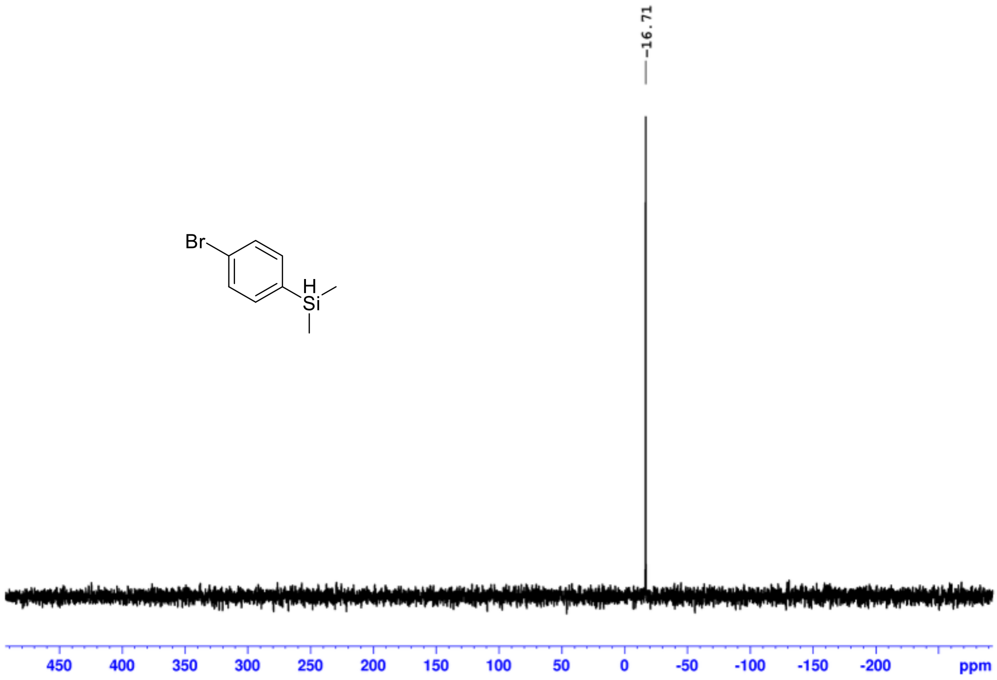

Figure S20. $^{29}Si\{^1H\}$ NMR spectrum of (4-Br-C_6H_4)Me_2SiH

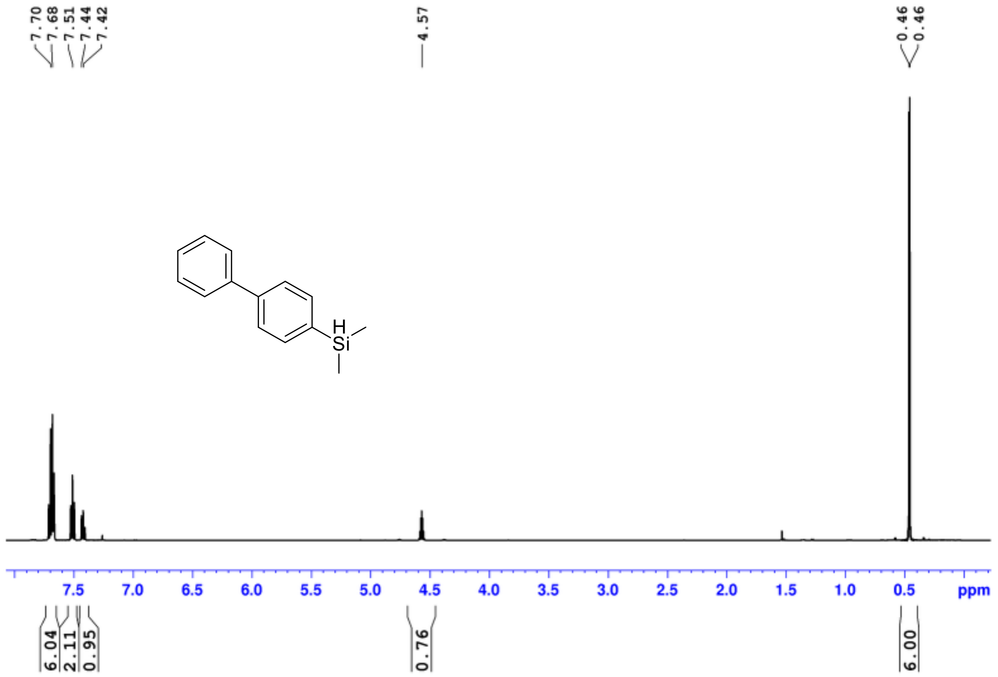

Figure S21. ^{1}H NMR spectrum of (biphenyl-4-yl)Me_2SiH

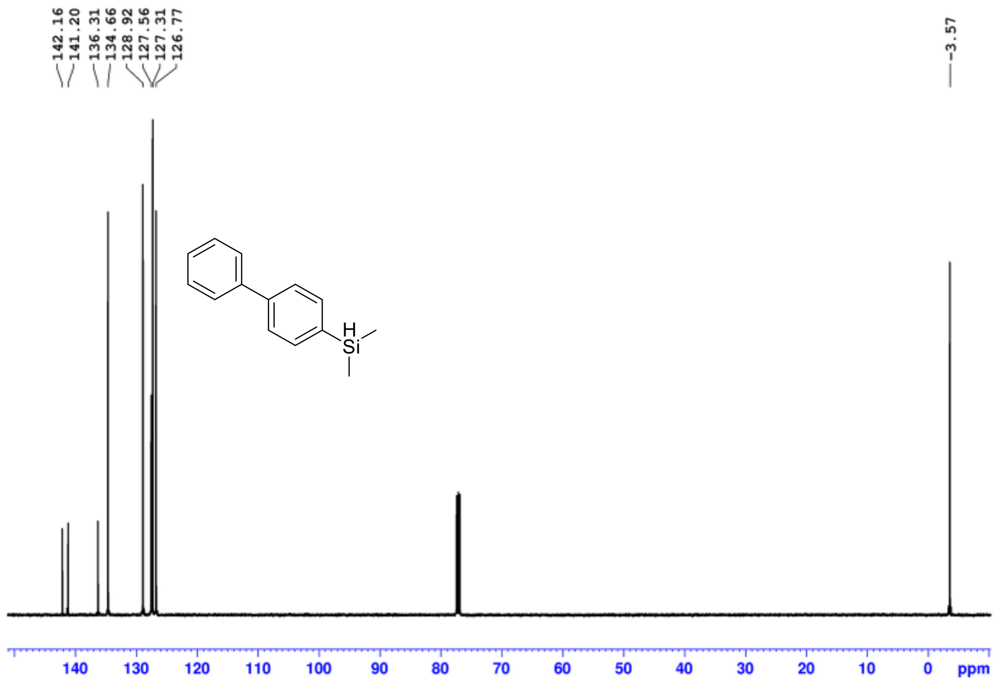

Figure S22. ^{13}C{^{1}H} NMR spectrum of (biphenyl-4-yl)Me_2SiH

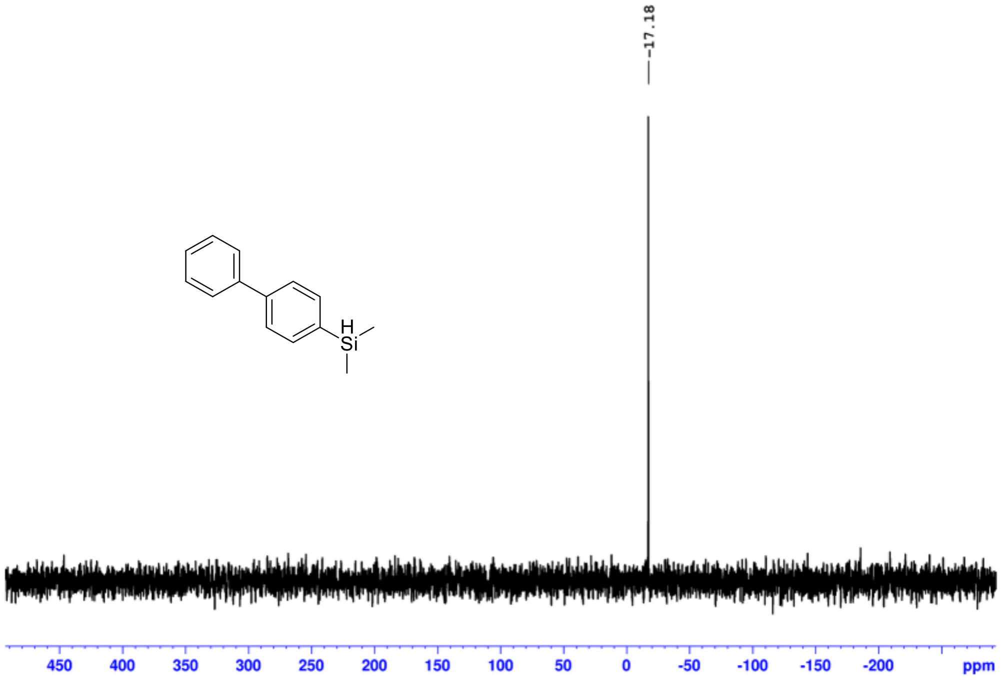

Figure S23. $^{29}Si\{^1H\}$ **NMR spectrum of (biphenyl-4-yl)Me$_2$SiH**

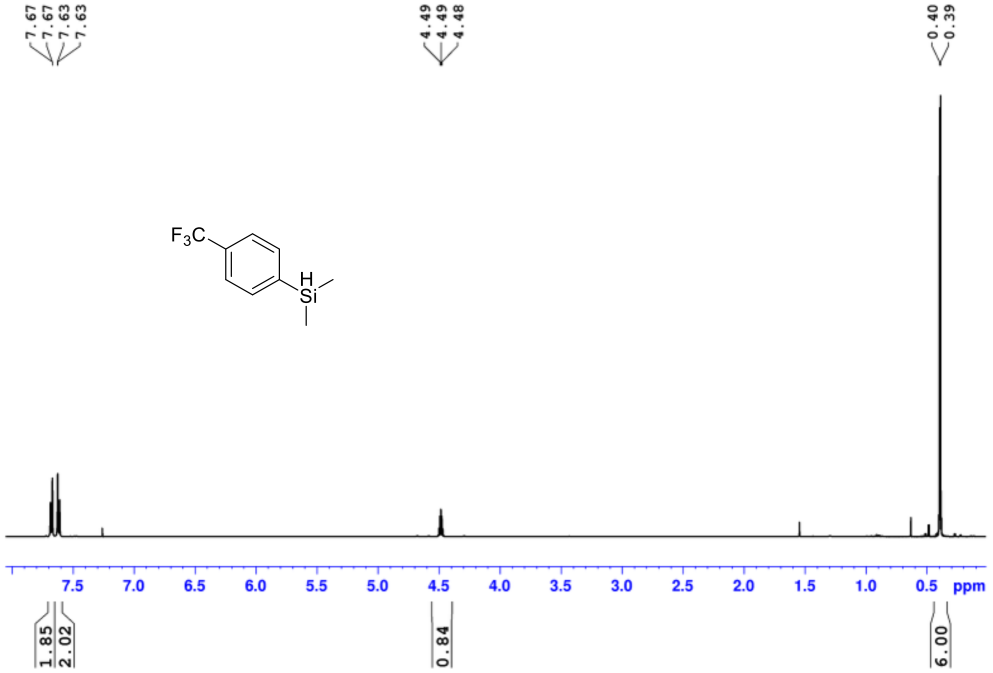

Figure S24. ^1H **NMR spectrum of (4-CF$_3$-C$_6$H$_4$)Me$_2$SiH**

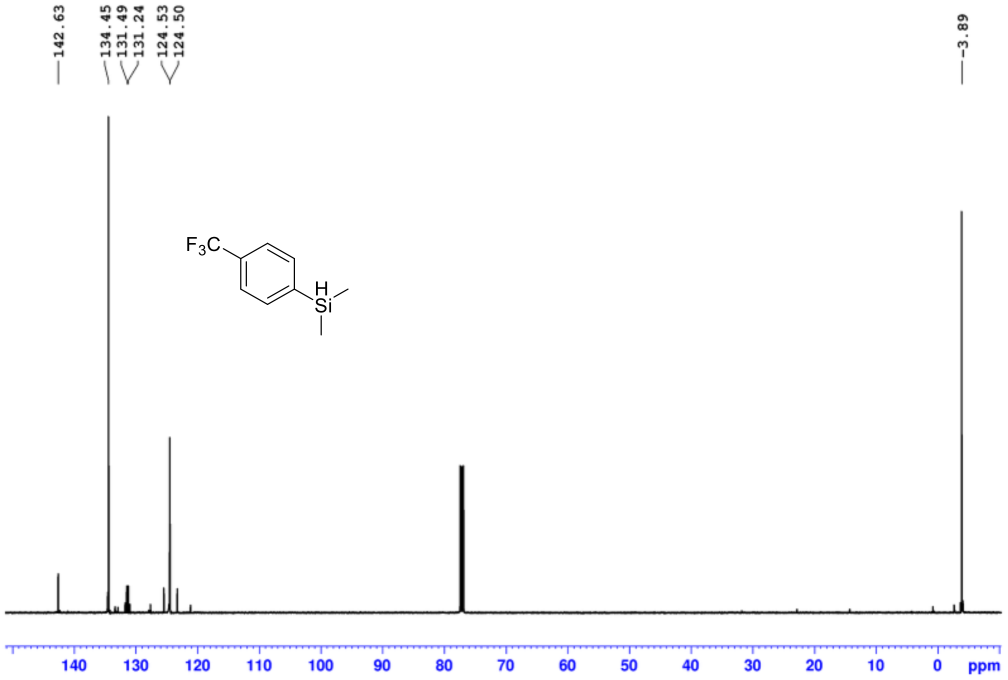

Figure S25. $^{13}C\{^{1}H\}$ NMR spectrum of $(4\text{-}CF_3\text{-}C_6H_4)Me_2SiH$

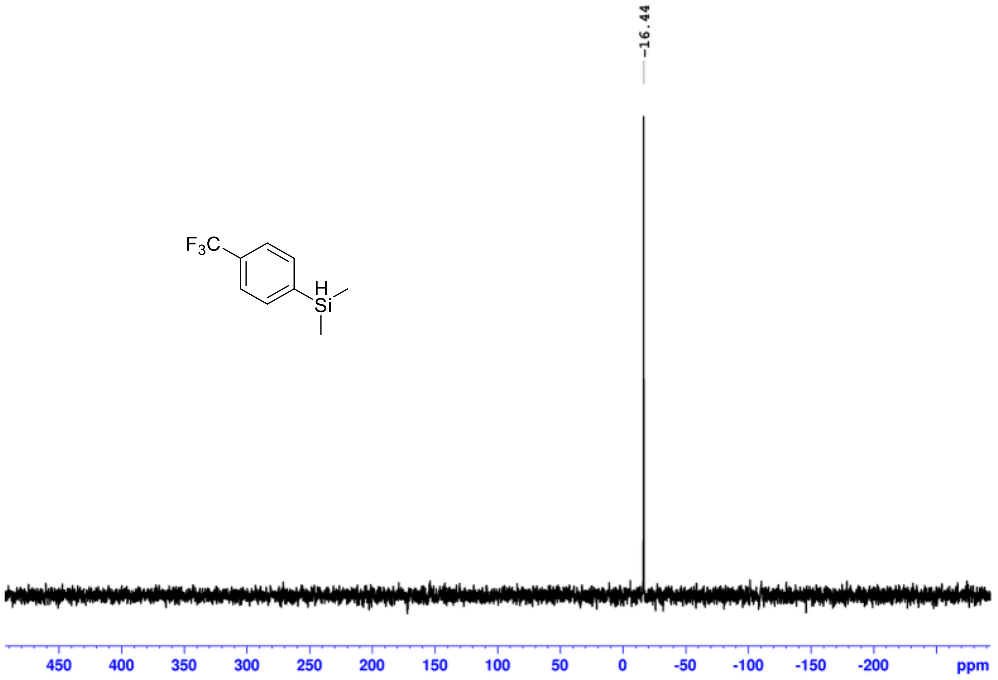

Figure S26. $^{29}Si\{^{1}H\}$ NMR spectrum of $(4\text{-}CF_3\text{-}C_6H_4)Me_2SiH$

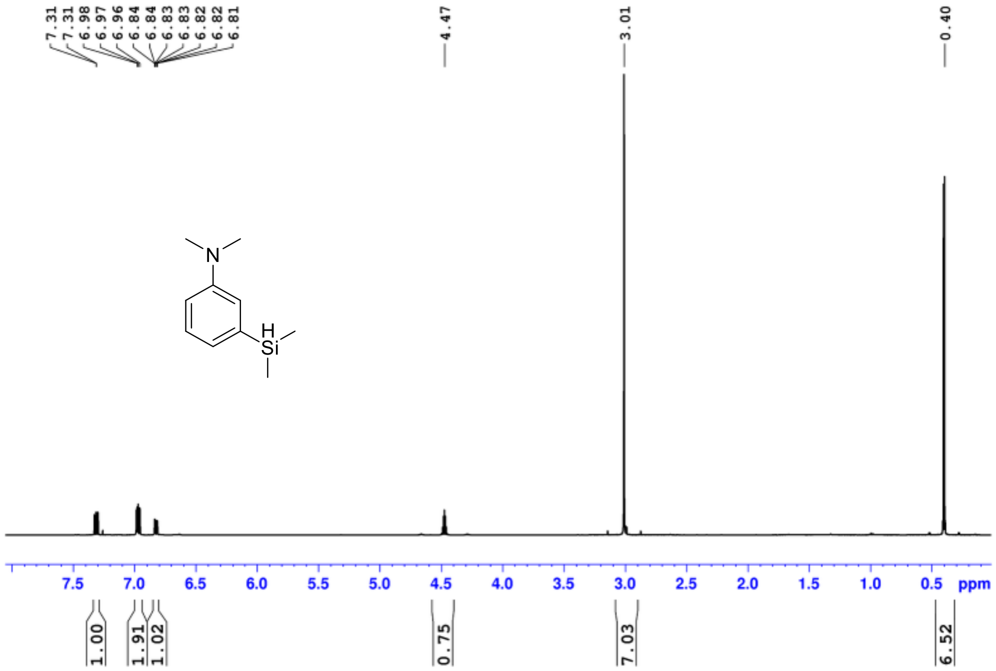

Figure S27. ^{1}H **NMR spectrum of (3-NMe$_2$-C$_6$H$_4$)Me$_2$SiH**

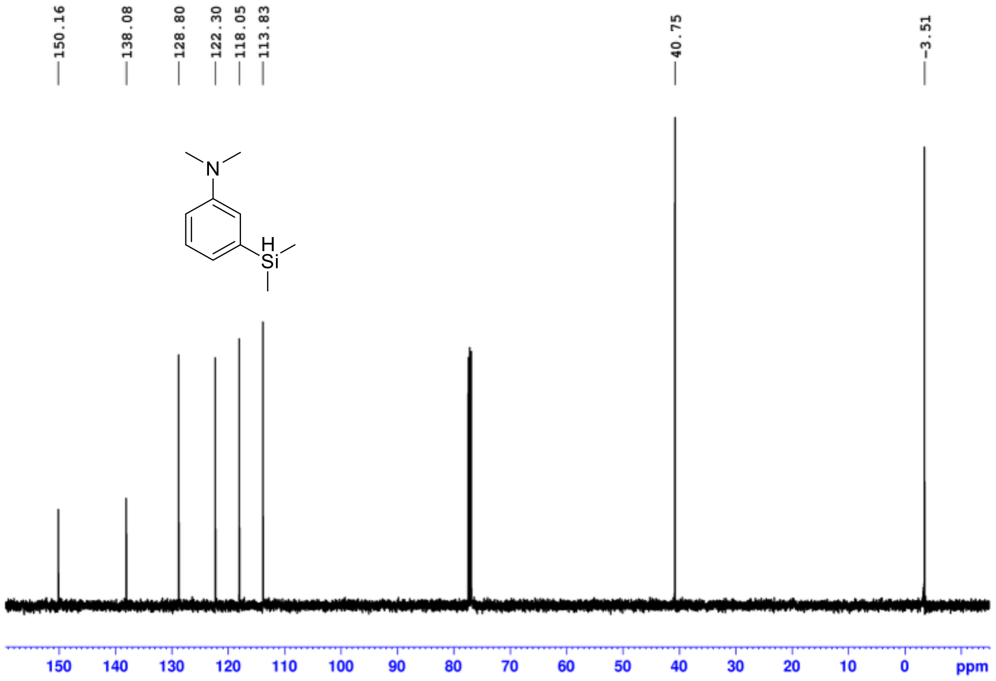

Figure S28. $^{13}C\{^{1}H\}$ **NMR spectrum of (3-NMe$_2$-C$_6$H$_4$)Me$_2$SiH**

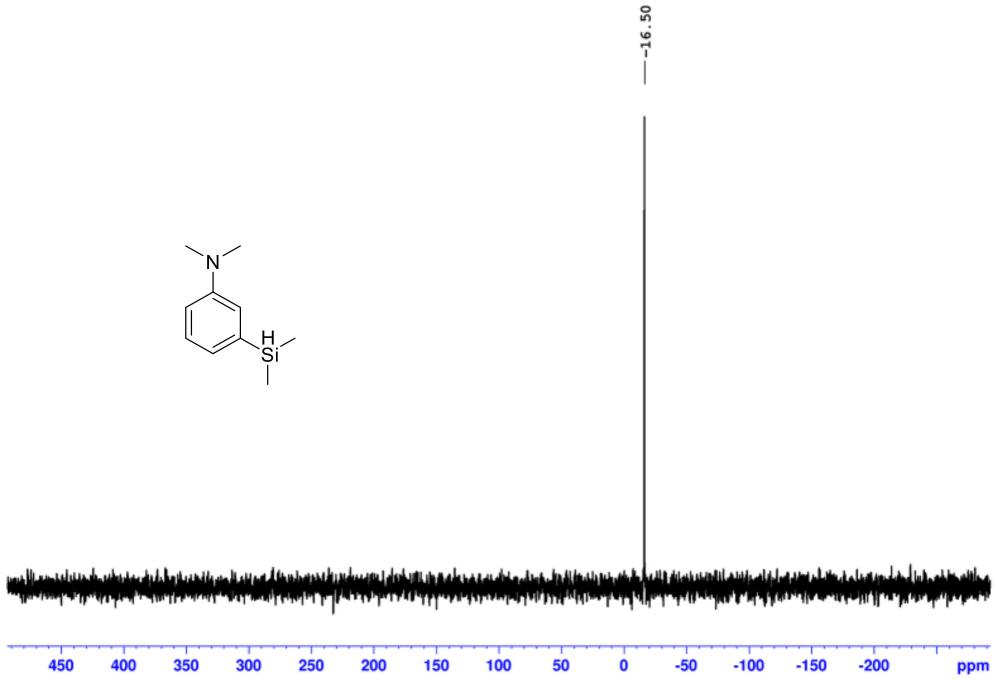

Figure S29. $^{29}Si\{^{1}H\}$ **NMR spectrum of (3-NMe$_2$-C$_6$H$_4$)Me$_2$SiH**

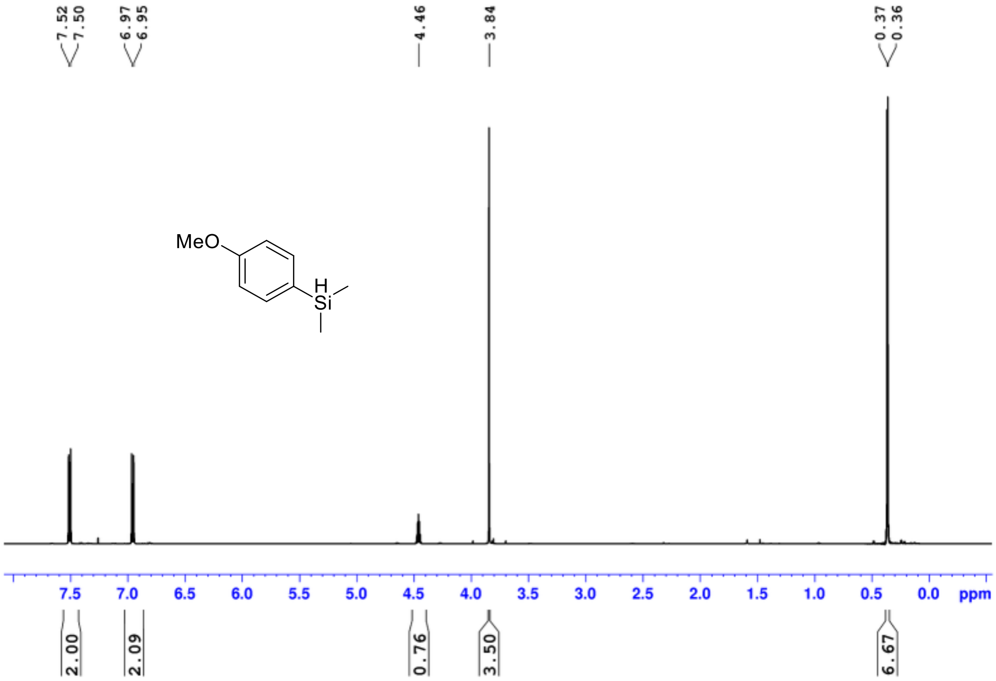

Figure S30. ^{1}H **NMR spectrum of (4-MeO-C$_6$H$_4$)Me$_2$SiH**

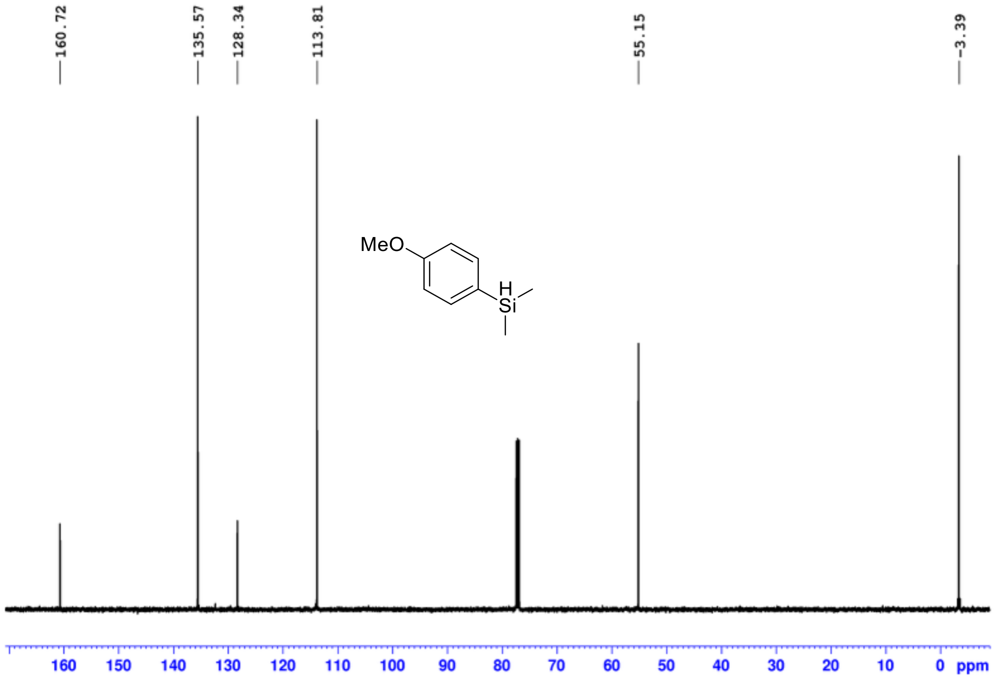

Figure S31. $^{13}C\{^{1}H\}$ NMR spectrum of (4-MeO-C_6H_4)Me_2SiH

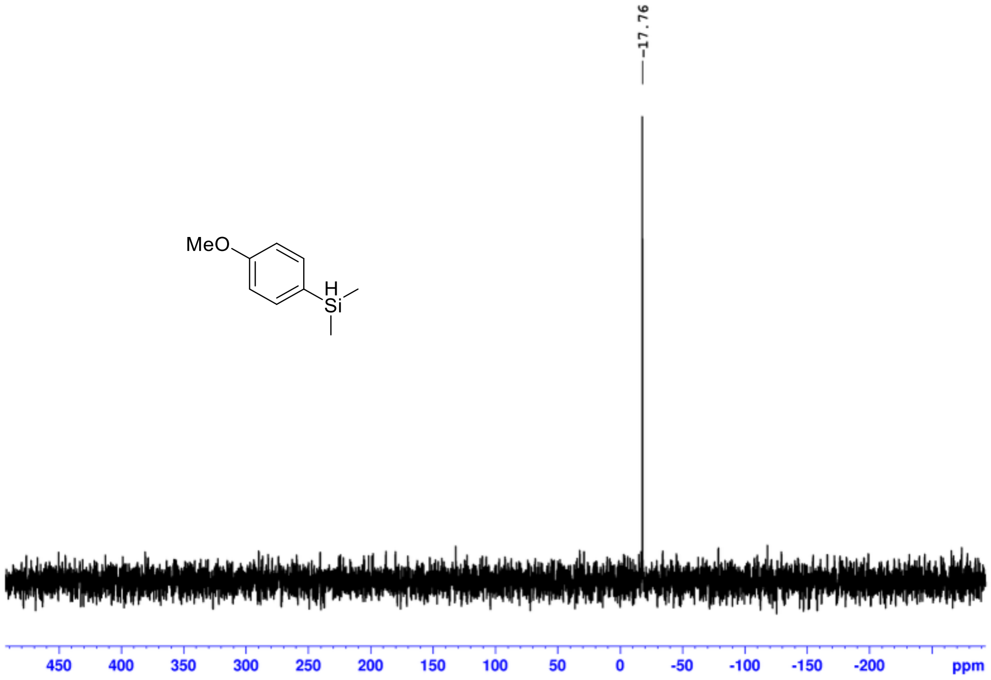

Figure S32. $^{29}Si\{^{1}H\}$ NMR spectrum of (4-MeO-C_6H_4)Me_2SiH

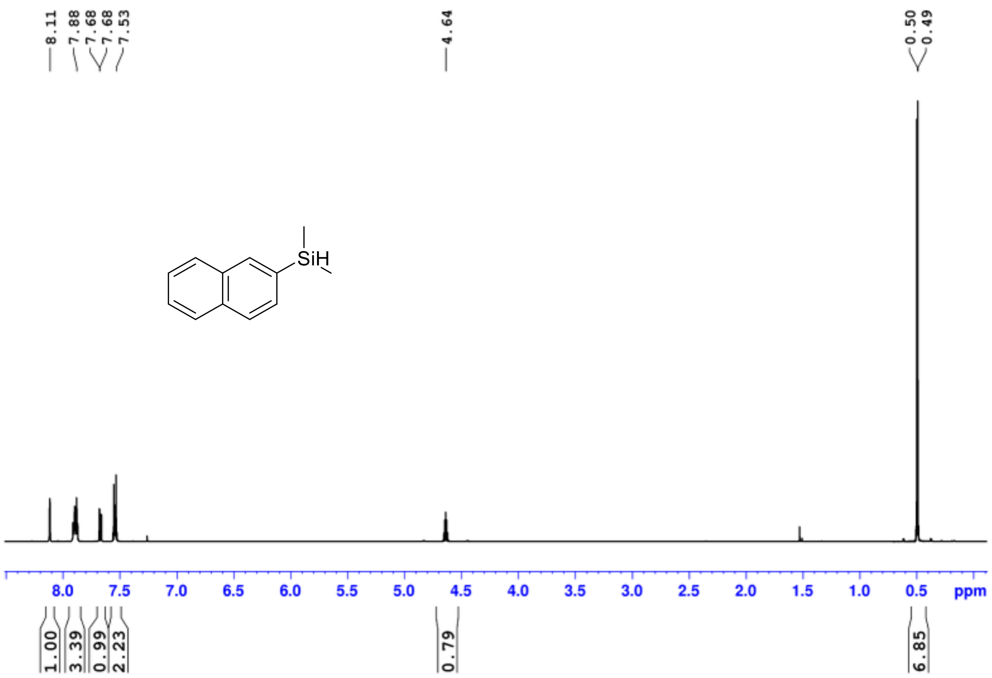

Figure S33. ^{1}H NMR spectrum of (naphthalen-2-yl)Me_2SiH

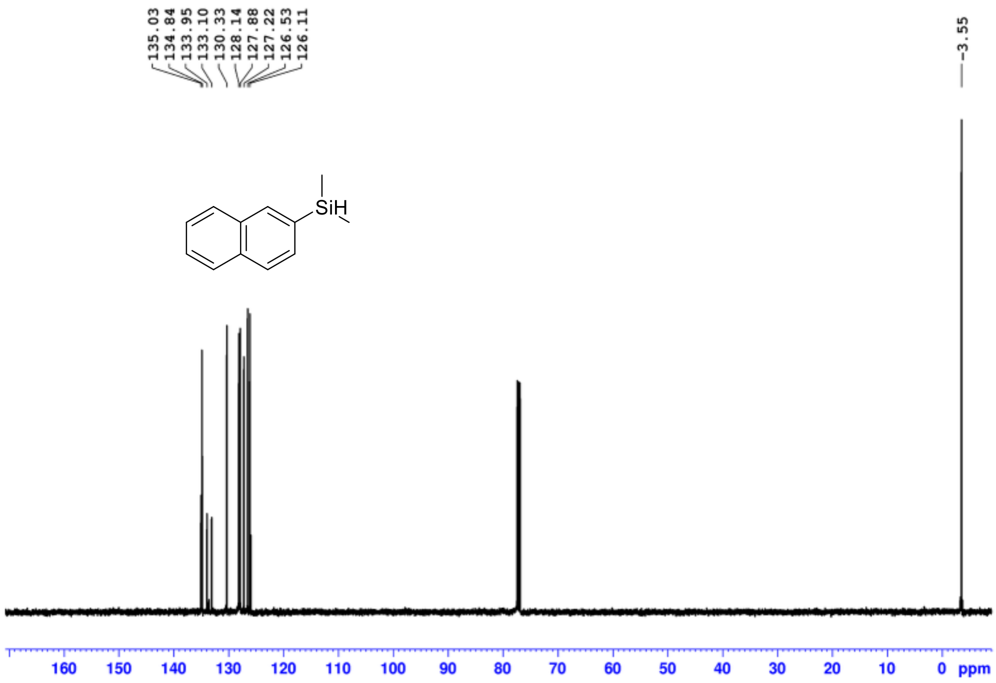

Figure S34. $^{13}C\{^1H\}$ NMR spectrum of (naphthalen-2-yl)Me_2SiH

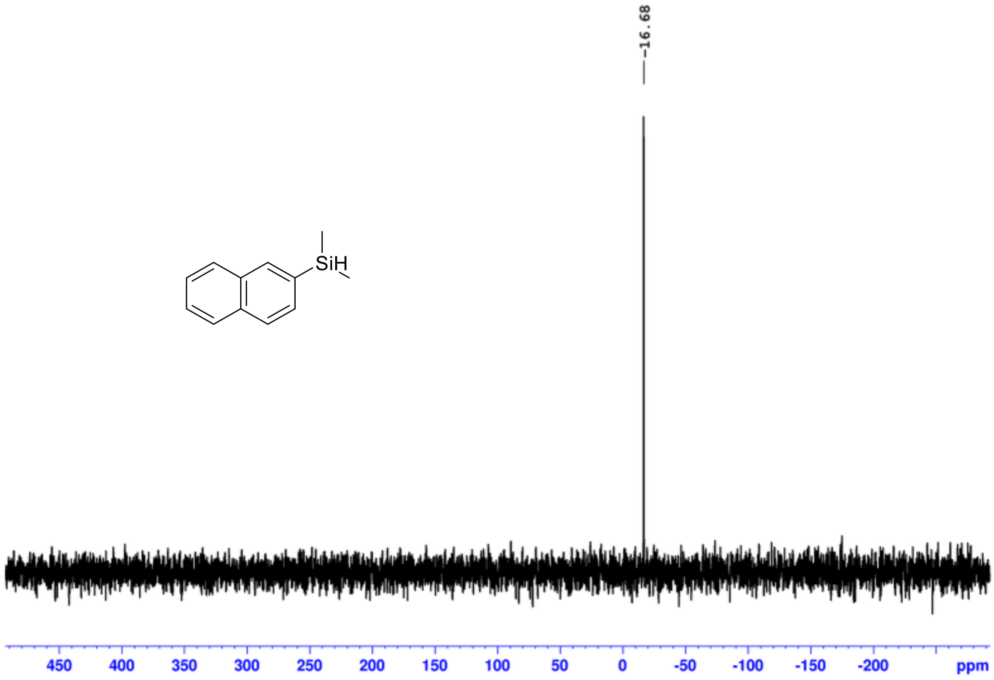

Figure S35. $^{29}Si\{^1H\}$ NMR spectrum of (naphthalen-2-yl)Me_2SiH

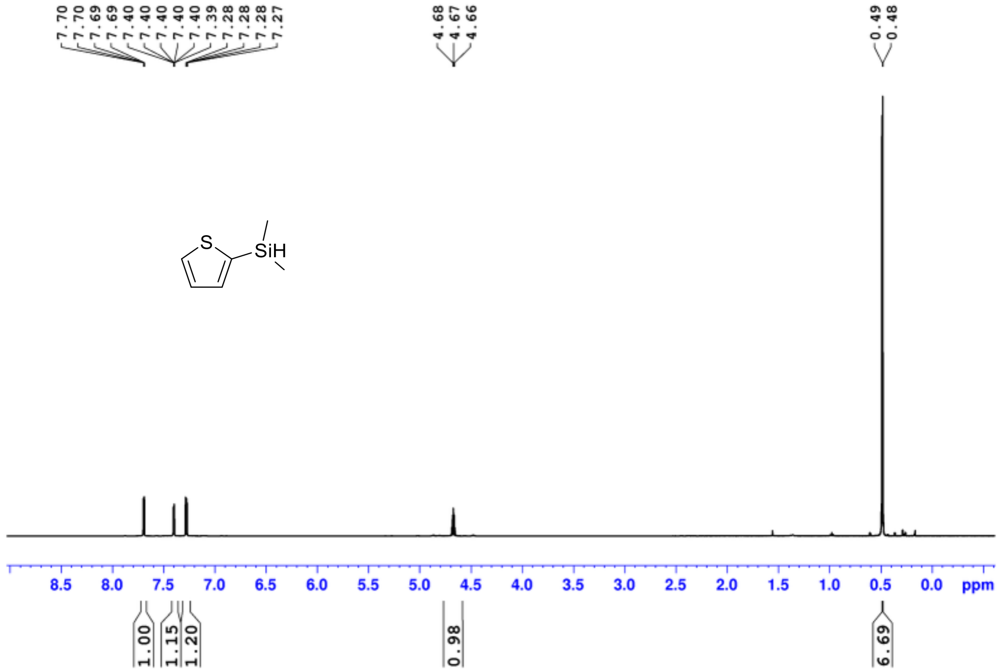

Figure S36. ^1H NMR spectrum of (thiophen-2-yl)Me_2SiH

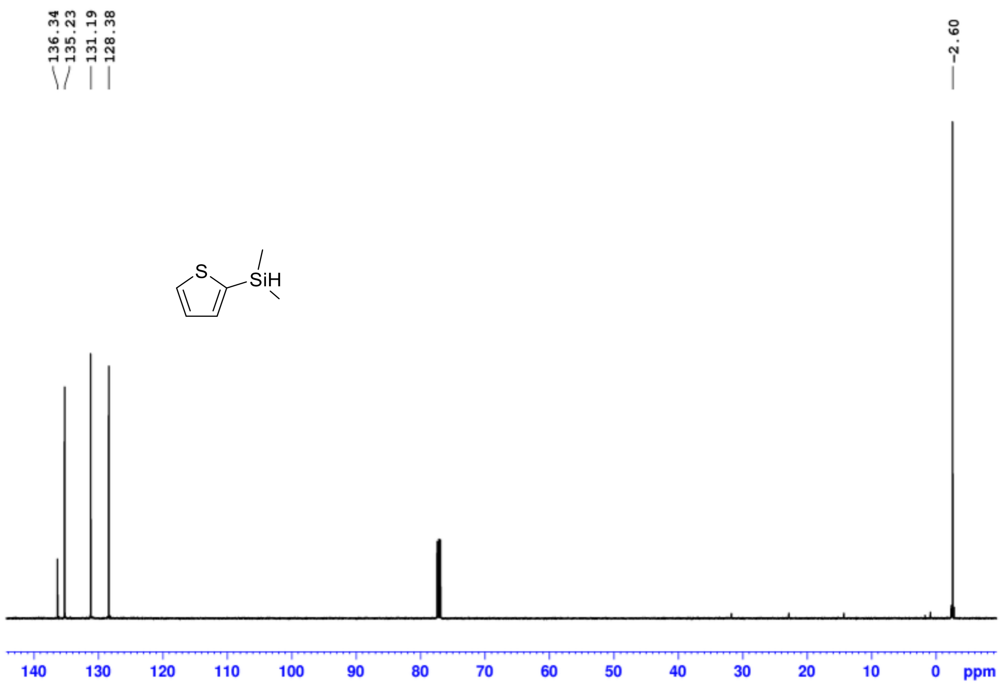

Figure S37. $^{13}C\{^{1}H\}$ NMR spectrum of (thiophen-2-yl)Me_2SiH

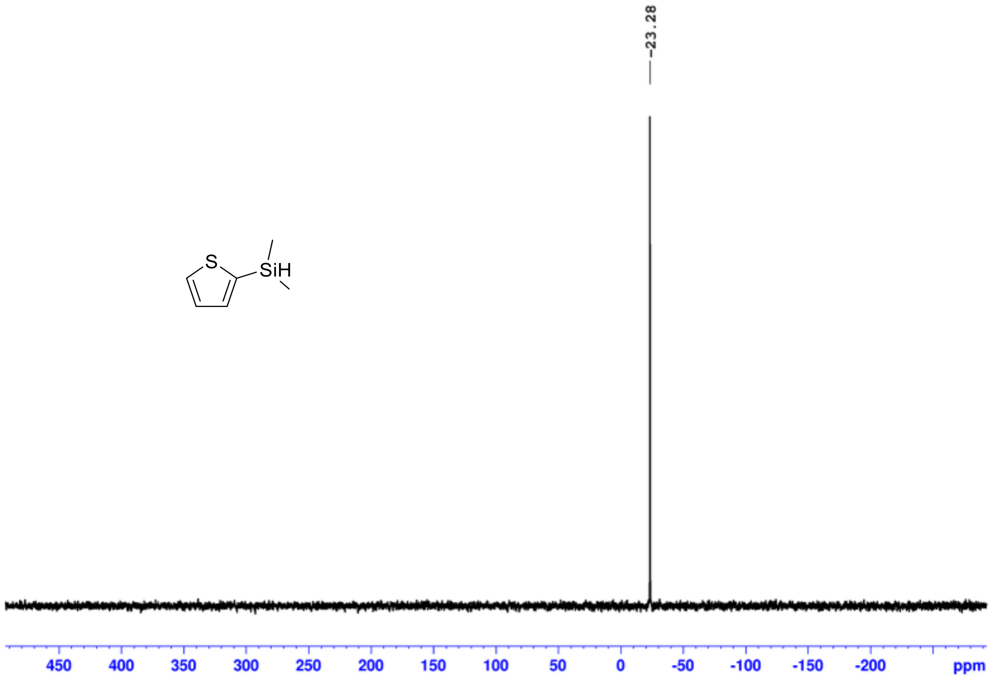

Figure S38. $^{29}Si\{^{1}H\}$ NMR spectrum of (thiophen-2-yl)Me_2SiH

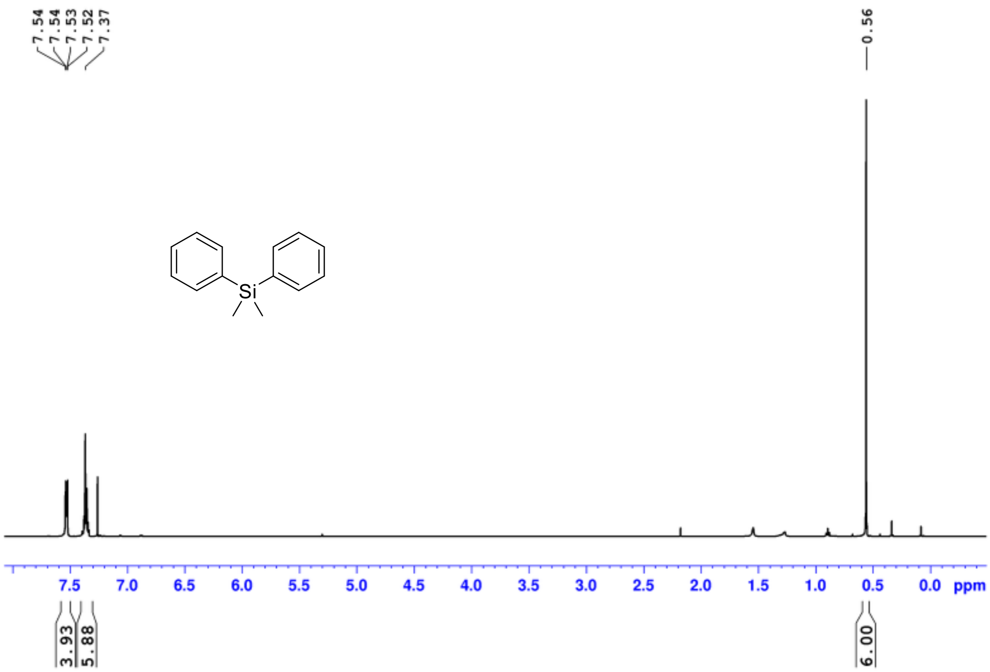

Figure S39. **^{1}H NMR spectrum of Ph_2Me_2Si**

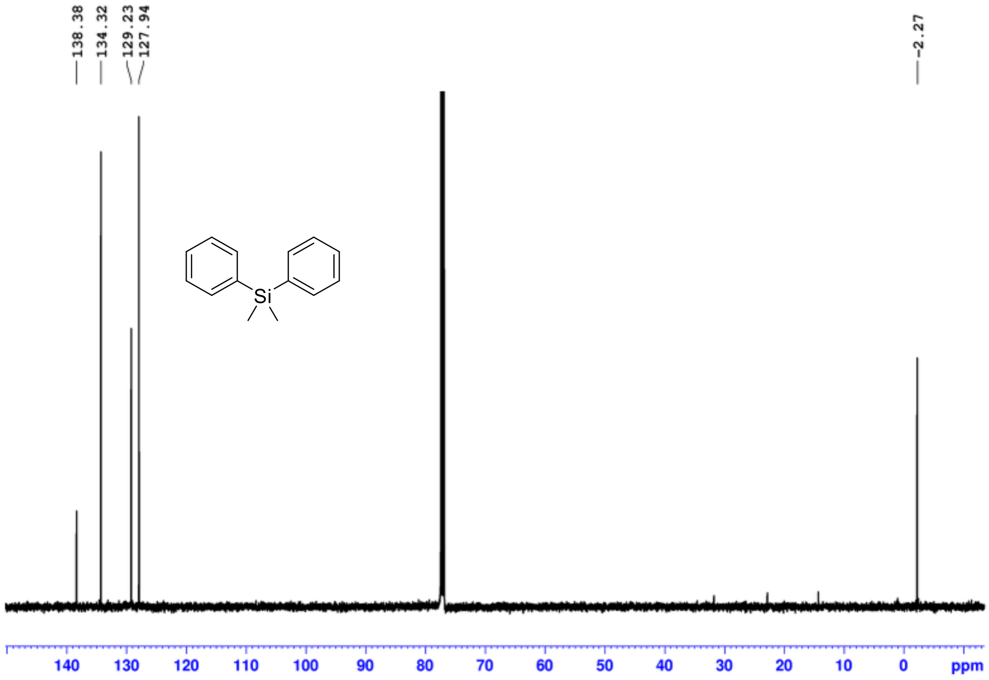

Figure S40. **^{13}C{^{1}H} NMR spectrum of Ph_2Me_2Si**

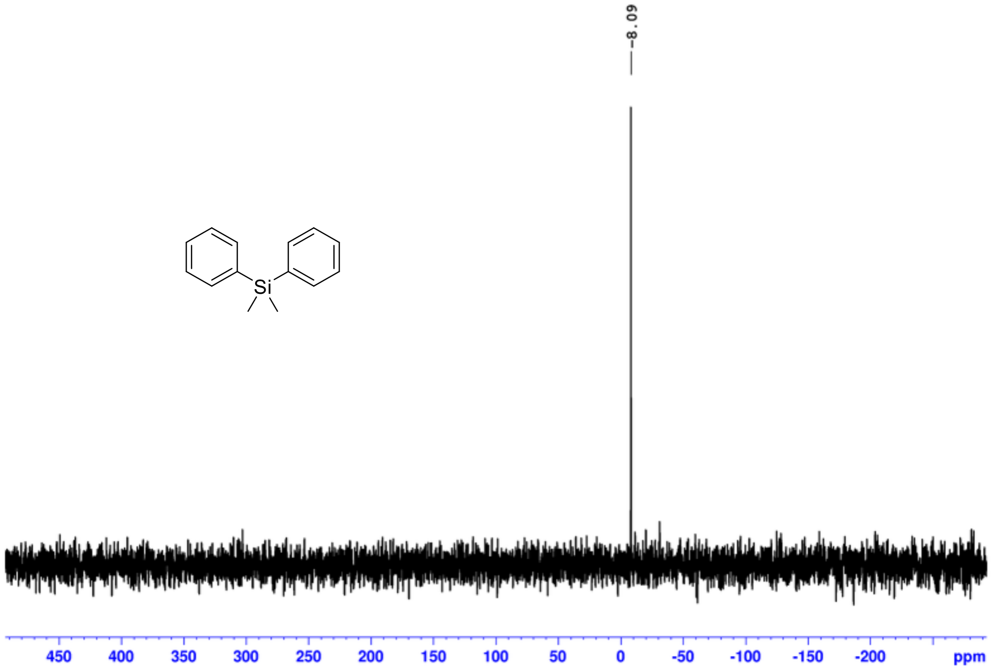

Figure S41. $^{29}Si\{^1H\}$ NMR spectrum of Ph_2Me_2Si

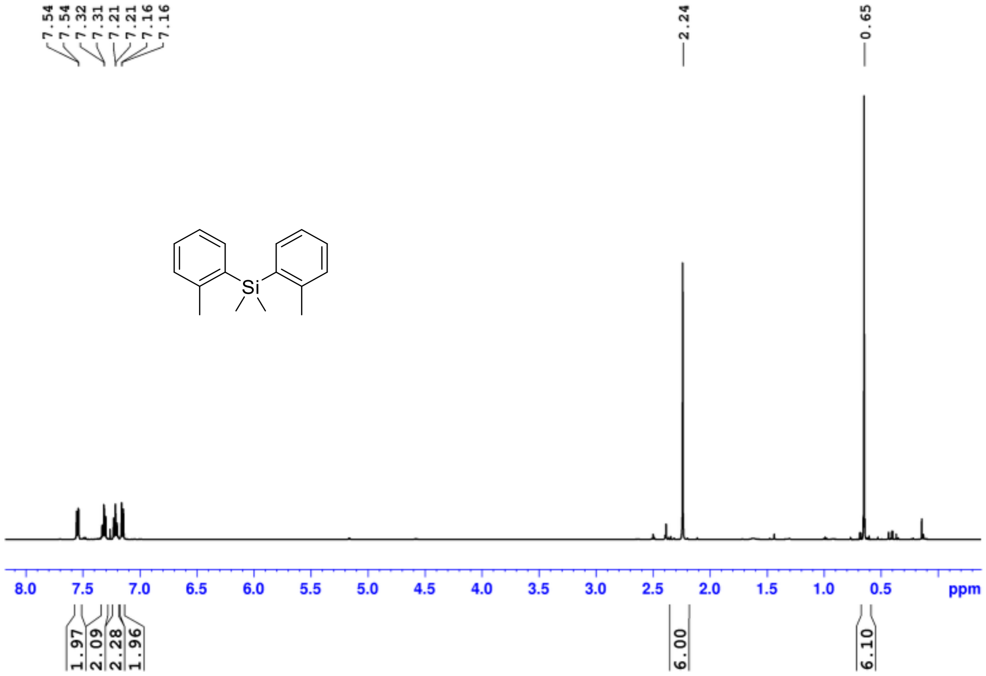

Figure S42. ^1H NMR spectrum of (2-tolyl)$_2$Me$_2$Si

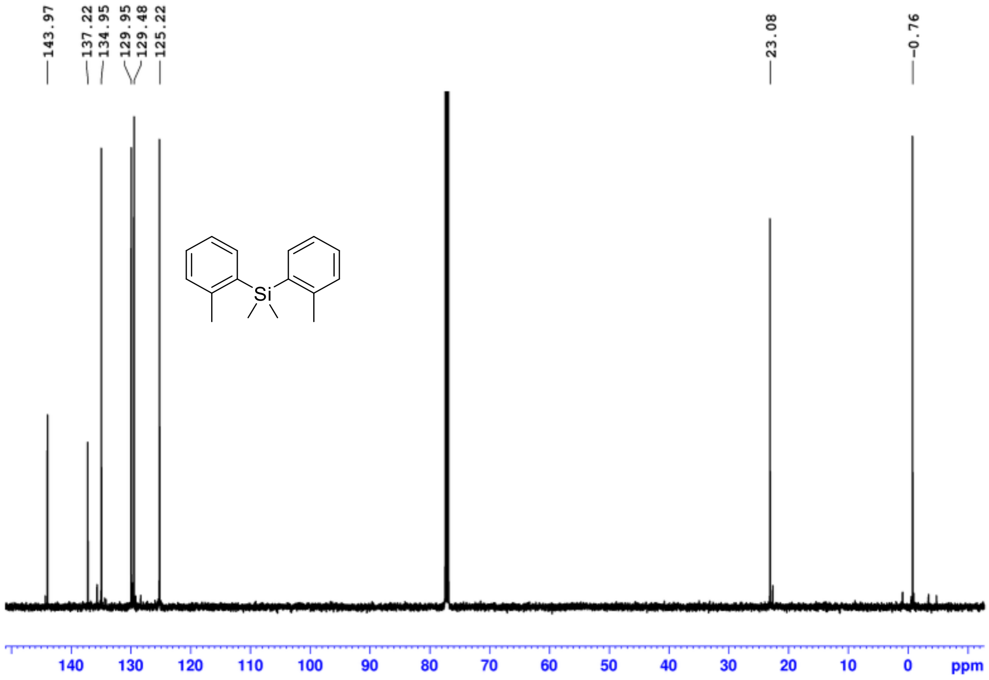

Figure S43. $^{13}C\{^1H\}$ NMR spectrum of $(2\text{-tolyl})_2Me_2Si$

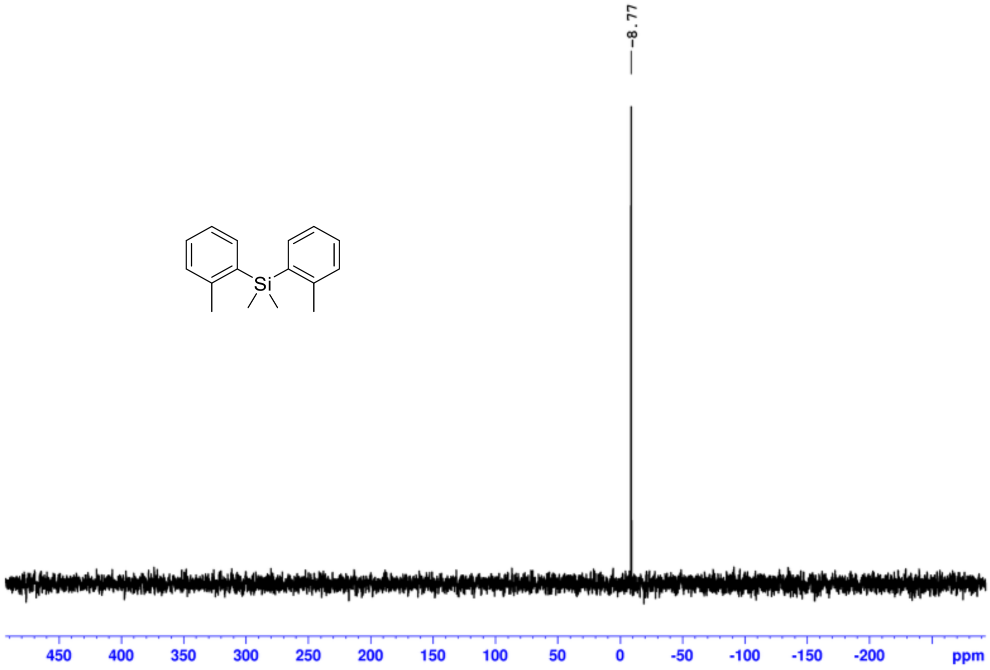

Figure S44. $^{29}Si\{^1H\}$ NMR spectrum of $(2\text{-tolyl})_2Me_2Si$

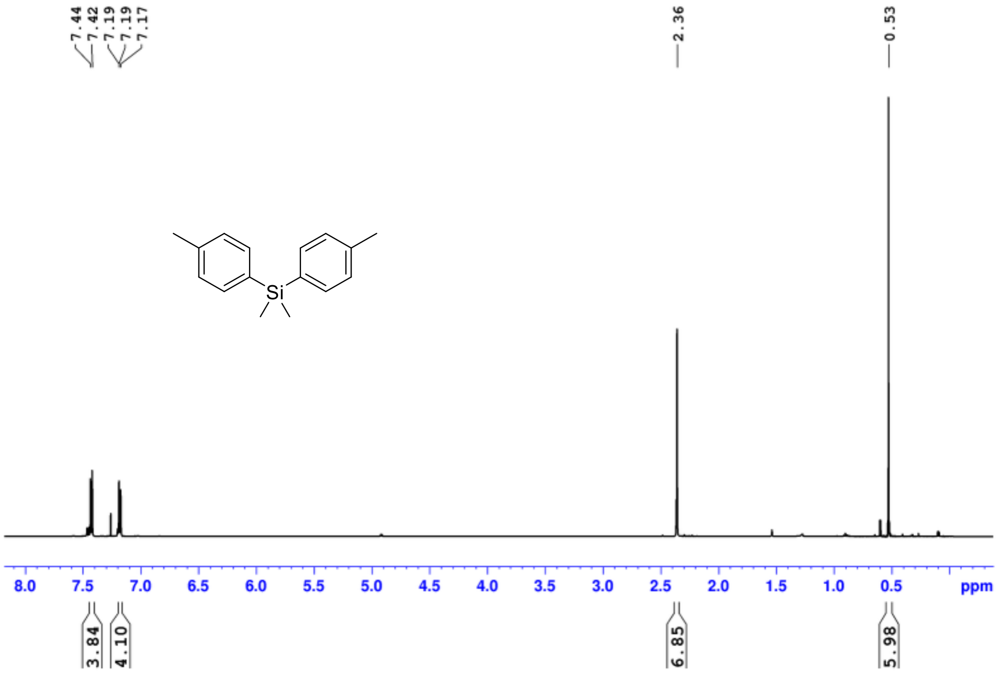

Figure S45. ^{1}H **NMR spectrum of (4-tolyl)$_2$Me$_2$Si**

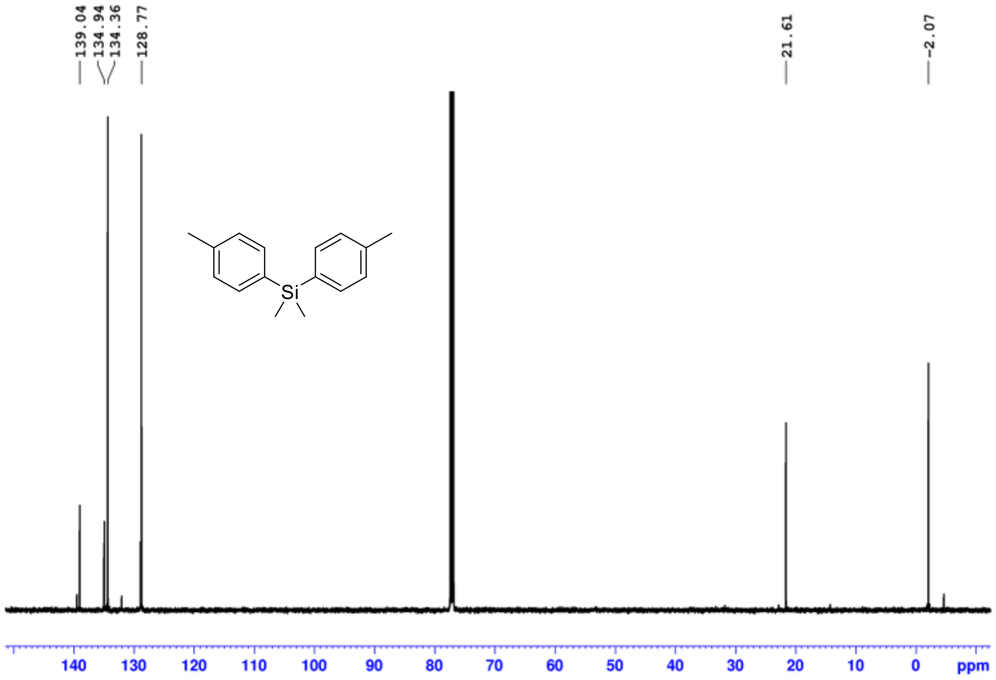

Figure S46. $^{13}C\{^{1}H\}$ **NMR spectrum of (4-tolyl)$_2$Me$_2$Si**

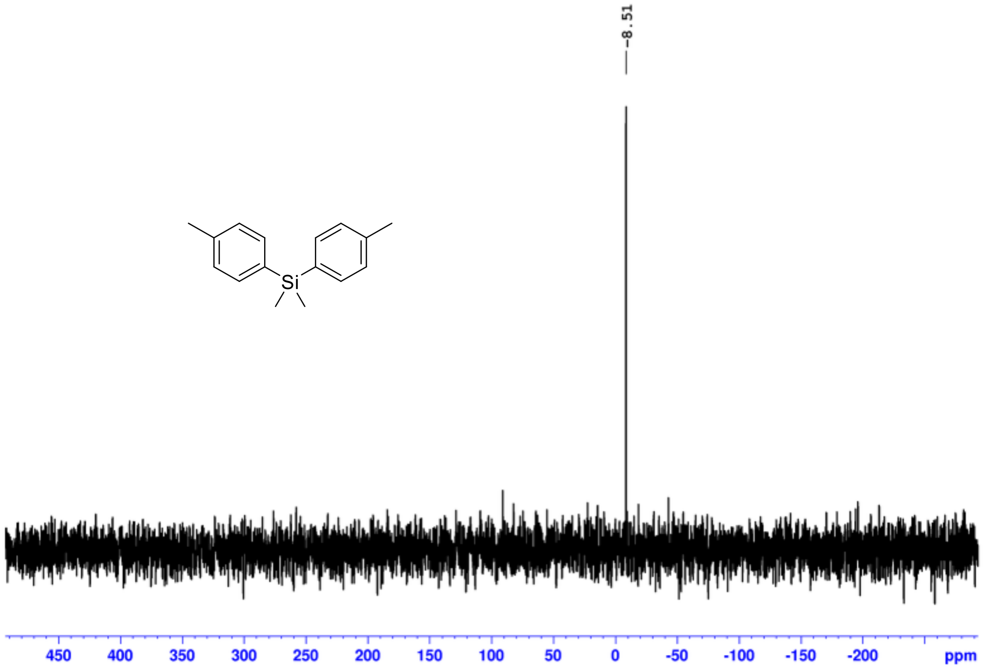

Figure S47. $^{29}Si\{^{1}H\}$ **NMR spectrum of (4-tolyl)$_2$Me$_2$Si**

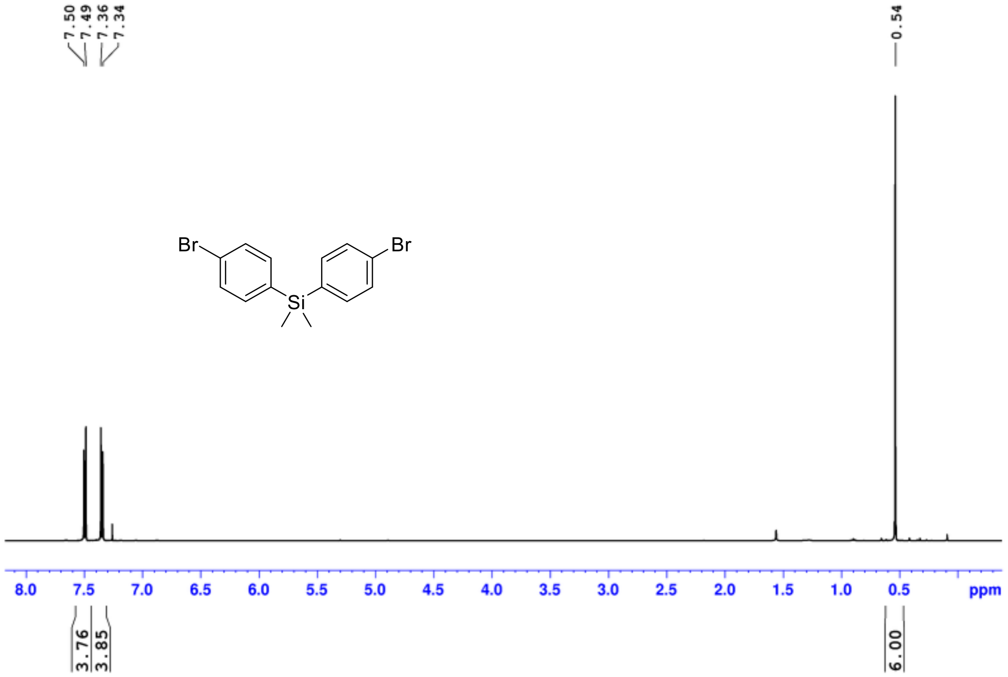

Figure S48. ^{1}H **NMR spectrum of (4-Br-C$_6$H$_4$)$_2$Me$_2$Si**

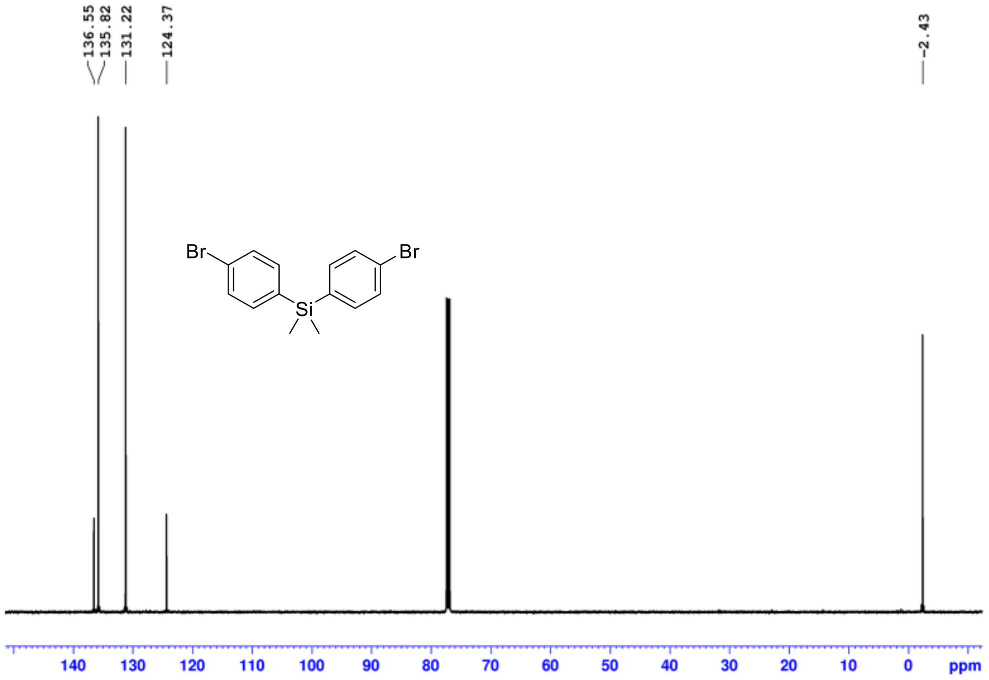

Figure S49. $^{13}C\{^{1}H\}$ NMR spectrum of $(4\text{-Br-}C_6H_4)_2Me_2Si$

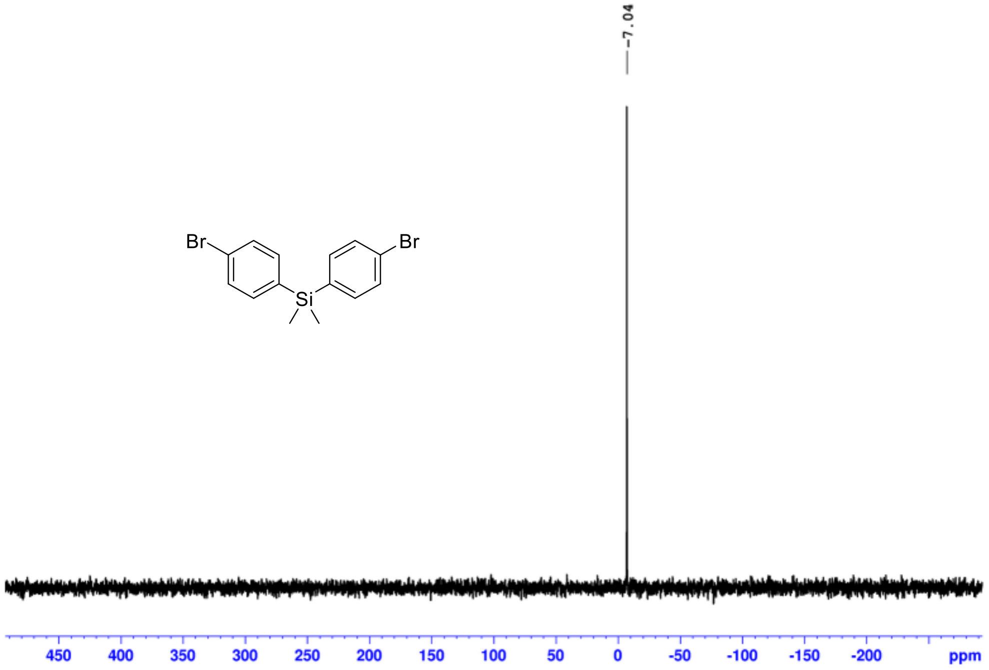

Figure S50. $^{29}Si\{^{1}H\}$ NMR spectrum of $(4\text{-Br-}C_6H_4)_2Me_2Si$

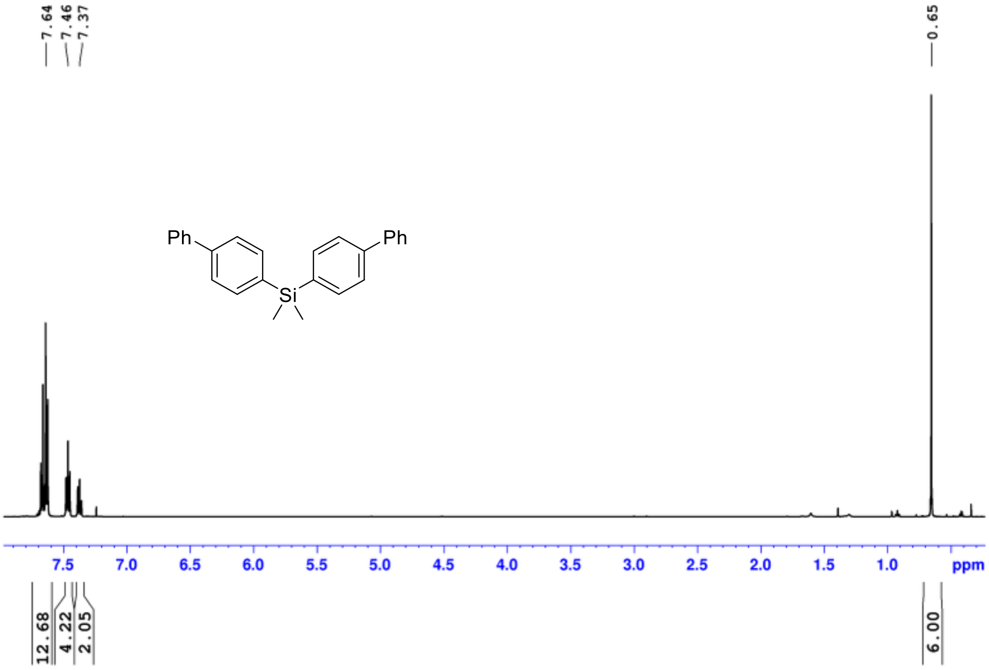

Figure S51. ^{1}H NMR spectrum of (biphenyl-4-yl)$_2$Me$_2$Si

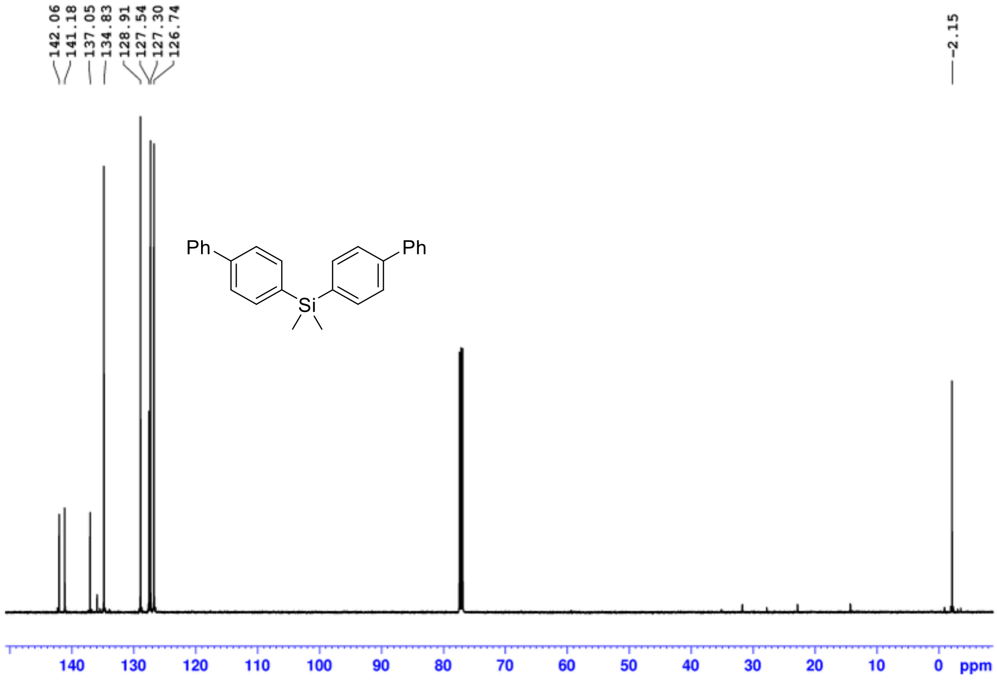

Figure S52. $^{13}C\{^{1}H\}$ NMR spectrum of (biphenyl-4-yl)$_2$Me$_2$Si

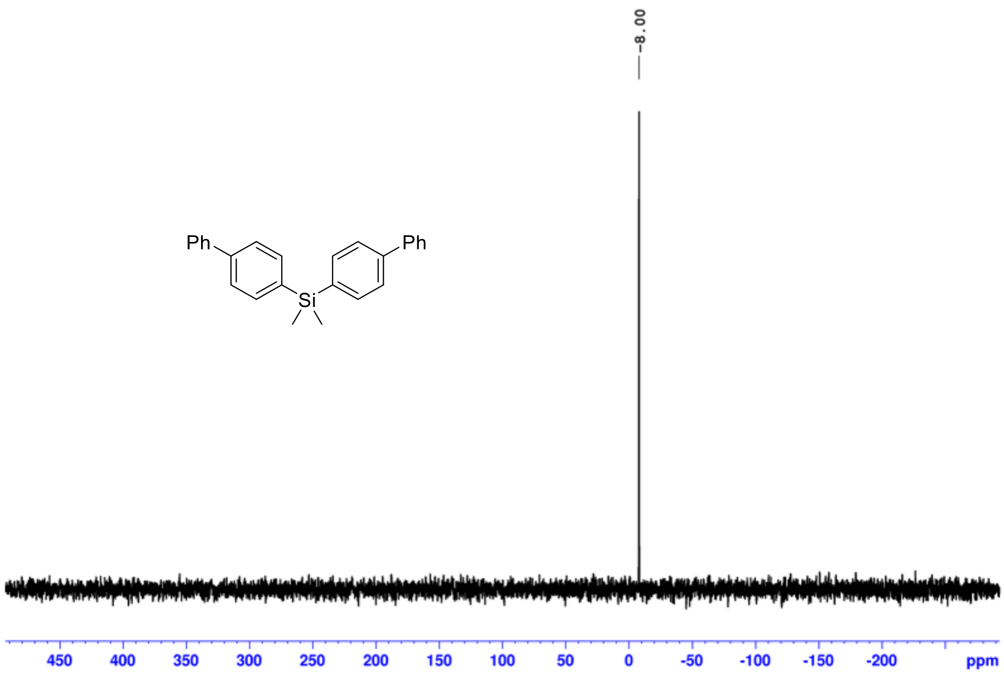

Figure S53. $^{29}Si\{^{1}H\}$ NMR spectrum of (biphenyl-4-yl)$_2$Me$_2$Si

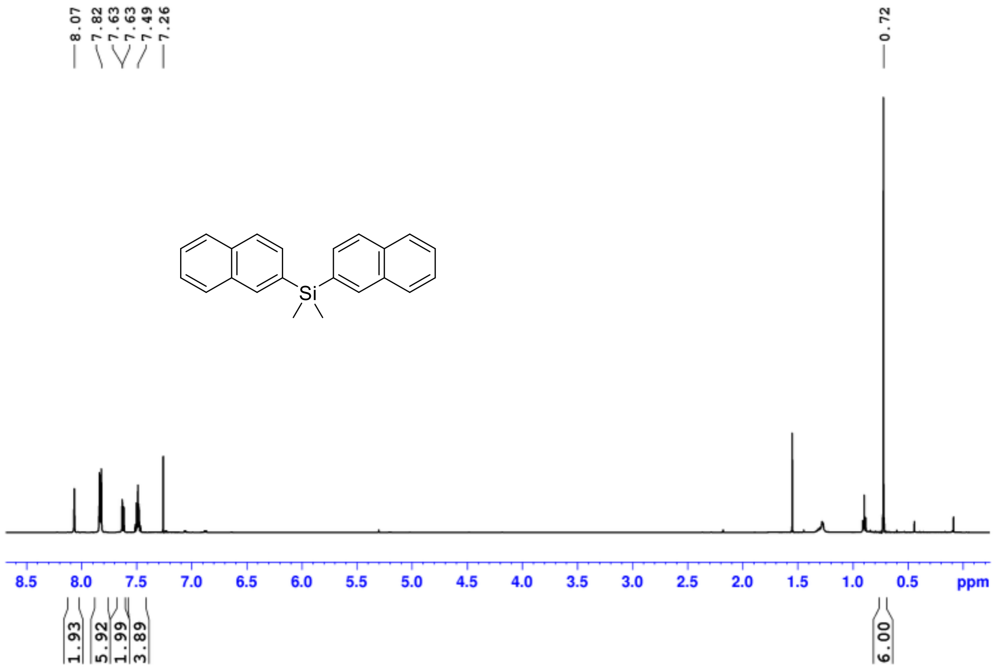

Figure S54. ^{1}H NMR spectrum of (naphthalen-2-yl)$_2$Me$_2$Si

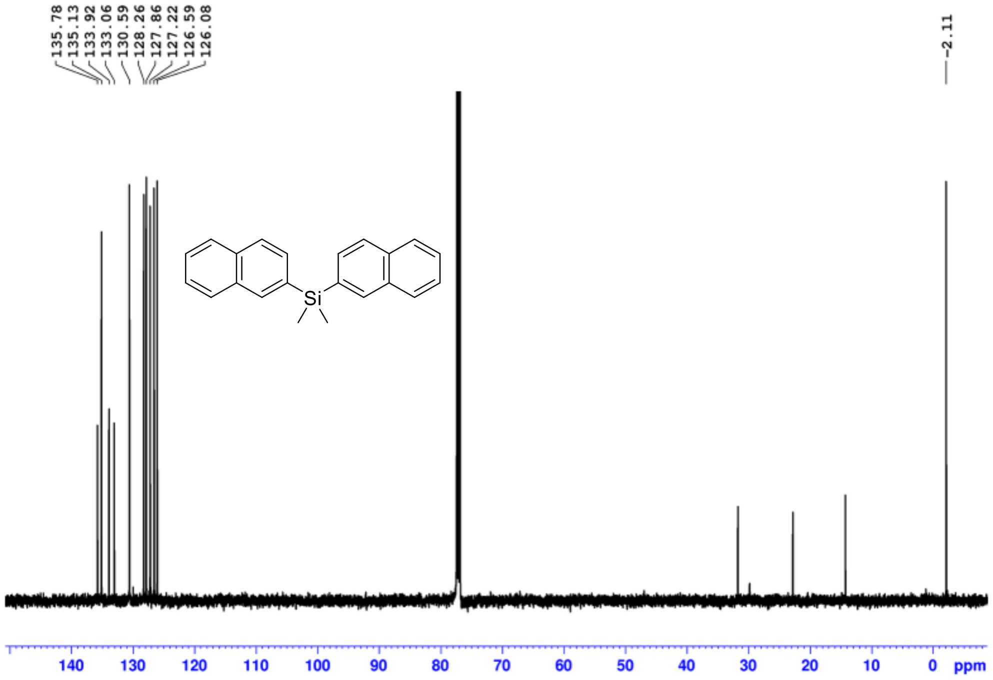

Figure S55. $^{13}C\{^1H\}$ **NMR spectrum of (naphthalen-2-yl)$_2$Me$_2$Si**

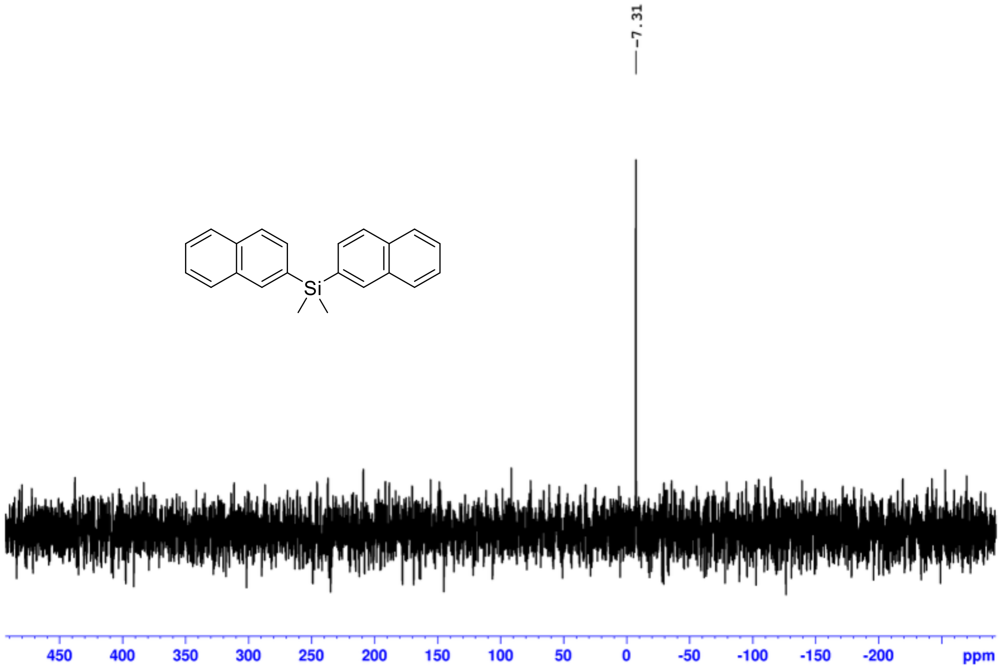

Figure S56. $^{29}Si\{^1H\}$ **NMR spectrum of (naphthalen-2-yl)$_2$Me$_2$Si**

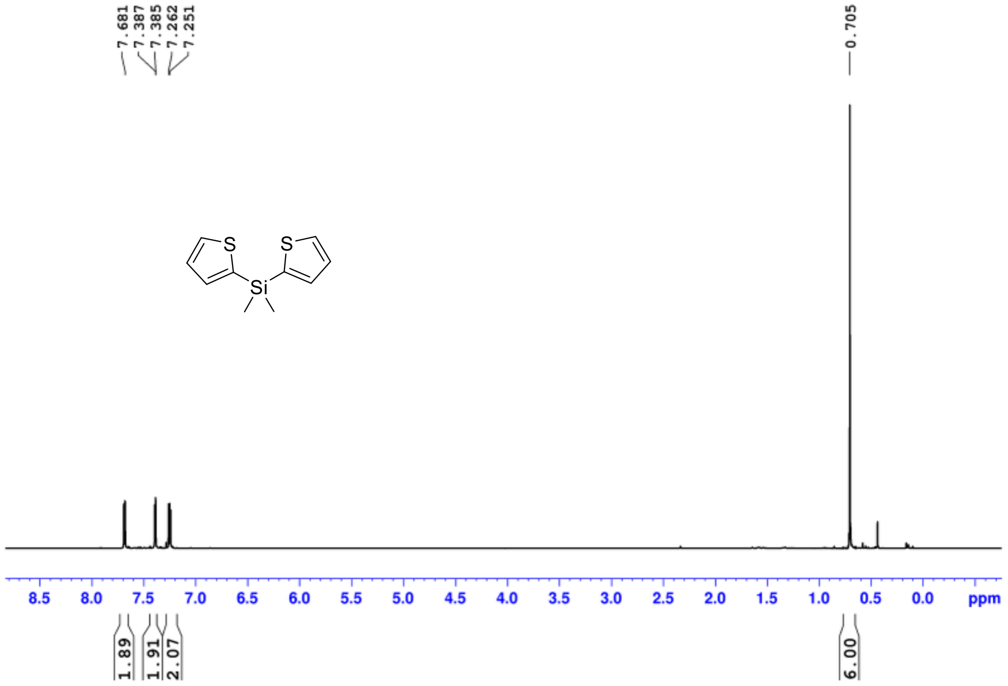

Figure S57. ^{1}**H NMR spectrum of (thiophen-2-yl)$_2$Me$_2$Si**

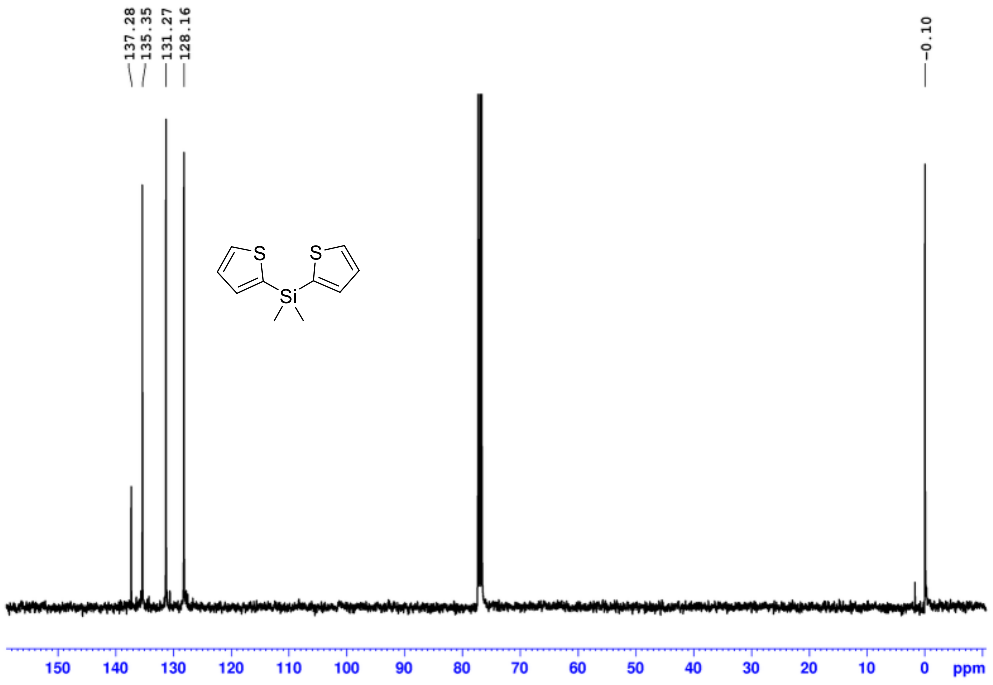

Figure S58. ^{13}**C{^{1}H} NMR spectrum of (thiophen-2-yl)$_2$Me$_2$Si**

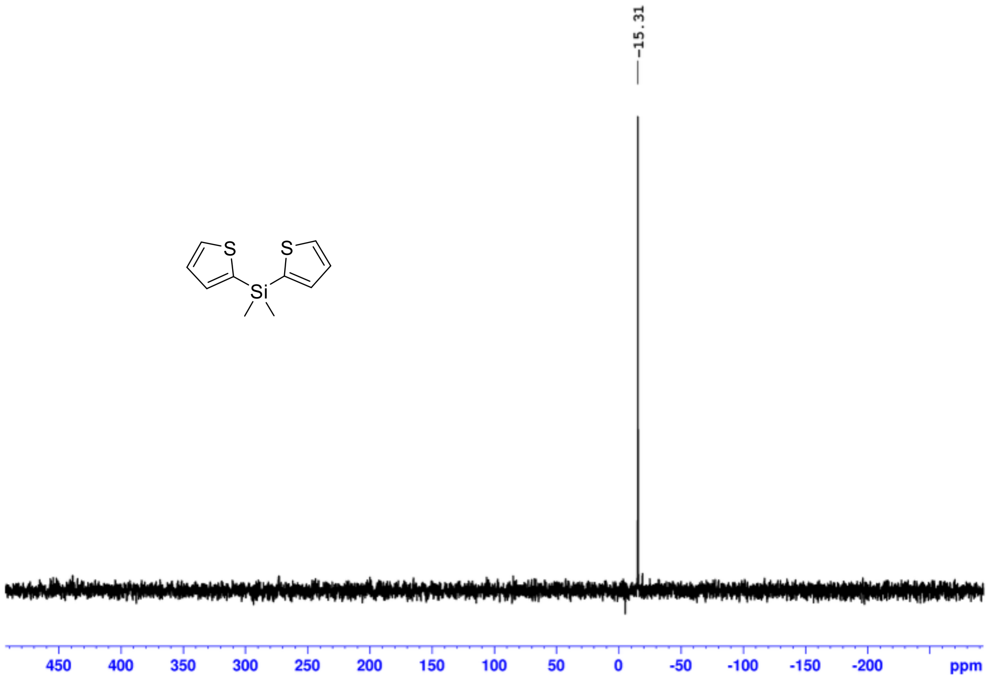

Figure S59. $^{29}Si\{^{1}H\}$ NMR spectrum of (thiophen-2-yl)$_2$Me$_2$Si

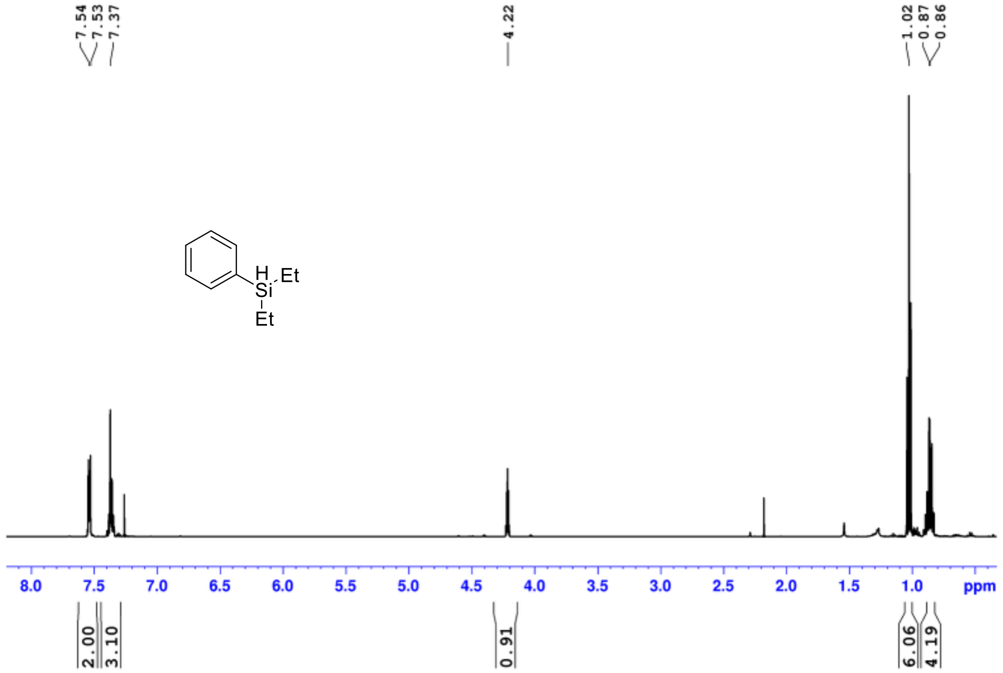

Figure S60. ^{1}H NMR spectrum of PhEt$_2$SiH

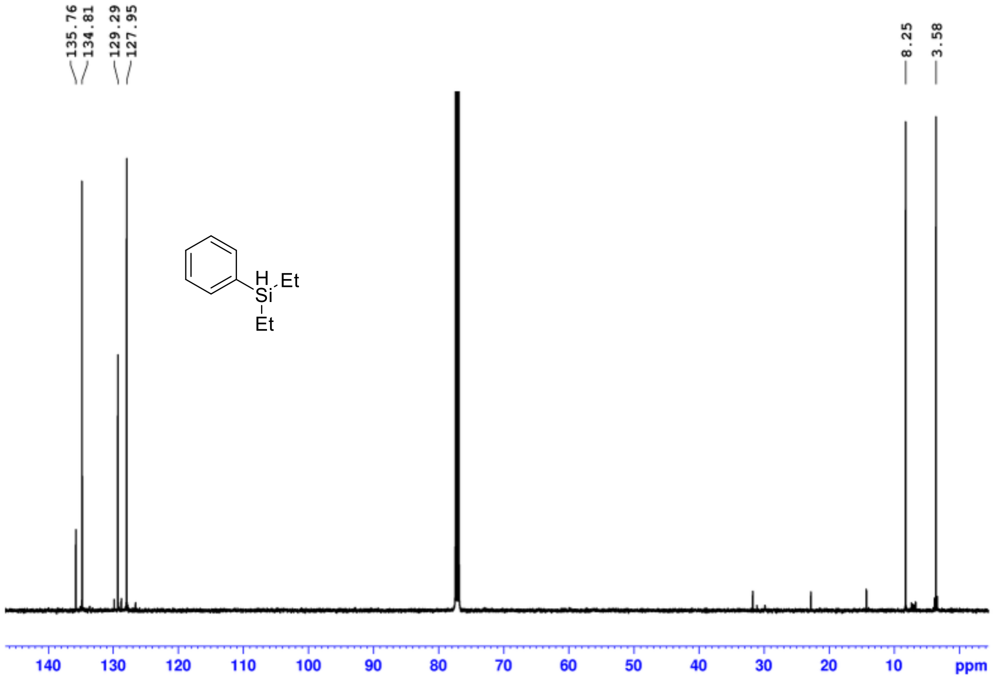

Figure S61. $^{13}C\{^1H\}$ **NMR spectrum of** $PhEt_2SiH$

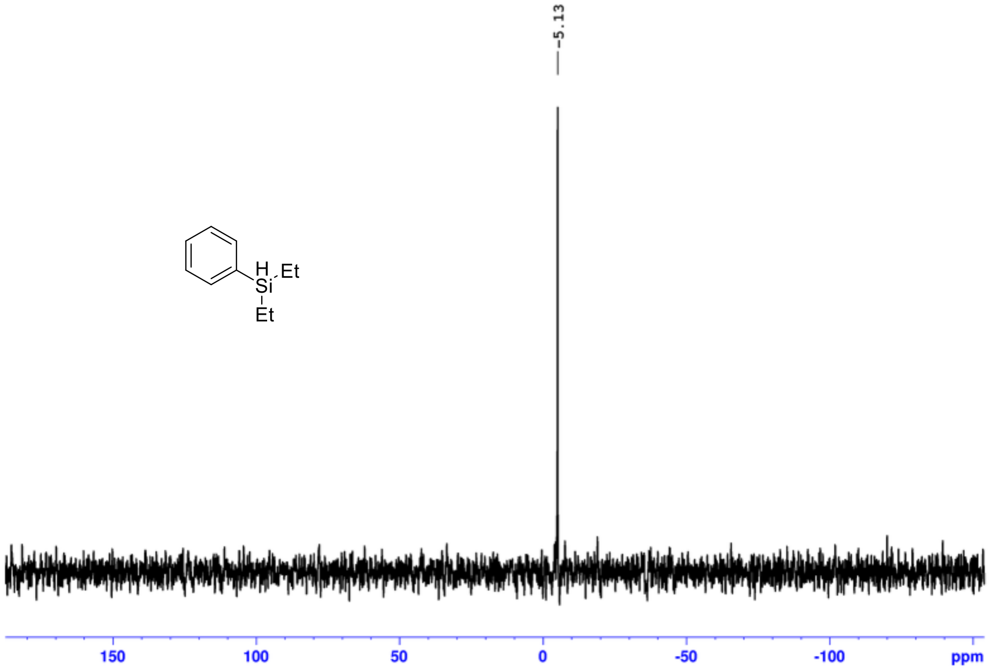

Figure S62. $^{29}Si\{^1H\}$ **NMR spectrum of** $PhEt_2SiH$

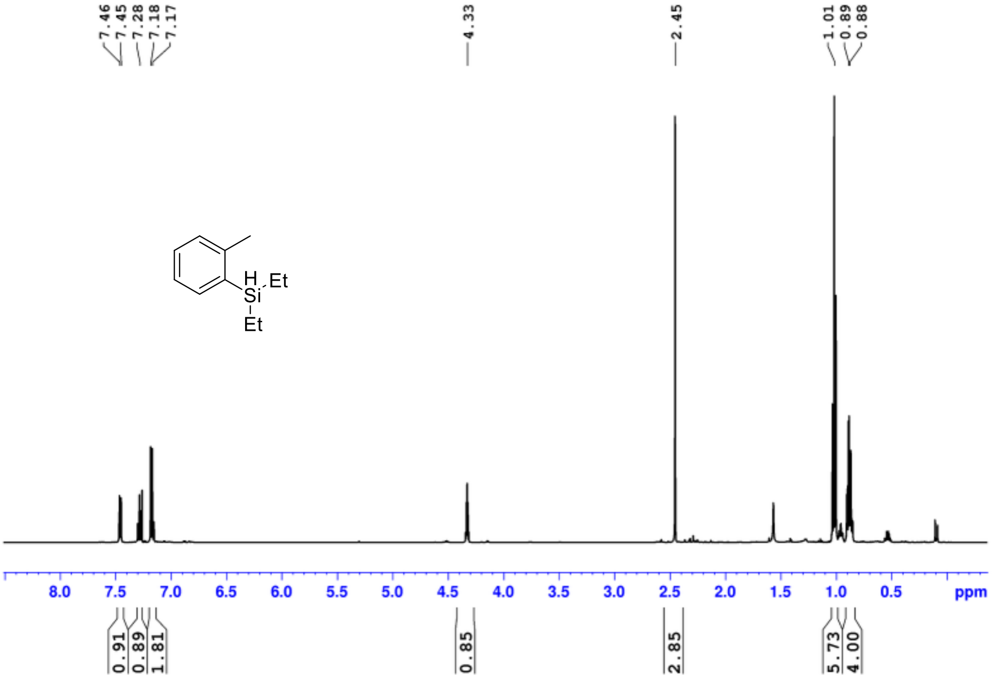

Figure S63. ^{1}H NMR spectrum of (2-tolyl)Et_2SiH

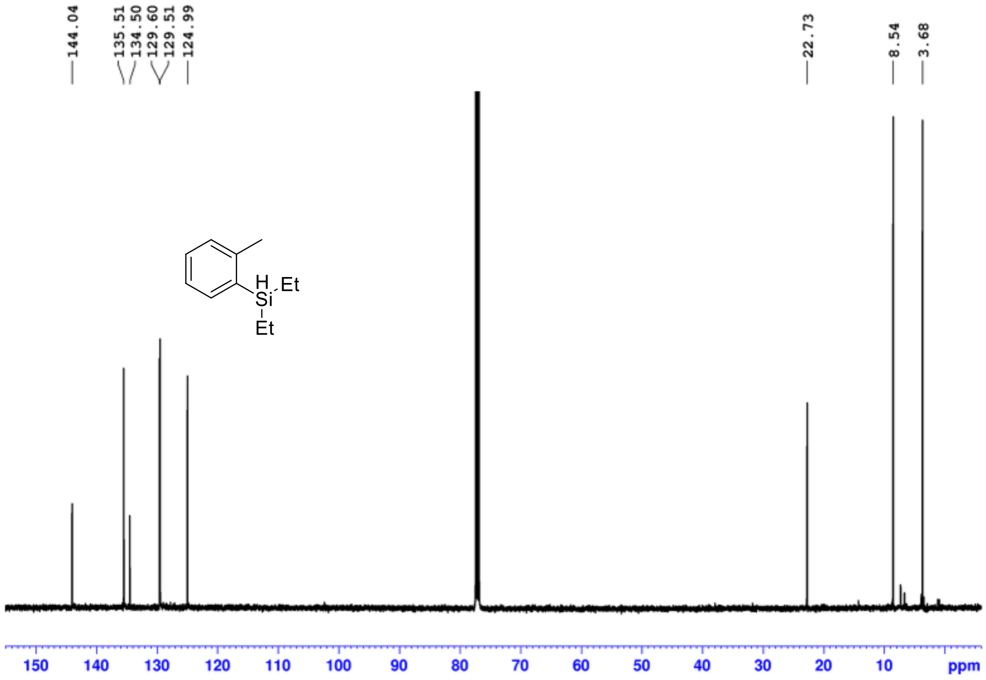

Figure S64. $^{13}C\{^1H\}$ NMR spectrum of (2-tolyl)Et_2SiH

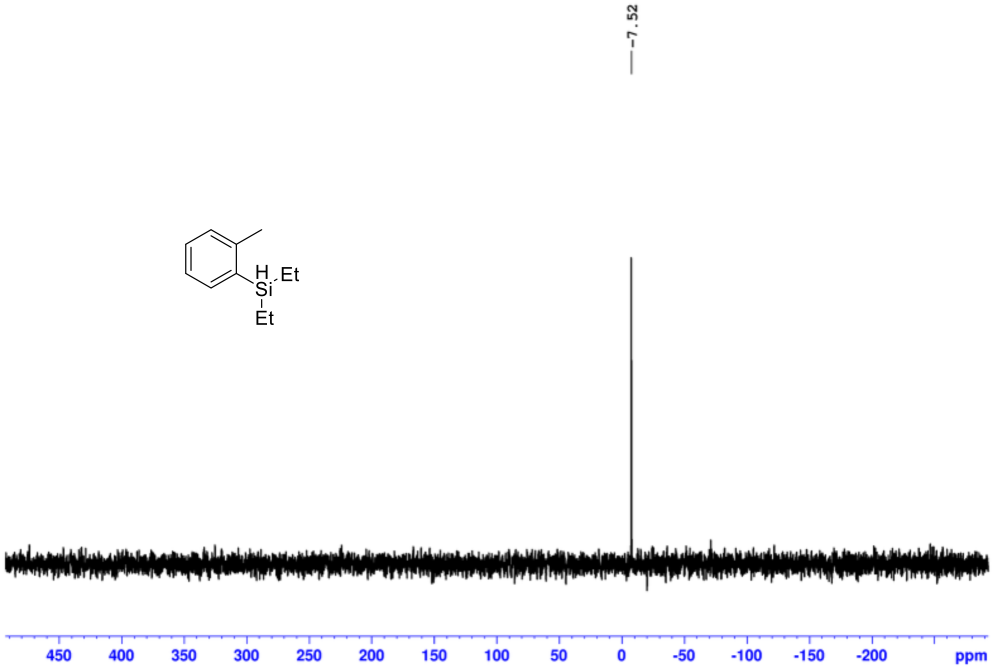

Figure S65. $^{29}Si\{^1H\}$ NMR spectrum of (2-tolyl)Et_2SiH

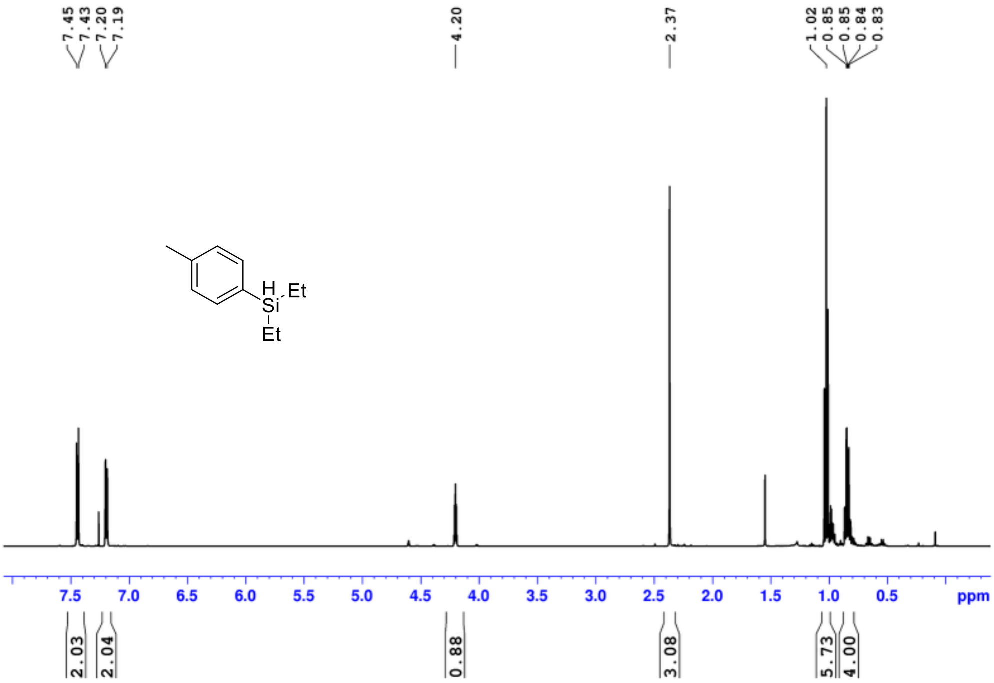

Figure S66. ^1H NMR spectrum of (4-tolyl)Et_2SiH

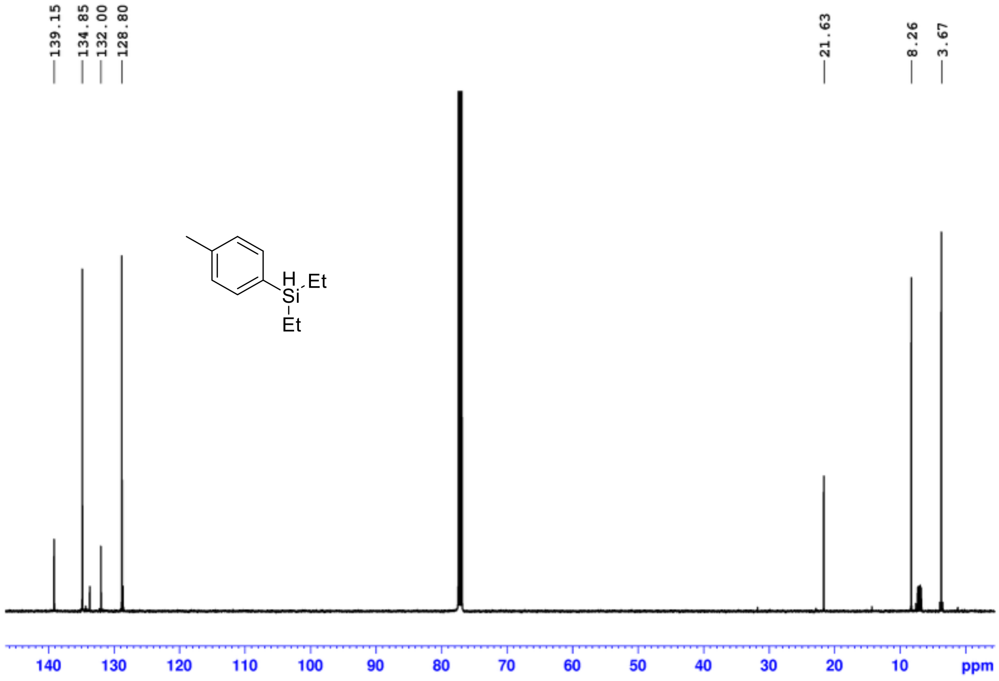

Figure S67. $^{13}C\{^1H\}$ **NMR spectrum of (4-tolyl)Et_2SiH**

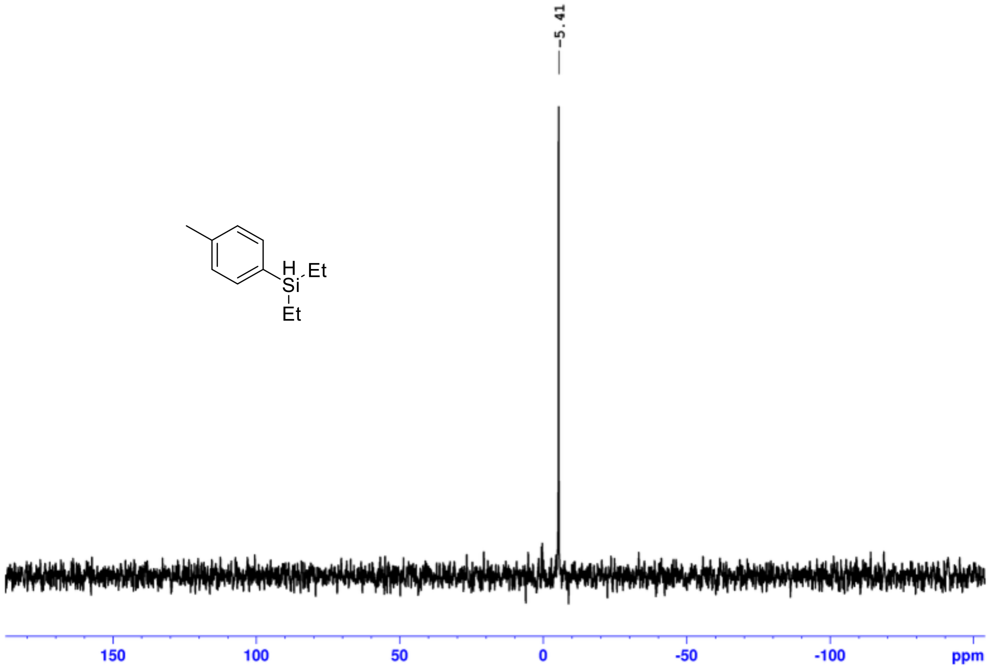

Figure S68. $^{29}Si\{^1H\}$ **NMR spectrum of (4-tolyl)Et_2SiH**

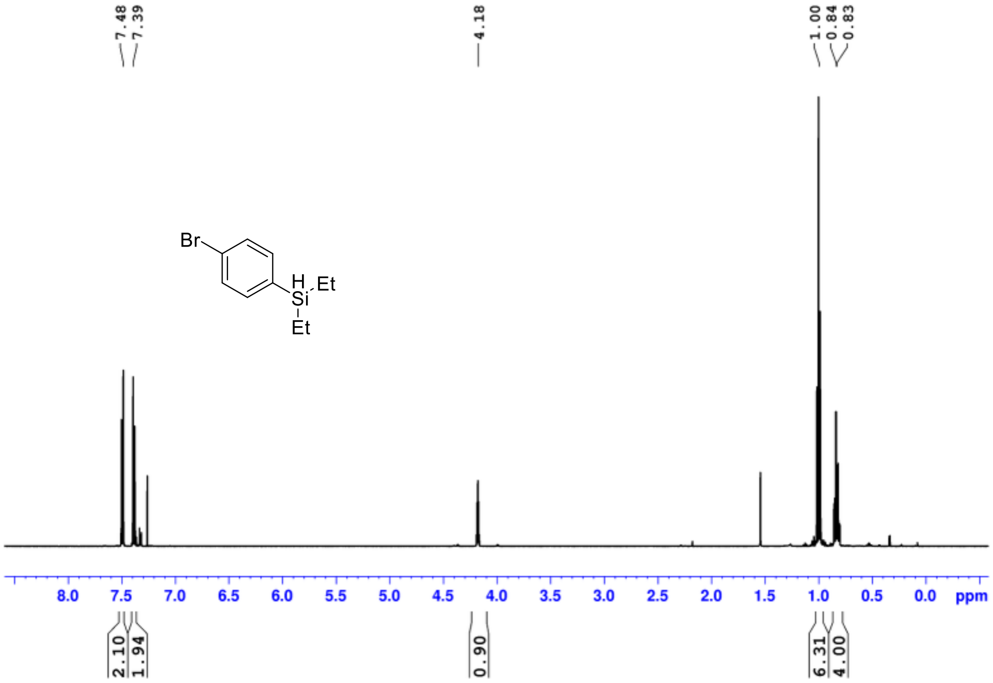

Figure S69. ^{1}H NMR spectrum of (4-Br-C_6H_4)Et_2SiH

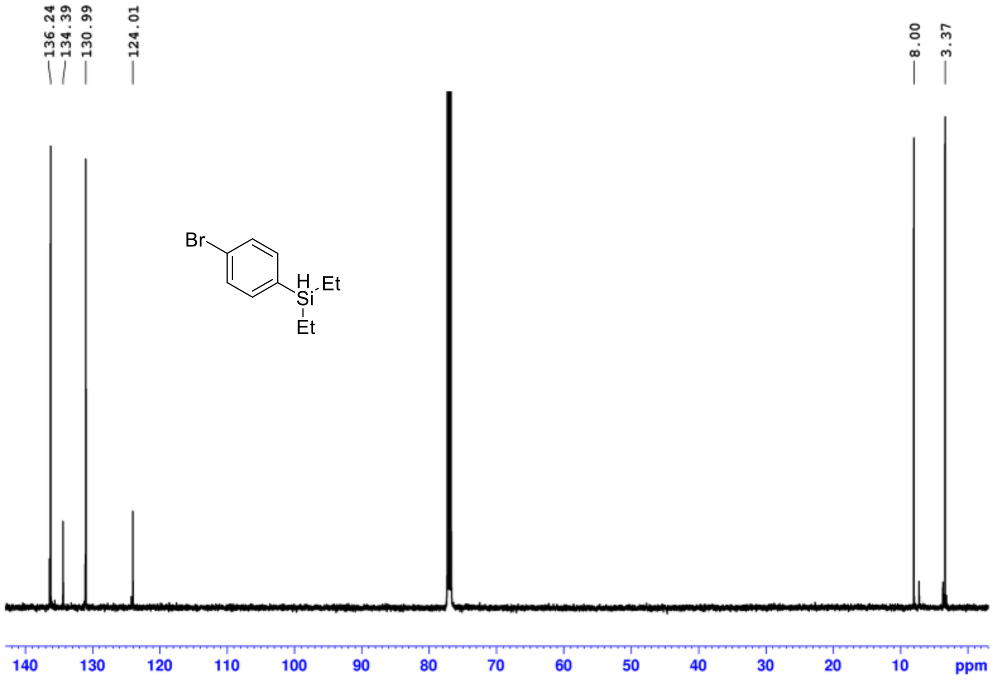

Figure S70. ^{13}C{^{1}H} NMR spectrum of (4-Br-C_6H_4)Et_2SiH

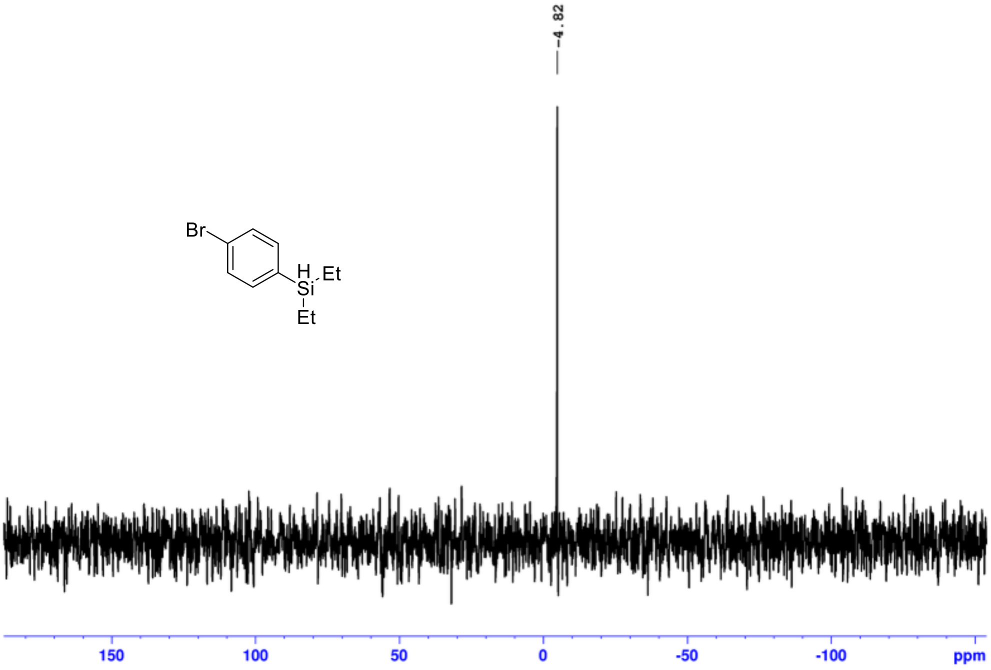

Figure S71. $^{29}Si\{^{1}H\}$ **NMR spectrum of (4-Br-C_6H_4)Et_2SiH**

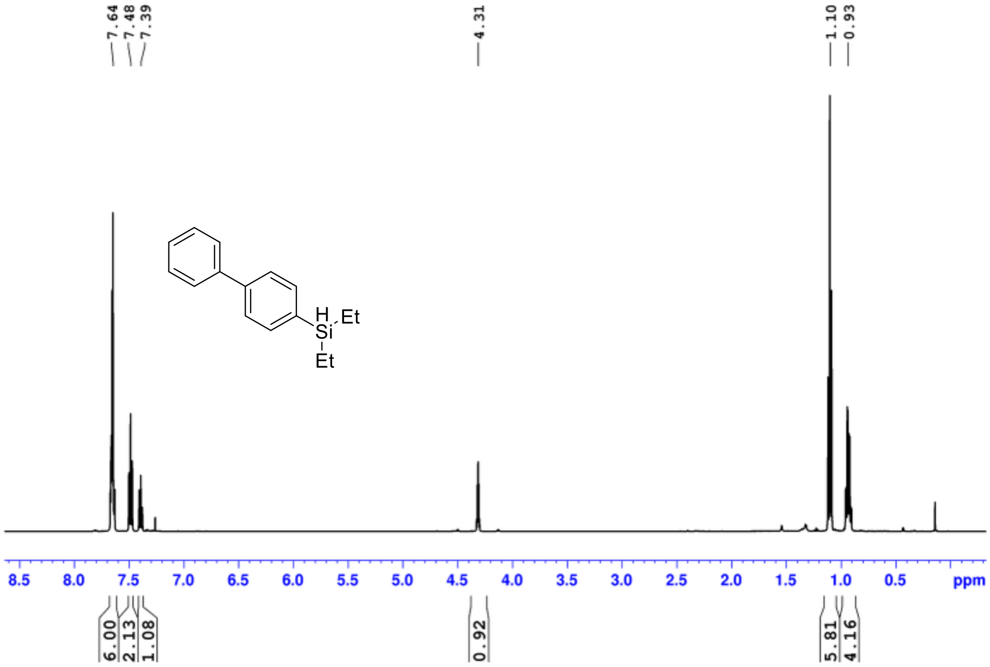

Figure S72. ^{1}H **NMR spectrum of (biphenyl-4-yl)Et_2SiH**

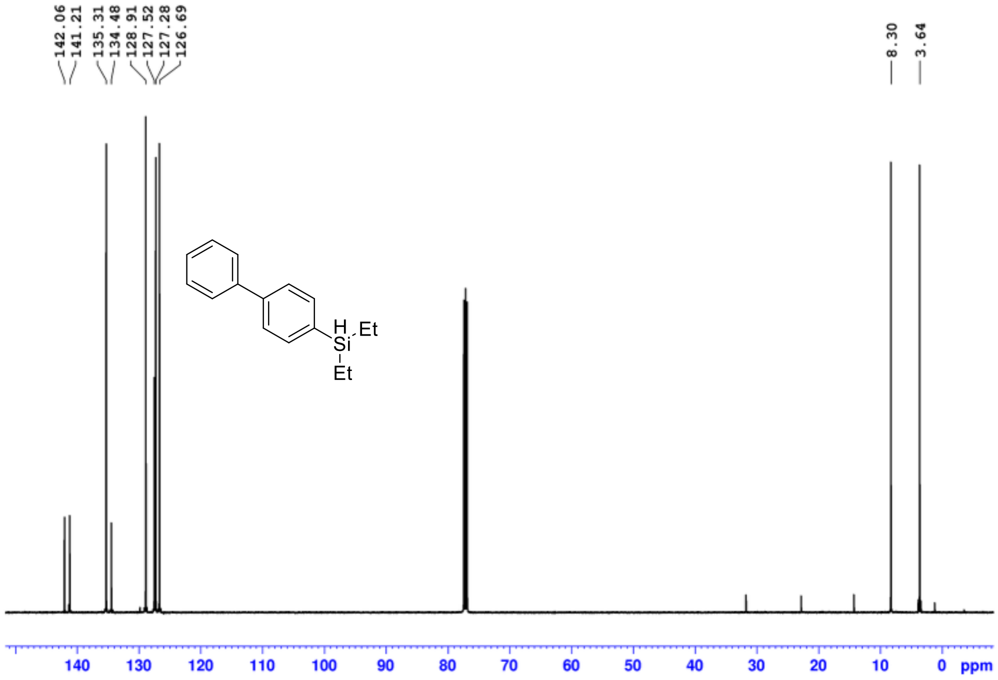

Figure S73. $^{13}C\{^{1}H\}$ NMR spectrum of (biphenyl-4-yl)Et_2SiH

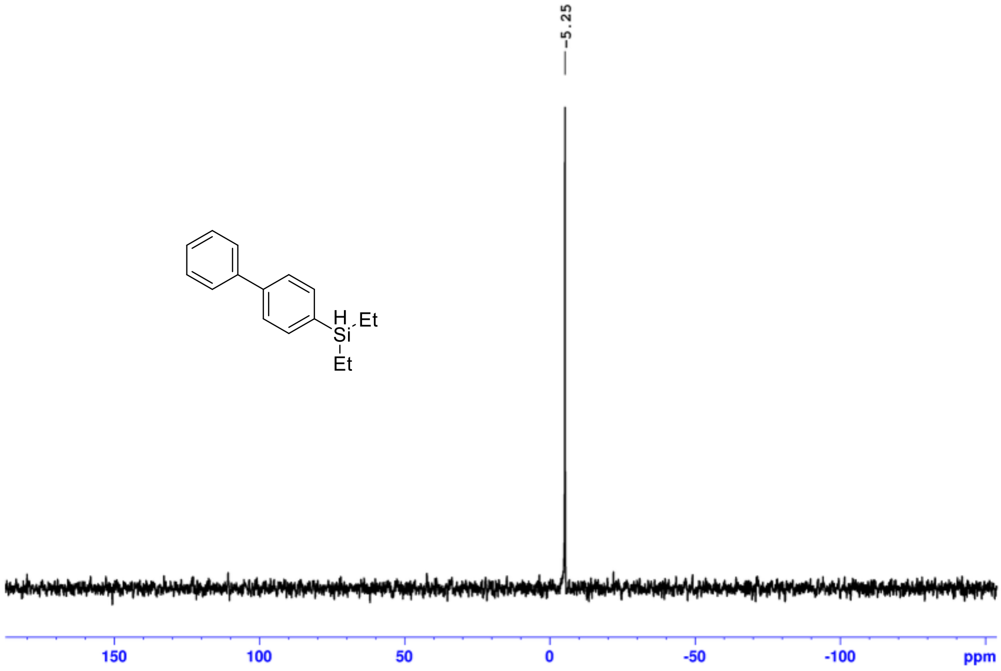

Figure S74. ^{29}Si NMR spectrum of (biphenyl-4-yl)Et_2SiH

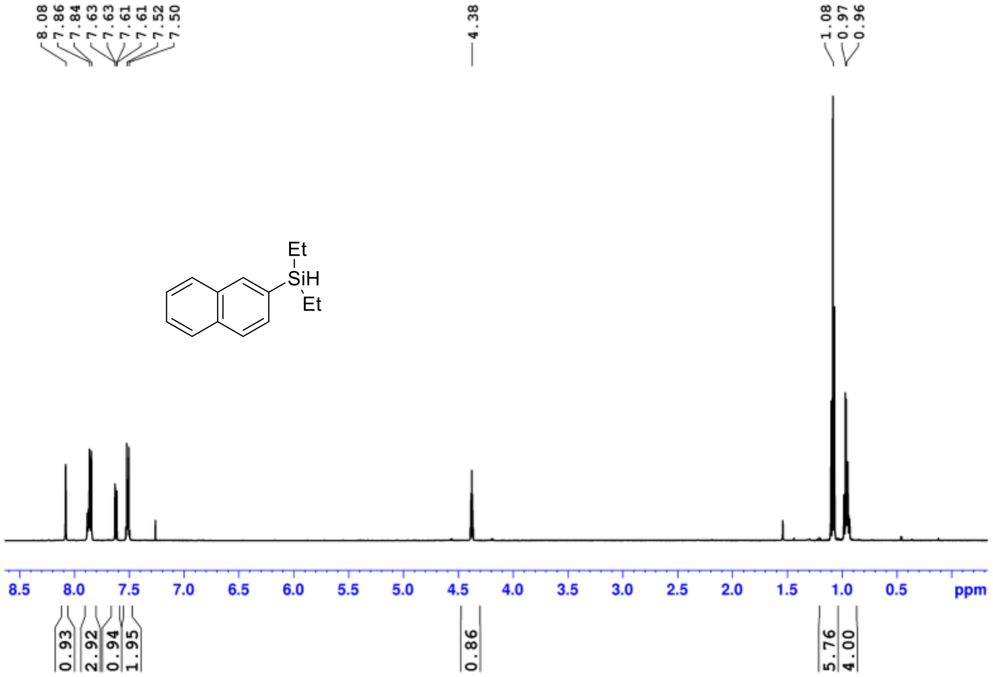

Figure S75. ^{1}H **NMR spectrum of (naphthalen-2-yl)Et$_2$SiH**

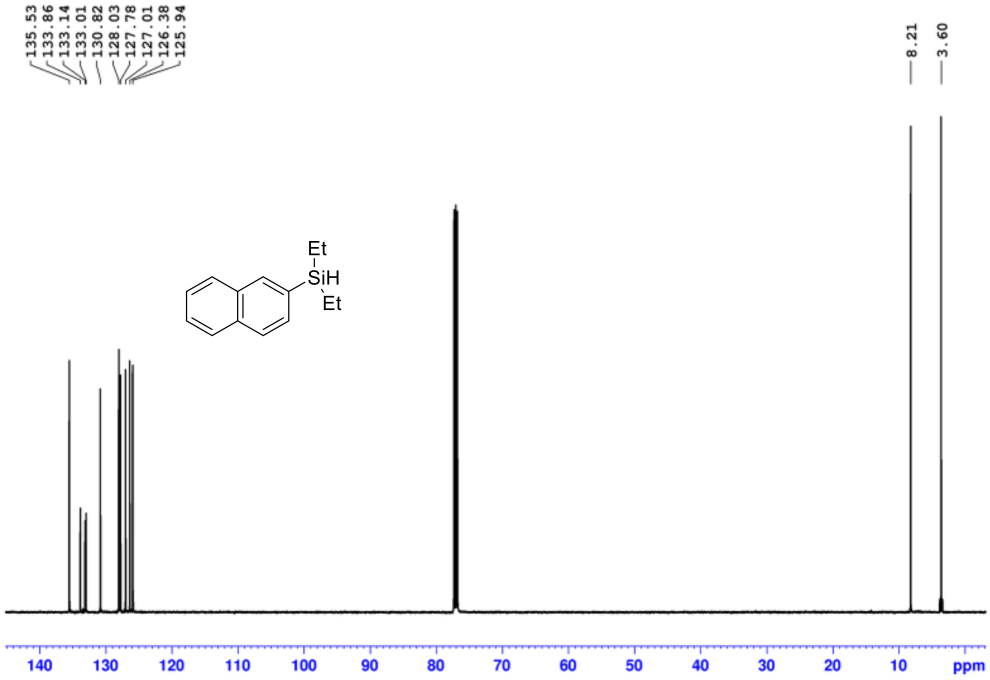

Figure S76. $^{13}C\{^{1}H\}$ **NMR spectrum of (naphthalen-2-yl)Et$_2$SiH**

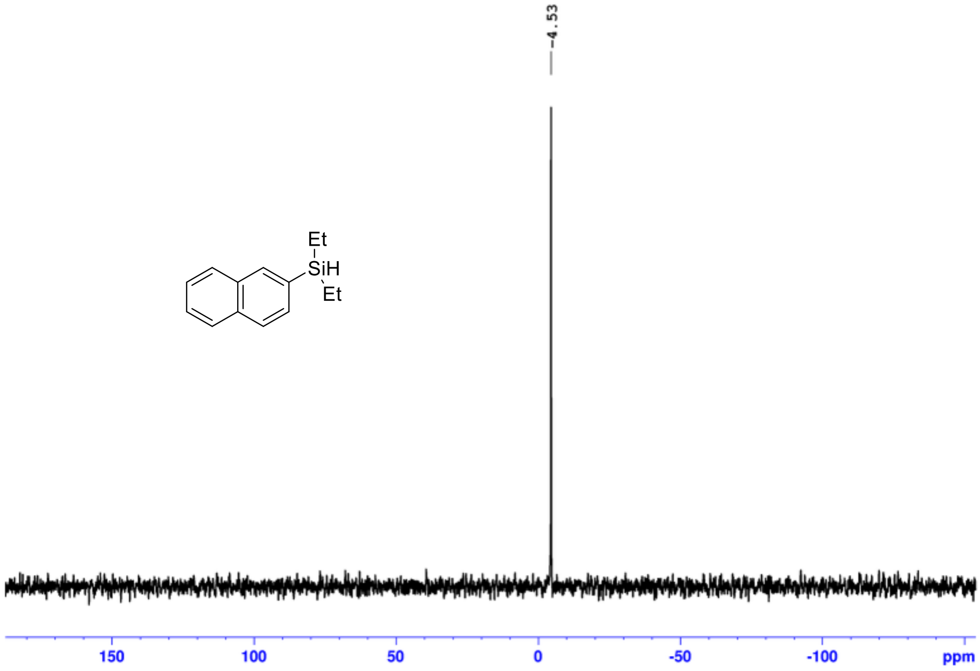

Figure S77. $^{29}Si\{^{1}H\}$ NMR spectrum of (naphthalen-2-yl)Et_2SiH

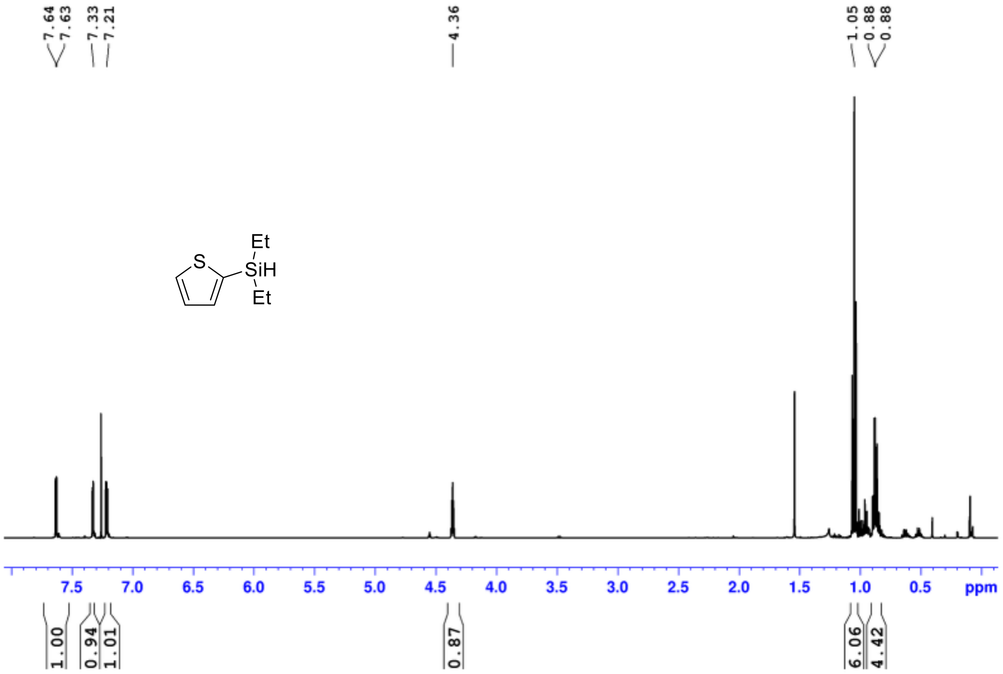

Figure S78. ^{1}H NMR spectrum of (thiophen-2-yl)Et_2SiH

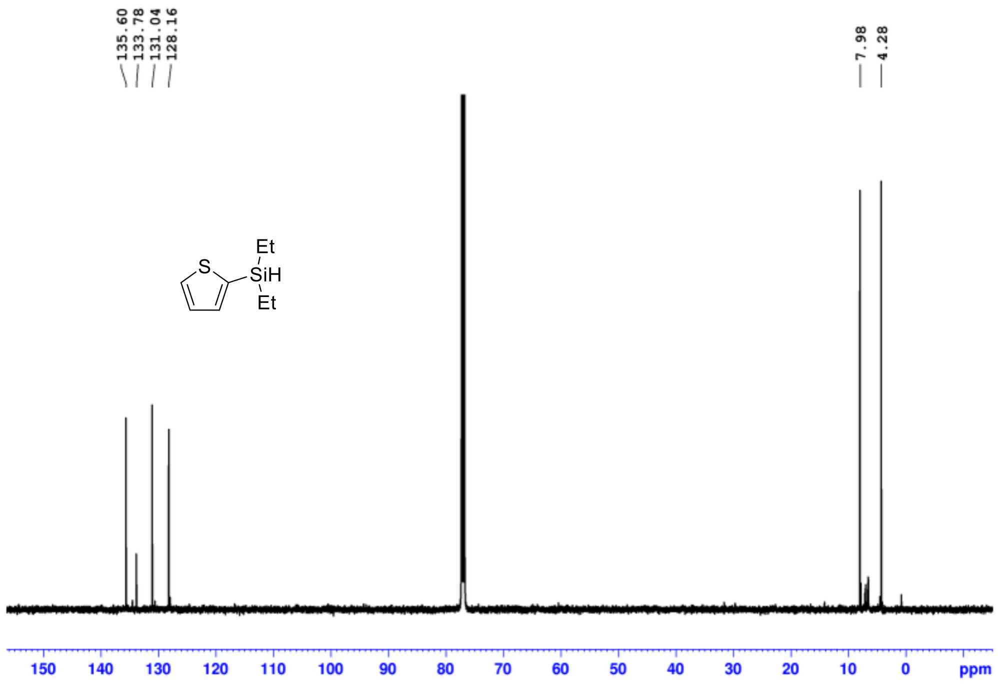

Figure S79. $^{13}C\{^{1}H\}$ **NMR spectrum of (thiophen-2-yl)Et_2SiH**

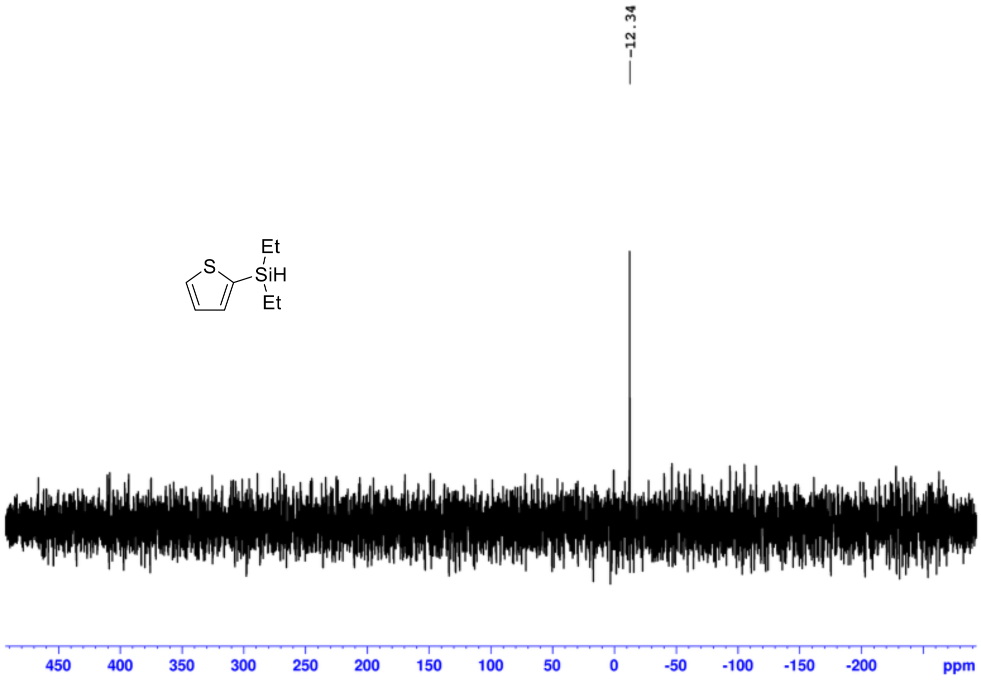

Figure S80. $^{29}Si\{^{1}H\}$ **NMR spectrum of (thiophen-2-yl)Et_2SiH**

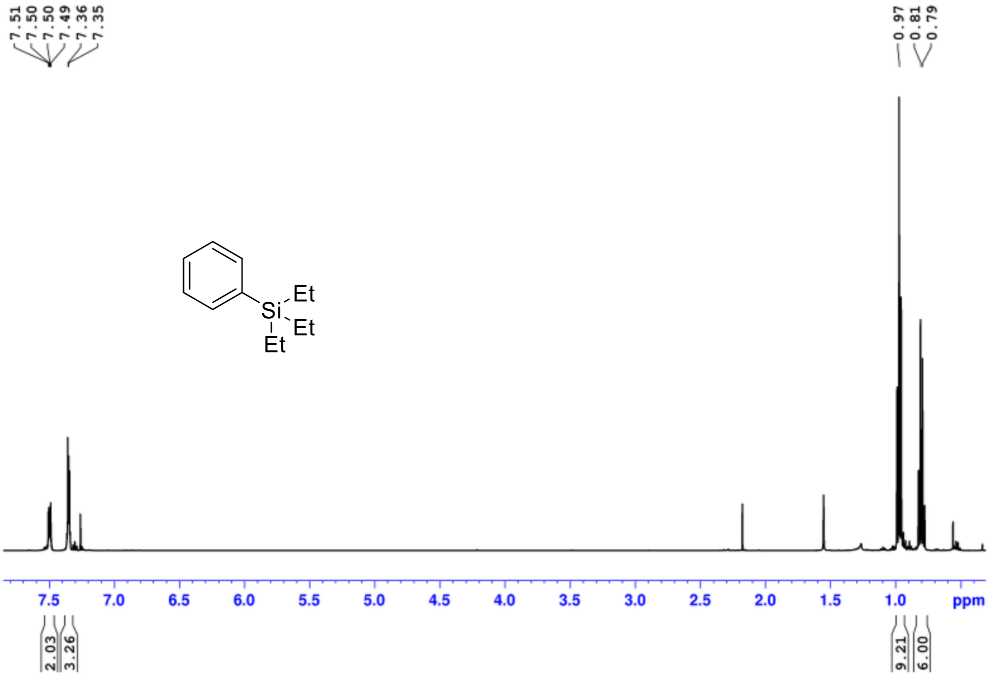

Figure S81. ^{1}H NMR spectrum of $PhEt_3Si$

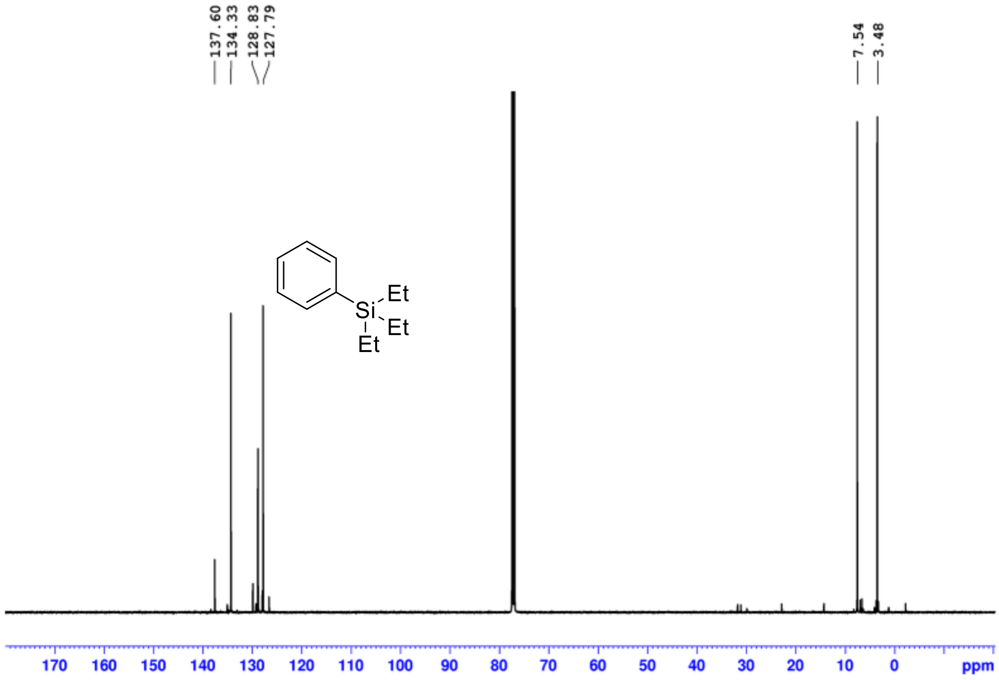

Figure S82. $^{13}C\{^{1}H\}$ NMR spectrum of $PhEt_3Si$

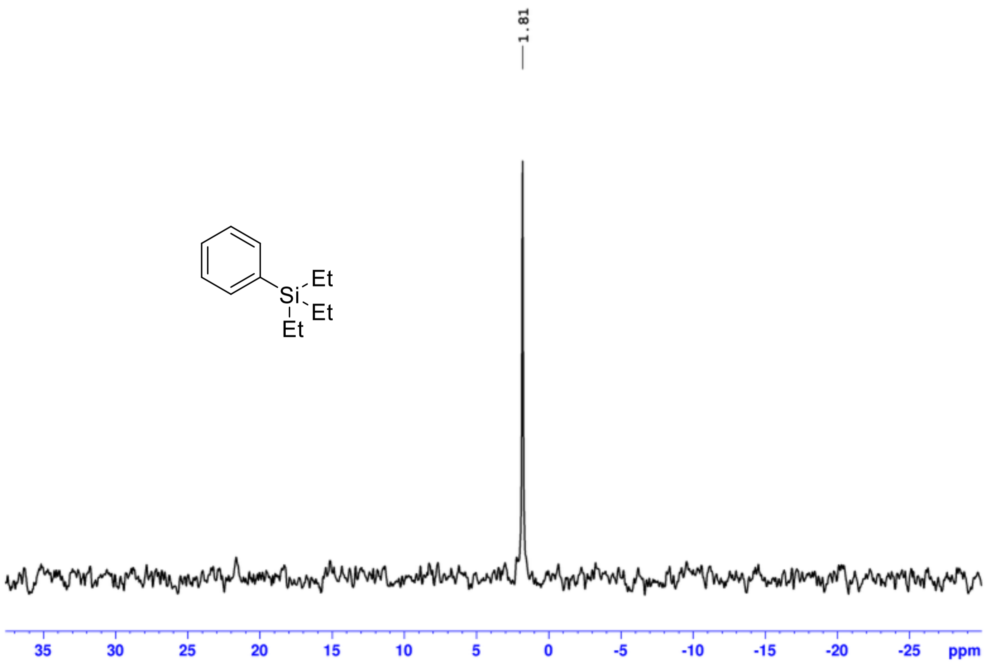

Figure S83. $^{29}Si\{^{1}H\}$ NMR spectrum of $PhEt_3Si$

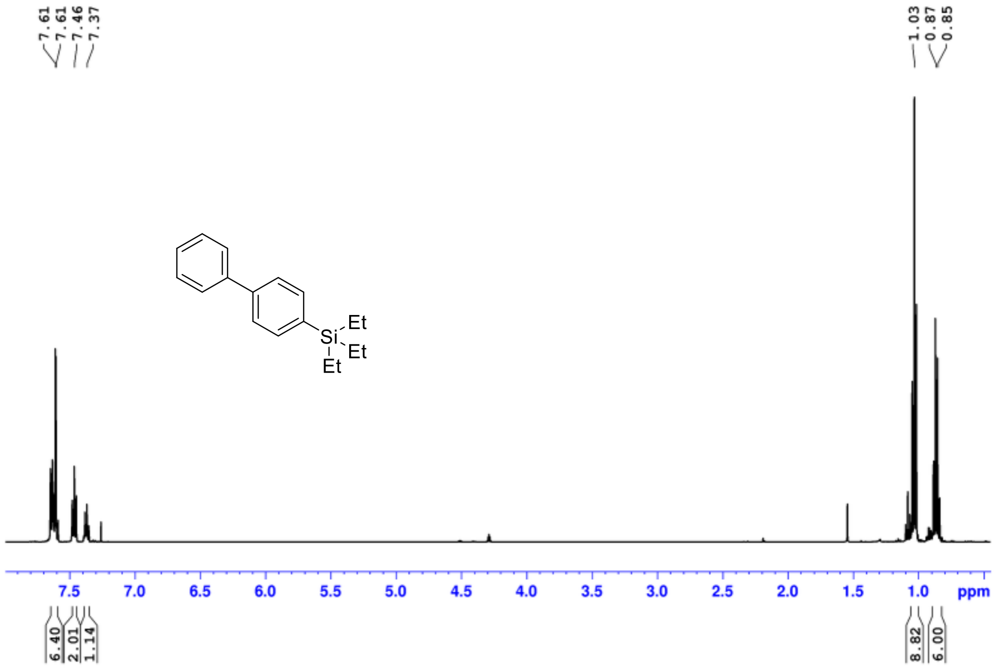

Figure S84. ^{1}H NMR spectrum of (biphenyl-4-yl)Et_3Si

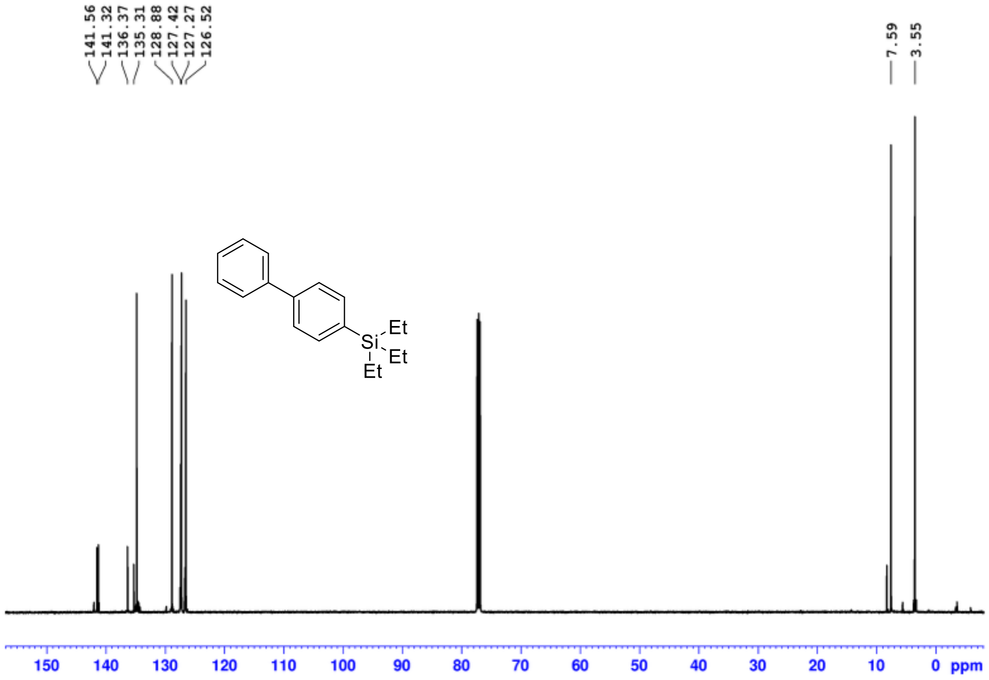

Figure S85. $^{13}C\{^1H\}$ NMR spectrum of (biphenyl-4-yl)Et_3Si

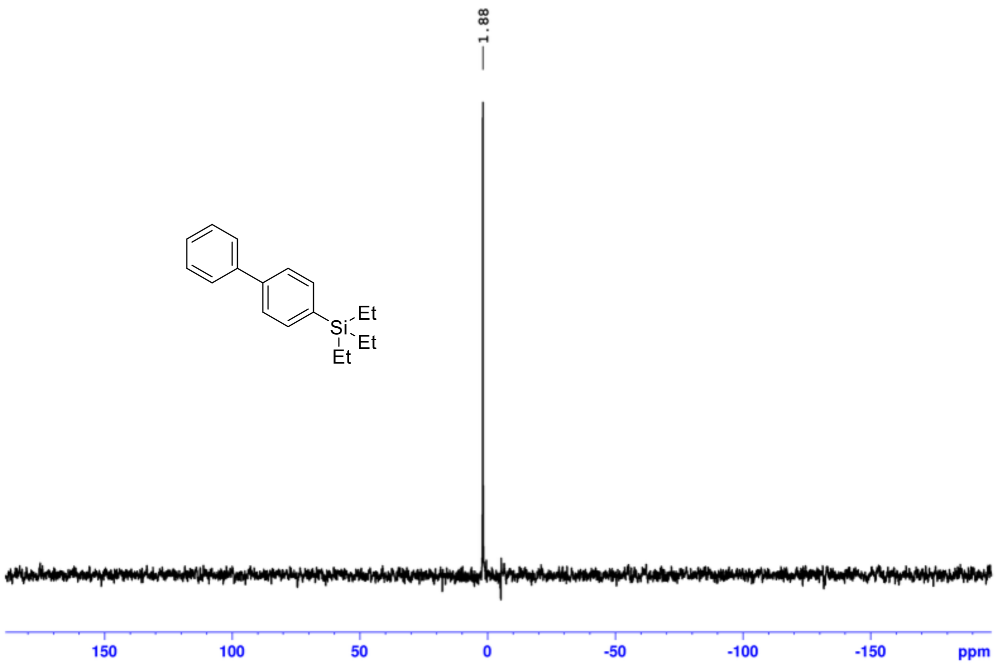

Figure S86. $^{29}Si\{^1H\}$ NMR spectrum of (biphenyl-4-yl)Et_3Si

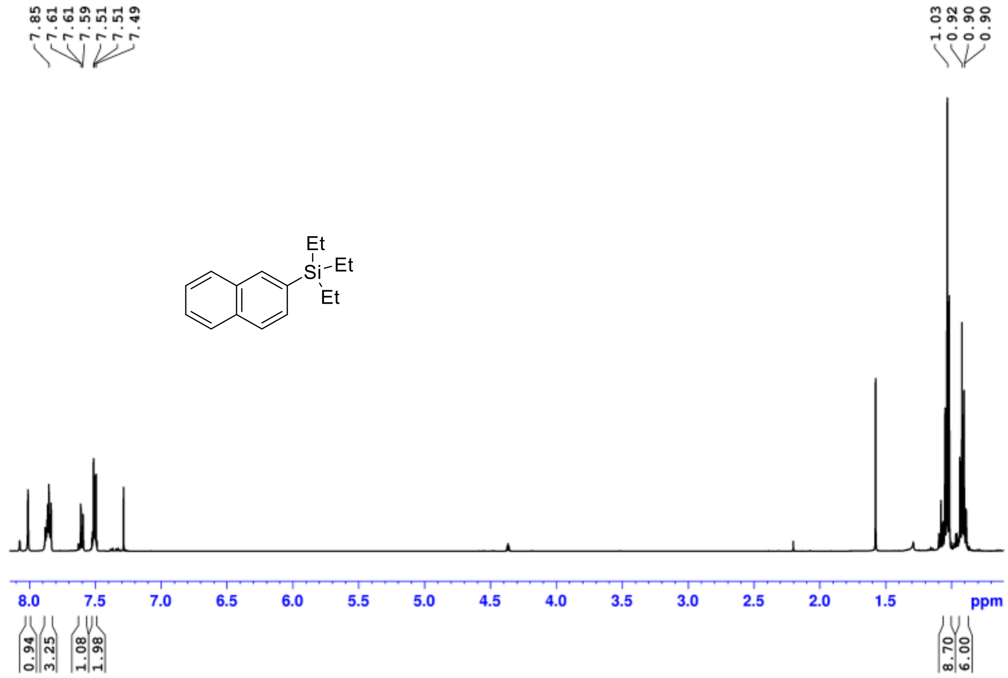

Figure S87. **^{1}H NMR spectrum of (naphthalen-2-yl)Et$_3$Si**

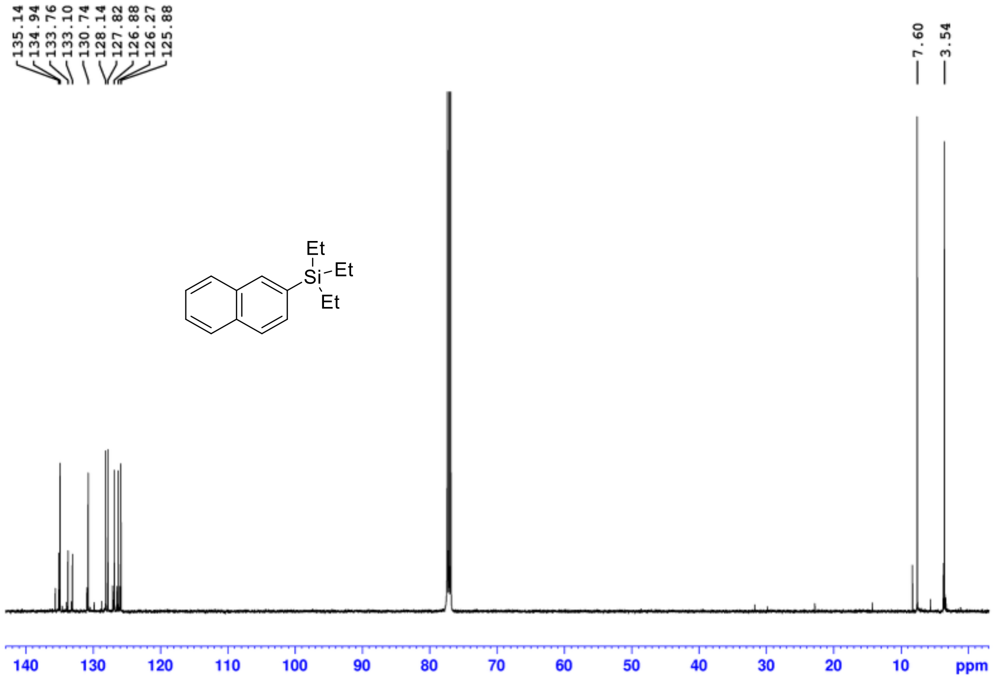

Figure S88. **^{13}C{^{1}H} NMR spectrum of (naphthalen-2-yl)Et$_3$Si**

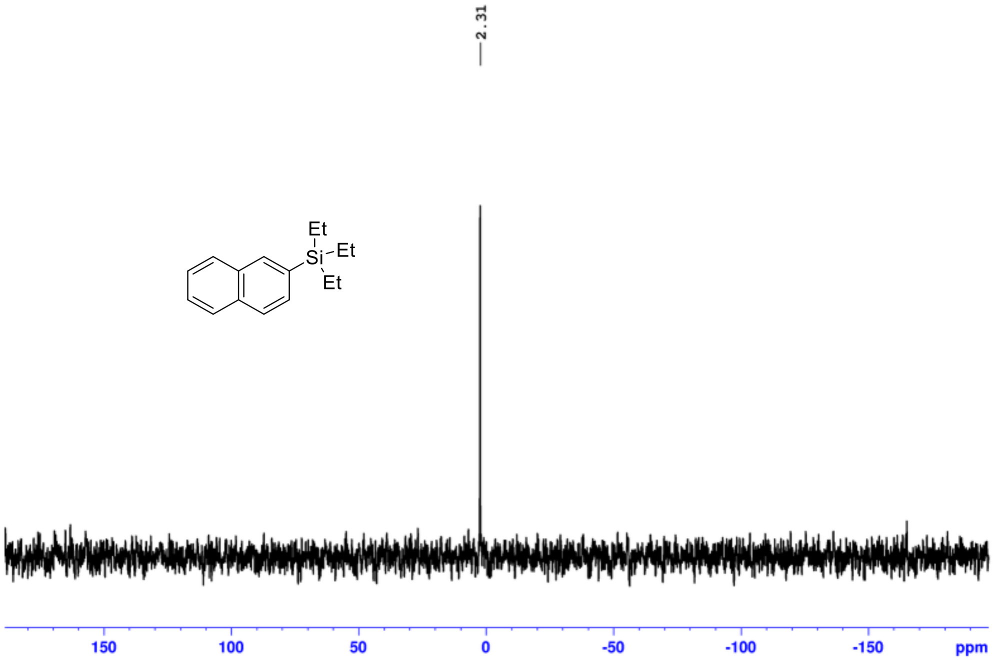

Figure S89. $^{29}Si\{^1H\}$ NMR spectrum of (naphthalen-2-yl)Et_3Si

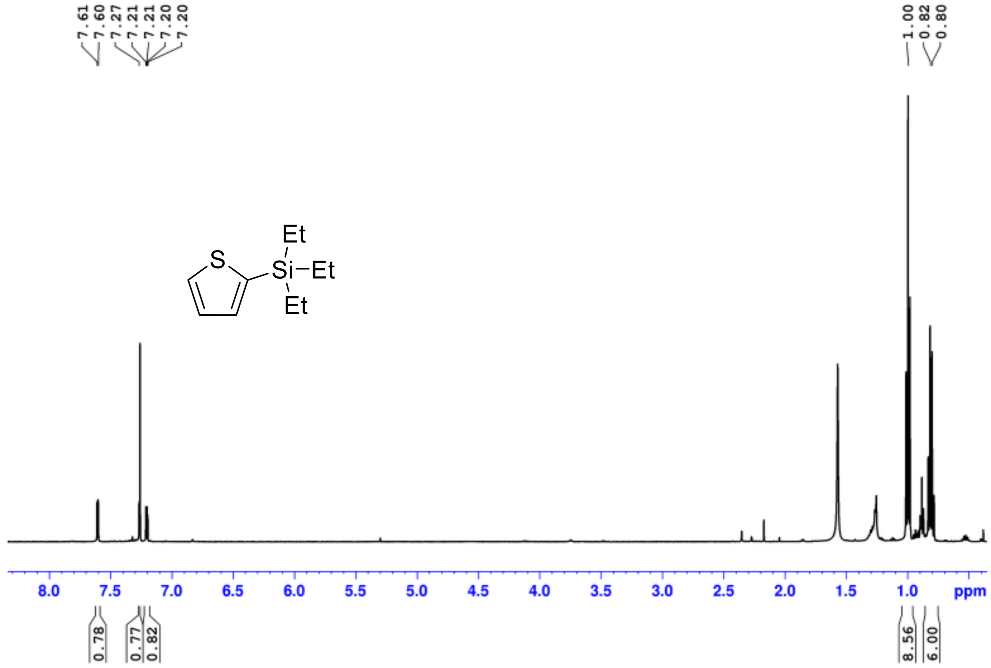

Figure S90. ^1H NMR spectrum of (thiophen-2-yl)Et_3Si

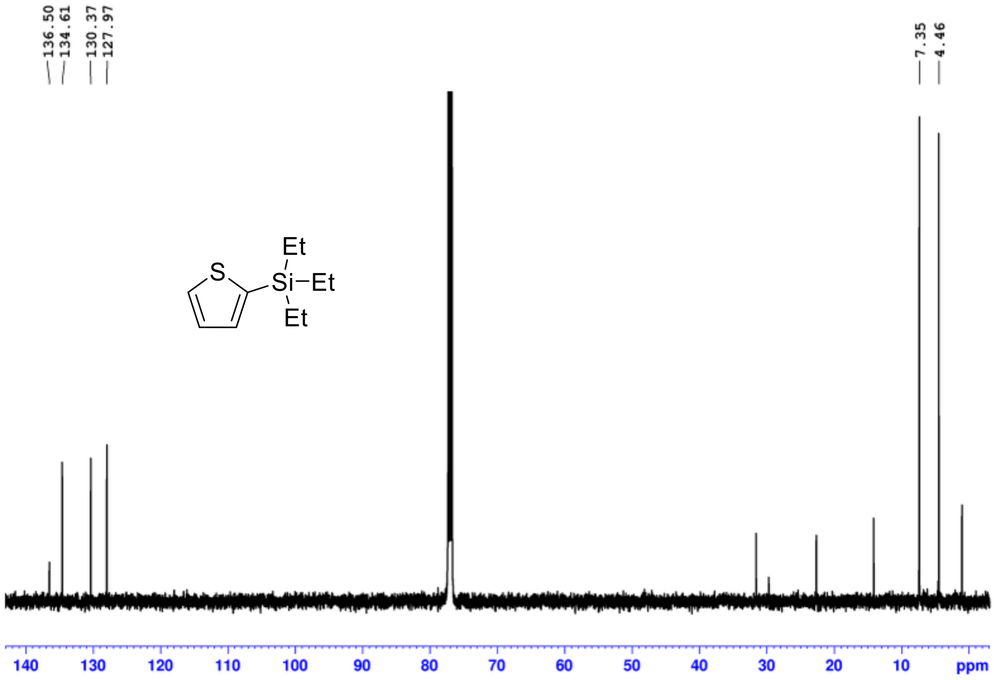

Figure S91. $^{13}C\{^{1}H\}$ NMR spectrum of (thiophen-2-yl)Et_3Si

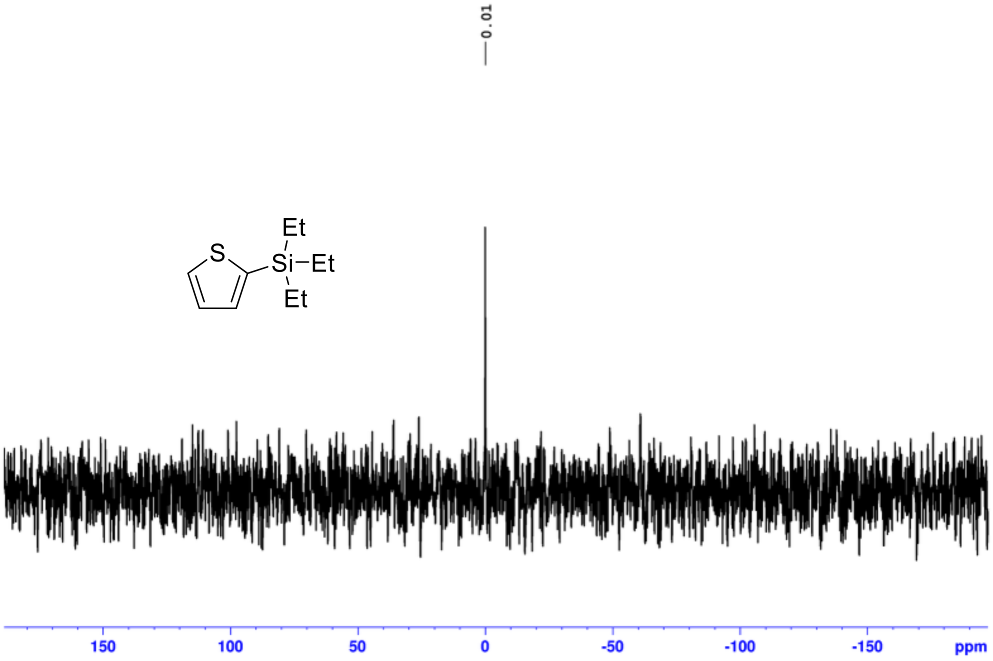

Figure S92. $^{29}Si\{^{1}H\}$ NMR spectrum of (thiophen-2-yl)Et_3Si

6. References

(1) Enthaler, S. Zinc-Catalyzed Depolymerization of End-of-Life Polysiloxanes. *Angewandte Chemie International Edition* **2014**, *53* (10), 2716-2721. DOI: https://doi.org/10.1002/anie.201309299.

(2) Ma, Y.; Zhang, L.; Luo, Y.; Nishiura, M.; Hou, Z. B(C6F5)3-Catalyzed C–Si/Si–H Cross-Metathesis of Hydrosilanes. *Journal of the American Chemical Society* **2017**, *139* (36), 12434-12437. DOI: 10.1021/jacs.7b08053.

(3) Tour, J. M.; John, J. A.; Stephens, E. B. Convenient route to di- and triorganosilyl ethyl ethers and the corresponding di- and triorganosilanes. *Journal of Organometallic Chemistry* **1992**, *429* (3), 301-310. DOI: https://doi.org/10.1016/0022-328X(92)83181-G.

(4) Zhang, Q.; Peng, M.; Gao, Z.; Guo, W.; Sun, Z.; Zhao, Y.; Zhou, W.; Wang, M.; Mei, B.; Du, X.-L.; et al. Nitrogen-Neighbored Single-Cobalt Sites Enable Heterogeneous Oxidase-Type Catalysis. *Journal of the American Chemical Society* **2023**, *145* (7), 4166-4176. DOI: 10.1021/jacs.2c12586.

(5) Rayment, E. J.; Summerhill, N.; Anderson, E. A. Synthesis of Phenols via Fluoride-free Oxidation of Arylsilanes and Arylmethoxysilanes. *The Journal of Organic Chemistry* **2012**, *77* (16), 7052-7060. DOI: 10.1021/jo301363h.

(6) Nakatani, S.; Ito, Y.; Sakurai, S.; Kodama, T.; Tobisu, M. Nickel-Catalyzed Decarbonylation of Acylsilanes. *The Journal of Organic Chemistry* **2020**, *85* (11), 7588-7594. DOI: 10.1021/acs.joc.0c00772.

(7) Tandura, S. N. S., Yu. A.; Androsenko, S. I.; Masterov, E. E.; Alekseev, N. V.; Rakitskaya, V. I.; Rodin, O. G.; Traven, V. F. Conformation of dibenzochalcogenosilacins and -silanins from proton, carbon-13, and silicon-29 NMR spectra. *Zhurnal Obshchei Khimii* **1988**, *58* (2), 398-406.

(8) Tobita, H.; Shiozawa, R.; Ogino, H. Reaction of Silyl(carbonyl)iron Complexes with LiAlH4 Giving Methylsilanes: Reduction of a Carbonyl Ligand and Coupling with a Silyl Group. *Chemistry Letters* **2004**, *26* (8), 805-806. DOI: 10.1246/cl.1997.805 (acccessed 1/2/2026).

(9) Gupta, V.; Mandal, S. K. A Highly Stable Triazole-Functionalized Metal–Organic Framework Integrated with Exposed Metal Sites for Selective CO2 Capture and Conversion. *Chemistry – A European Journal* **2020**, *26* (12), 2658-2665. DOI: https://doi.org/10.1002/chem.201903912.

(10) Correa-Duran, F.; Allred, A. L.; Glover, D. E.; Smith, D. E. Dimethylsilyl- and methylene-bridged aromatic groups: I. Polarography and ultraviolet spectroscopy. *Journal of Organometallic Chemistry* **1973**, *49* (2), 353-364. DOI: https://doi.org/10.1016/S0022-328X(00)84225-4.

(11) Moreau, C.; Serein-Spirau, F.; Bordeau, M.; Biran, C.; Dunoguès, J. Electrochemical synthesis of bis(2-thienyl) silanes, 2-thienylchlorosilanes, bis[5-(2-bromothienyl)]silanes, and 5-(2-bromothienyl) dimethylchlorosilane, precursors of poly[(silanylene)thiophene]s. *Journal of Organometallic Chemistry* **1996**, *522* (2), 213-221. DOI: https://doi.org/10.1016/0022-328X(96)06333-4.

(12) Murakami, K.; Hirano, K.; Yorimitsu, H.; Oshima, K. Silver-Catalyzed Transmetalation between Chlorosilanes and Aryl and Alkenyl Grignard Reagents for the Synthesis of Tetraorganosilanes. *Angewandte Chemie International Edition* **2008**, *47* (31), 5833-5835. DOI: https://doi.org/10.1002/anie.200801949.

(13) Humbert, M.; Clerc, A.; Miqueu, K.; Monot, J.; Martin-Vaca, B.; Bourissou, D. Hydrogermylation of alkynes via metal–ligand cooperative catalysis. *Chemical Communications* **2025**, *61* (16), 3327-3330, 10.1039/D4CC06374K. DOI: 10.1039/D4CC06374K.

(14) Hu, S.-S.; Weber, W. P. Photolysis of 1,1,2,2-tetramethyl-1,2-bis-(2′-thienyl)disilane. *Journal of Organometallic Chemistry* **1989**, *369* (2), 155-163. DOI: https://doi.org/10.1016/0022-328X(89)88003-9.

(15) Zhang, J.; Tian, X.; Wang, Y.; Zhang, Y.; Wang, F.; Wu, L. Borane-Catalyzed Intermolecular Aryl Transfer between Hydrosilanes: Shifting the Equilibrium by Removal of a Gaseous Hydrosilane. *Journal of the American Chemical Society* **2024**, *146* (49), 33349-33358. DOI: 10.1021/jacs.4c07212.

(16) Ohshita, J.; Sugimoto, K.; Watanabe, T.; Kunai, A.; Ishikawa, M.; Aoyama, S. Polymeric organosilicon systems. XXIX. Thermal properties of poly[(disilanylene)oligophenylenes]. *Journal of Organometallic Chemistry* **1998**, *564* (1), 47-56. DOI: https://doi.org/10.1016/S0022-328X(98)00702-5.

(17) Kurpik, G.; Walczak, A.; Dydio, P.; Stefankiewicz, A. R. Multi-Stimuli-Responsive Network of Multicatalytic Reactions using a Single Palladium/Platinum Catalyst. *Angewandte Chemie International Edition* **2024**, *63* (37), e202404684. DOI: https://doi.org/10.1002/anie.202404684.

(18) Chen, S.; Guo, X.; Hou, H.; Geng, S.; Liu, Z.; He, Y.; Xue, X.-S.; Feng, Z. Thioethers as Dichotomous Electrophiles for Site-Selective Silylation via C−S Bond Cleavage. *Angewandte Chemie International Edition* **2023**, *62* (25), e202303470. DOI: https://doi.org/10.1002/anie.202303470.

(19) Jang, J.; Byun, S.; Kim, B. M.; Lee, S. Arylsilylation of aryl halides using the magnetically recyclable bimetallic Pd–Pt–Fe3O4 catalyst. *Chemical Communications* **2018**, *54* (28), 3492-3495, 10.1039/C7CC09926F. DOI: 10.1039/C7CC09926F.

(20) Neese, F. The ORCA program system. *WIREs Computational Molecular Science* **2012**, *2* (1), 73-78. DOI: https://doi.org/10.1002/wcms.81.

(21) Neese, F. Software Update: The ORCA Program System—Version 6.0. *WIREs*

Computational Molecular Science **2025**, *15* (2), e70019. DOI: https://doi.org/10.1002/wcms.70019.
(22) Grimme, S.; Hansen, A.; Ehlert, S.; Mewes, J.-M. r2SCAN-3c: A "Swiss army knife" composite electronic-structure method. *The Journal of Chemical Physics* **2021**, *154* (6). DOI: 10.1063/5.0040021 (acccessed 11/5/2024).
(23) Caldeweyher, E.; Ehlert, S.; Hansen, A.; Neugebauer, H.; Spicher, S.; Bannwarth, C.; Grimme, S. A generally applicable atomic-charge dependent London dispersion correction. *The Journal of Chemical Physics* **2019**, *150* (15). DOI: 10.1063/1.5090222 (acccessed 11/6/2024).
(24) Kruse, H.; Grimme, S. A geometrical correction for the inter- and intra-molecular basis set superposition error in Hartree-Fock and density functional theory calculations for large systems. *The Journal of Chemical Physics* **2012**, *136* (15). DOI: 10.1063/1.3700154 (acccessed 11/6/2024).
(25) Grimme, S. Supramolecular Binding Thermodynamics by Dispersion-Corrected Density Functional Theory. *Chemistry – A European Journal* **2012**, *18* (32), 9955-9964. DOI: https://doi.org/10.1002/chem.201200497.
(26) Riplinger, C.; Neese, F. An efficient and near linear scaling pair natural orbital based local coupled cluster method. *The Journal of Chemical Physics* **2013**, *138* (3). DOI: 10.1063/1.4773581 (acccessed 3/23/2025).
(27) Riplinger, C.; Sandhoefer, B.; Hansen, A.; Neese, F. Natural triple excitations in local coupled cluster calculations with pair natural orbitals. *The Journal of Chemical Physics* **2013**, *139* (13). DOI: 10.1063/1.4821834 (acccessed 3/23/2025).
(28) Marenich, A. V.; Cramer, C. J.; Truhlar, D. G. Universal Solvation Model Based on Solute Electron Density and on a Continuum Model of the Solvent Defined by the Bulk Dielectric Constant and Atomic Surface Tensions. *The Journal of Physical Chemistry B* **2009**, *113* (18), 6378-6396. DOI: 10.1021/jp810292n.
(29) Zhao, Y.; Truhlar, D. G. The M06 suite of density functionals for main group thermochemistry, thermochemical kinetics, noncovalent interactions, excited states, and transition elements: two new functionals and systematic testing of four M06-class functionals and 12 other functionals. *Theoretical Chemistry Accounts* **2008**, *120* (1), 215-241. DOI: 10.1007/s00214-007-0310-x.
